# Randomized phase I trial of antigen-specific tolerizing immunotherapy with peptide/calcitriol liposomes in ACPA^+^ rheumatoid arthritis

**DOI:** 10.1172/jci.insight.160964

**Published:** 2022-10-24

**Authors:** Amee Sonigra, Hendrik J. Nel, Pascale Wehr, Nishta Ramnoruth, Swati Patel, Karin A. van Schie, Maxwell W. Bladen, Ahmed M. Mehdi, Joanne Tesiram, Meghna Talekar, Jamie Rossjohn, Hugh H. Reid, Frederik E. Stuurman, Helen Roberts, Phillip Vecchio, Ian Gourley, Mark Rigby, Stephane Becart, Rene E.M. Toes, Hans Ulrich Scherer, Kim-Anh Lê Cao, Kim Campbell, Ranjeny Thomas

**Affiliations:** 1 Department of Rheumatology, Princess Alexandra Hospital, Brisbane, Queensland, Australia.; 2 University of Queensland Diamantina Institute, the University of Queensland, Woolloongabba, Queensland, Australia.; 3 Department of Rheumatology, Leiden University Medical Center, Leiden, Netherlands.; 4 Melbourne Integrative Genomics and School of Mathematics and Statistics, University of Melbourne, Melbourne, Victoria, Australia.; 5 Department of Biochemistry and Molecular Biology, School of Biomedical Sciences, Monash Biomedicine Discovery Institute, Monash University, Clayton, Victoria, Australia.; 6 Division of Infection and Immunity, School of Medicine, Cardiff University, Cardiff, United Kingdom.; 7 Dendright Pty Ltd, Brisbane, Queensland, Australia.; 8 Immunology Clinical Development, Janssen Research & Development, LLC, Spring House, Pennsylvania, USA.; 9 Discovery Immunology, Janssen Research & Development, LLC, La Jolla, California, USA.; 10 Immunology Translational Medicine, Janssen Research & Development, LLC, Spring House, Pennsylvania, USA.

**Keywords:** Autoimmunity, Clinical Trials, Dendritic cells, Rheumatology, Tolerance

## Abstract

**BACKGROUND:**

Antigen-specific regulation of autoimmune disease is a major goal. In seropositive rheumatoid arthritis (RA), T cell help to autoreactive B cells matures the citrullinated (Cit) antigen-specific immune response, generating RA-specific V domain glycosylated anti-Cit protein antibodies (ACPA VDG) before arthritis onset. Low or escalating antigen administration under “sub-immunogenic” conditions favors tolerance. We explored safety, pharmacokinetics, and immunological and clinical effects of s.c. DEN-181, comprising liposomes encapsulating self-peptide collagen II_259-273_ (CII) and NF-κB inhibitor 1,25-dihydroxycholecalciferol.

**METHODS:**

A double-blind, placebo-controlled, exploratory, single-ascending-dose, phase I trial assessed the impact of low, medium, and high DEN-181 doses on peripheral blood CII-specific and bystander Cit64vimentin_59-71_–specific (Cit-Vim–specific) autoreactive T cell responses, cytokines, and ACPA in 17 HLA-DRB1*04:01^+^ or *01:01^+^ ACPA^+^ RA patients on methotrexate.

**RESULTS:**

DEN-181 was well tolerated. Relative to placebo and normalized to baseline values, Cit-Vim–specific T cells decreased in patients administered medium and high doses of DEN-181. Relative to placebo, percentage of CII-specific programmed cell death 1^+^ T cells increased within 28 days of DEN-181. Exploratory analysis in DEN-181–treated patients suggested improved RA disease activity was associated with expansion of CII-specific and Cit-Vim–specific T cells; reduction in ACPA VDG, memory B cells, and inflammatory myeloid populations; and enrichment in CCR7^+^ and naive T cells. Single-cell sequencing identified T cell transcripts associated with tolerogenic TCR signaling and exhaustion after low or medium doses of DEN-181.

**CONCLUSION:**

The safety and immunomodulatory activity of low/medium DEN-181 doses provide rationale to further assess antigen-specific immunomodulatory therapy in ACPA^+^ RA.

**TRIAL REGISTRATION:**

Anzctr.org.au identifier ACTRN12617001482358, updated September 8, 2022.

**FUNDING:**

Innovative Medicines Initiative 2 Joint Undertaking (grant agreement 777357), supported by European Union’s Horizon 2020 research and innovation programme and European Federation of Pharmaceutical Industries and Associations; Arthritis Queensland; National Health and Medical Research Council (NHMRC) Senior Research Fellowship; and NHMRC grant 2008287.

## Graphical abstract

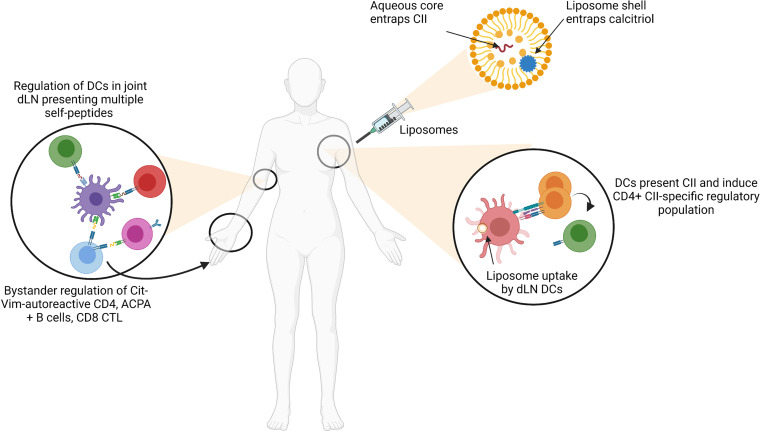

## Introduction

Rheumatoid arthritis (RA) is an inflammatory autoimmune disease affecting 23 million people worldwide (1). The incidence of RA is disproportionately high in the female sex; risk is increased by family history and lifestyle factors, including smoking and obesity. RA erodes quality of life and requires lifelong treatment with expensive, noncurative, antiinflammatory drugs (2). Ongoing disability and premature mortality, due to chronic joint and cardiovascular inflammation, occur because current drugs only partially block symptoms, cause unwanted side effects, and fundamentally do not target (or cure) the underlying immune mechanism (3). A paradigm shift in treatment of RA is needed.

Antigen-specific immunological tolerance (ASIT) strategies leverage the natural process of antigen presentation by dendritic cells (DCs) to regulate pathogenic T cells and antigen-presenting cells (APCs). DCs are professional APCs that migrate from peripheral tissues to draining lymph nodes (dLNs), where they present antigens in an HLA-restricted manner to T cells (4). In preclinical models of ASIT, peripheral antigen-specific regulatory T cells (Tregs) required T cell receptor (TCR) ligation and were shown to suppress multiple autoreactive T cell specificities through bystander tolerance (5, 6). Other models showed that repeated “sub-immunogenic” low doses and dose escalation of TCR agonists favored persistence of Foxp3^+^ Tregs and differentiation of Foxp3^–^ type 1 Treg (Tr1) cells (7–10). In autoimmune or autoallergic conditions, various ASIT approaches deliver antigen in a context that reprograms DC function through modulation of NF-κB or by targeting steady-state DCs or a naturally tolerogenic environment to promote autoreactive T cell regulation (11). When autologous DCs modified with an NF-κB inhibitory drug were exposed to citrullinated (Cit) peptides and administered to HLA risk genotype–positive RA patients in a proof-of-concept phase I clinical trial, the ASIT was safe, regulated inflammation, and modulated CD4^+^ effector T cells (12). To expand on these findings, we developed a nanoparticulate liposome formulation that encapsulates the RA joint-specific, HLA-DRB1*04:01- and *01:01-restricted native self-peptide collagen II_259-273_ (CII) with the NF-κB inhibitor 1,25 dihydroxycholecalciferol (calcitriol), which targets dLN DCs. In mouse models of autoimmune disease, multiple doses of liposomes expanded programmed cell death 1^+^ (PD-1^+^) antigen-specific T cells, induced antigen-specific Foxp3^+^ Tregs in a programmed cell death ligand 1–dependent (PD-L1–dependent) manner, regulated memory T (Tmem) cells, reduced disease severity, and induced bystander tolerance (5, 13).

ASIT strategies face several translational hurdles to realize clinical benefit, including difficulties in translating preclinical models to clinical trials and a general lack of robust biomarkers of antigen-specific responding cells for evidence of regulation. However, small, exploratory, phase 0 and phase I clinical trials afford the opportunity to test novel immunotherapeutic drugs for safety while undertaking a broad immunological analysis, including novel assays of antigen-specific and bystander T cells optimized and evaluated for precision and reproducibility (14). In this regard, various regulatory immune signatures have emerged from the analysis of patients with type 1 diabetes (T1D), multiple sclerosis, or allogeneic hematopoietic stem cell transplantation, who achieve prolonged partial remission (from T1D) or a favorable response to T cell immunotherapies. The favorable responses include CD4^+^CD25^+^CD127^hi^ Th cells producing Th2 cytokines, IL-10–producing CD4^+^ T cells, CD4^+^ proinsulin-specific T cell proliferation, and EOMES^+^TIGIT^+^KLRG1^+^ “partially exhausted” CD8^+^ T cells (15–19). In RA, T cells recognizing Cit and other self-antigens, such as native and posttranslationally modified CII_259-273_, are identifiable with peptide-HLA-DR tetramers and are found in low numbers in peripheral blood and synovial fluid of patients with HLA-DRB1*04:01 and *01:01 (20–25). Due to their “immune-aged” phenotype, RA T cells may undergo replication stress and a propensity to die by pyroptosis in response to TCR activation, which may contribute to observed low frequencies of autoreactive T cells in synovial fluid (26, 27). Predisposition to poor vaccine responses due to T cell replication stress (28) could also affect the capacity of T cells to respond to tolerizing immunotherapy.

We carried out a dose-ranging, double-blind, placebo-controlled phase I clinical trial of a single ascending dose of liposomes encapsulating 40 μg/mL CII + 400 ng/mL calcitriol (DEN-181) in 17 anti-citrullinated protein antibody^+^ (ACPA^+^) HLA-DRB1*0401 or *0101^+^ RA patients on methotrexate. The primary objective was safety and tolerability, and the secondary objectives were antigen-specific and bystander T cell immunomodulation, preliminary clinical efficacy, and plasma calcitriol concentrations after DEN-181.

## Results

### Patients.

We prescreened 56 ACPA^+^ RA patients on methotrexate (MTX) for HLA-DR and identified 26 who carried HLA-DRB1*04:01 or *01:01. After screening for trial eligibility, 17 were included in the study and assigned to 1 of 3 cohorts (1 mL volume, 42 μg CII, 0.6 μg calcitriol; 0.3 mL volume, 12.6 μg CII, 0.18 μg calcitriol; or 3 mL volume, 126 μg CII, 1.8 μg calcitriol), as outlined in the flow chart (Supplemental Figure 1; supplemental material available online with this article; https://doi.org/10.1172/jci.insight.160964DS1). As MTX monotherapy was an eligibility criterion, some patients withdrew from combination disease-modifying antirheumatic drugs to meet eligibility, resulting in changes in disease activity and MTX dose (Supplemental Table 1). Screen failures included patients whose dose of MTX was not stable for 4 weeks, whose ACPA level was below the cutoff, and who decided not to participate for other reasons. Baseline characteristics and number of participants in each group are shown in Table 1. Patient disease duration ranged from 0.5 to 13 years. The median disease duration was significantly shorter (1.3 years) in the 0.3 mL cohort than other cohorts. Median baseline DAS28CRP ranged 2.2–3.1 and was comparable across cohorts. Median MTX dose ranged 16.3–18.3 mg/week and was comparable across cohorts.

### Safety.

Participants were administered DEN-181 in a single s.c. site (1 mL volume in left upper arm), 2 sites (150 μL volume in each upper arm), or 3 sites (1 mL volume in each upper arm and upper thigh). Potentially treatment-associated adverse events included grade 1 elevation of aspartate transaminase (AST) or alanine transaminase (ALT) (1 each in 3 mL and placebo cohorts), injection site bruising (1 each in 1 mL, 3 mL, and placebo cohorts), and grade 2 joint synovitis (1 each in 3 mL and placebo cohorts, Table 2). These RA flares were treated with prednisone until the end of the study. One participant receiving 3 mL had a lower respiratory tract infection on the day of dosing and was treated 1 day later with oral amoxicillin (Supplemental Table 1). One participant in cohort 2 experienced a serious but unrelated adverse event after screening and before dosing: acute lumbar spine back pain for 12 days, which was treated with oxycodone, naloxone, and pregabalin. There were no significant differences for other laboratory data between cohorts.

### Pharmacokinetics of plasma calcitriol after s.c. DEN-181.

**Figure 1:**
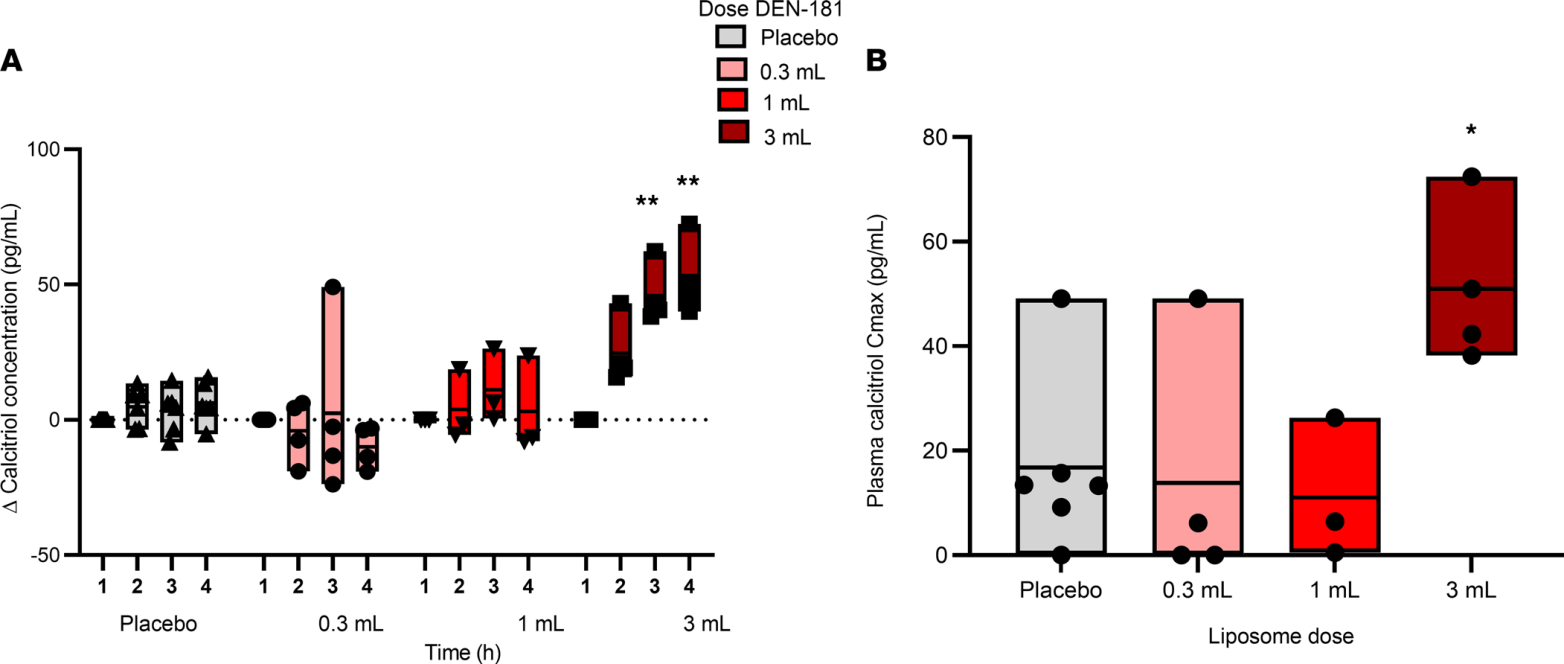
Plasma calcitriol pharmacokinetics. The change in plasma calcitriol concentration from baseline for the placebo (*n* = 6), 0.3 mL (*n* = 4), 1 mL (*n* = 3), and 3 mL (*n* = 4) dose groups, over the first 4 hours after dosing (**A**) and the C_max_ plasma calcitriol; **P < 0.01 compared with placebo (2-way ANOVA with Dunnett’s multiple comparisons correction) (**B**). Each box represents the range, with each individual plotted (min-max); *P = 0.0298, compared with placebo (1-way ANOVA, Dunnett’s multiple-comparison test).

Preclinical studies of DEN-181 in mice demonstrated that after s.c. injection, a proportion of the administered dose rapidly dispersed via afferent lymphatics to dLNs, where liposomes are taken up by skin migratory DCs (13). In biodistribution studies of liposomes encapsulating ^3^[H] calcitriol and ^14^[C]phospholipids, most radioactivity attributable to ^3^[H]calcitriol was retained at the injection site. However, small amounts of calcitriol entered the bloodstream within 30 minutes of dosing and more rapidly than the liposomes, which were found in blood and spleen at 2–24 hours (13). To assess calcitriol in humans, we measured plasma calcitriol 1, 2, and 4 hours after dosing in all participants. Plasma calcitriol increased significantly over the time course only in the 3 mL cohort, relative to placebo, and was associated with a significantly increased C_max_ (Figure 1). These data indicate that administered calcitriol does not significantly increase the endogenous levels in the circulation except after a 3 mL dose, where calcitriol could be free or liposome associated.

### Immunomodulatory effects of DEN-181.

**Figure 2:**
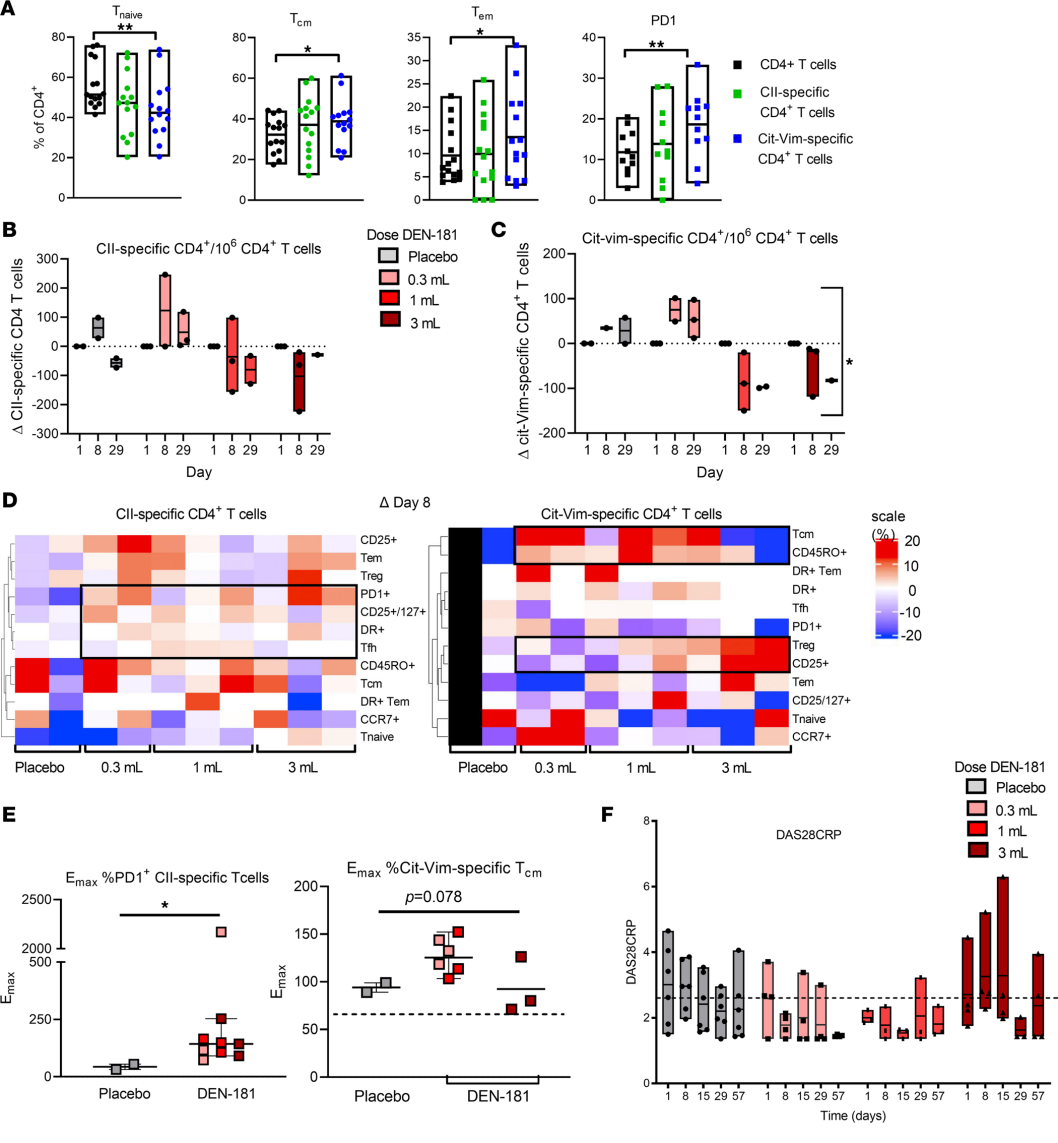
Antigen-specific T cells and clinical response of dose groups. (**A**) The percentage of naive, central memory (Tcm), effector memory (Tem), and PD-1^+^ CII-specific, Cit-Vim–specific, and CD4^+^ T cells at baseline in all trial participants (*n* = 17), analyzed by flow cytometry. Each box represents the range (min-max). *P < 0.05, **P < 0.01 compared with placebo (linear mixed effects model with Tukey’s multiple comparisons correction). The change in number of CII-specific (**B**) and Cit-Vim–specific T cells (**C**) per 10^6^ CD4^+^ T cells, relative to day 1, plotted for individuals across dose groups at day 8 and day 29. CII-specific and Cit-Vim–specific T cells were identified by flow cytometry using HLA-DRB1*04:01 and *01:01-CII and HLA-DRB1*04:01 and *01:01-64Cit-Vim tetramers. Gray bars: placebo, pink bars: 0.3 mL, red bars: 1 mL, dark red bars: 3 mL DEN-181 (*n* = 11). *P* = 0.0002 for effect of dose and *P* = 0.0212 for dose × time (linear mixed effects model to assess time and dose, with Geisser-Greenhouse correction) for Cit-Vim–specific T cells. (**D**) The change in prespecified phenotypic subset proportion in CII-specific, Cit-Vim–specific CD4^+^ T cells relative to day 1, represented as a heatmap for individuals across dose groups at day 8. Scale +20 to –20%. All data points are shown. (**E**) The maximum increase in %PD-1^+^ CII-specific CD4^+^ T cells was calculated in each participant relative to baseline (i.e., E_max_ = peak value/baseline value × 100%). E_max_ %PD-1^+^ CII-specific T cells comparing placebo and combined DEN-181–treated participants, plotted with median and IQR. *P = 0.0364 Mann-Whitney test. (**F**) DAS28CRP plotted for individuals across dose groups at day 1, 8, 15, 29, and 57 (*n* = 17). Each box represents the range (min-max), including all data points. *P* = 0.0258 for effect of time (linear mixed effects model to assess time and dose, with Geisser-Greenhouse correction). Patients with DAS28CRP < 2.6 (dotted line) are in DAS remission.

To assess T cell immunomodulation, we evaluated total CD4^+^ and CD8^+^ T cells, antigen-specific CD4^+^ T cells responsive to CII administered in DEN-181, and bystander CD4^+^ T cells recognizing Cit64vimentin_59-71_ (Cit-Vim), a well-described autoantigenic epitope restricted by HLA-DRB1*04:01 and *01:01 (23, 29, 30). Antigen-specific T cells were enumerated at baseline (day 1), day 8, and day 29, with haplotype-specific tetramers in a fit-for-purpose flow cytometric assay developed to monitor antigen-specific functional CD4^+^ T cell subpopulations, with high precision and reproducibility (Supplemental Figure 2) (25). Stored, frozen PBMCs from 11 participants (2 placebo and 3 from each of the DEN-181 dose cohorts) were available for retrospective analysis using this assay. Across all participants at baseline, there was considerable interindividual variability in the proportions of effector memory T (Tem) cells, central memory T (Tcm) cells, and PD-1 expression across CD4^+^, CII-specific, and Cit-Vim–specific T cells. However, Cit-Vim–specific T cells were significantly enriched in Tem cells, Tcm cells, and PD-1 expression compared with total CD4^+^ T cells (Figure 2A). Due to the small numbers of participants in each group and inherent individual variability, we corrected longitudinal changes to each individual’s baseline, as for a previous analysis (12). In the 28 days after dosing relative to day 1, the number of CII-specific CD4^+^ T cells did not change significantly. The trend was toward decrease in the cohorts receiving 1 mL and 3 mL, compared with baseline, while CII-specific CD4^+^ T cells were stable or increased in the cohort receiving 0.3 mL (Figure 2B and Supplemental Figure 3A). The number of Cit-Vim–specific CD4^+^ T cells significantly differed by dose and dose over time (linear mixed model analysis). The trends for Cit-Vim–specific CD4^+^ T cells were toward increase in the 0.3 mL cohort and decrease in the cohorts receiving 1 mL and 3 mL at days 8 and 29 (Figure 2C and Supplemental Figure 3). The number of CD4^+^ T cells did not change significantly relative to baseline (Supplemental Figure 3A). These data suggest a DEN-181 dose-related impact on the number of circulating Cit-Vim–specific T cells with a similar trend for CII-specific T cells.

To explore whether the phenotype of the antigen-specific and bystander T cells changed, we carried out a clustering analysis of the changes in percentage of CII-specific and Cit-Vim–specific T cell subsets at day 8 as the most proximal time point to DEN-181 dosing relative to baseline. Compared with placebo, CII-specific T cells appeared recently activated, with a greater percentage expressing PD-1, CD25/CD127, HLA-DR, or Tfh markers with any dose of DEN-181 (Figure 2D). Because PD-1 was a key regulatory marker in preclinical studies (13), and the timing of the peak change in percentage of PD-1^+^ CII-specific T cells varied between 8 and 29 days, we calculated the peak percentage increase (E_max_ = peak value/baseline value × 100%) above baseline. Percentage of PD-1^+^ CII-specific T cell E_max_ was significantly greater in DEN-181–treated than placebo-treated participants (Figure 2E). The Cit-Vim–specific T cells tended to acquire more Tcm markers after lower doses of DEN-181 (Figure 2D). Percentage of Cit-Vim–specific Tcm E_max_ was highest in participants treated with 0.3 mL or 1 mL DEN-181 (*P* = 0.077, Figure 2E). Changes in these subsets were not apparent among total CD4^+^ or CD8^+^ T cells of DEN-181–treated relative to placebo-treated participants (Supplemental Figure 3B).

RA disease activity scores (DAS28CRP) changed significantly over time (*P* < 0.05, 2-way ANOVA). All patients in the 0.3 mL and 1 mL cohorts had a DAS28CRP < 2.6 (remission) on day 57. DAS28CRP transiently increased over the first 15 days in the 3 mL cohort (Figure 2F and Supplemental Figure 3C).

### Serum ACPA, ACPA variable domain glycosylation, cytokines, and chemokines.

**Figure 3:**
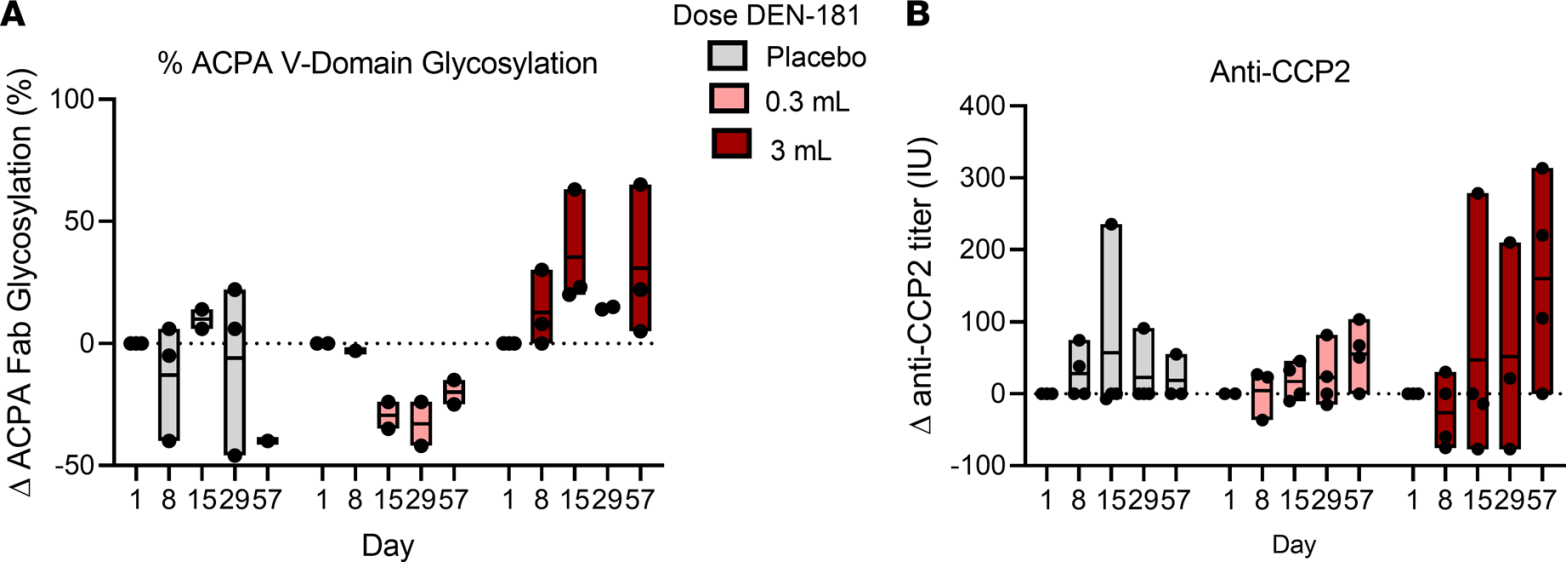
Serum anti-citrullinated protein antibodies. The change in percentage anti-citrullinated protein antibody (ACPA) glycosylation (**A**) and anti-CCP2 titre (**B**) at day 8, 15, 29, and 57 relative to day 1, plotted for individuals across placebo and 0.3 mL and 3 mL (*n* = 10) dose groups. Anti-CCP2 was measured by ELISA and Fab glycosylation by HPLC. Each box represents the range (min-max), including all data points.

While the ACPA V domain glycosylation (VDG) varied over time in the placebo group, we observed a trend of reduction in ACPA VDG from day 15 to day 57 in participants treated with 0.3 mL DEN-181 with an opposite trend in participants treated with 3 mL DEN-181 (Figure 3A). Fc ACPA percentage fucosylation, galactosylation, and sialylation did not change in any group over time, while percentage of bisection decreased at day 29 after 0.3 mL DEN-181, relative to participants receiving placebo or 3 mL (Supplemental Figure 4A). ACPA IgG levels (CCP2 ELISA) did not change significantly after treatment, but the trend was toward an increase after 3 mL DEN-181 (Figure 3B).

We analyzed 12 serum cytokines and chemokines relevant to the immune response and inflammation (Supplemental Table 3) at days 1, 29, and 57 in participants treated with placebo, 0.3 mL, or 3 mL DEN-181. There was a downward trend in serum amyloid A (SAA) at day 57 in the 3 mL cohort, potentially driven by the participant treated with steroid (Supplemental Figure 4B).

### DCs, monocytes, and B cells.

To assess APCs, we analyzed B cell, monocyte, and DC subset numbers by flow cytometry at days 1, 8, and 29 in all participants. Relative to placebo, there were no significant changes in cell numbers after treatment with DEN-181 (Supplemental Figure 4C).

### Effects of treatment.

**Figure 4:**
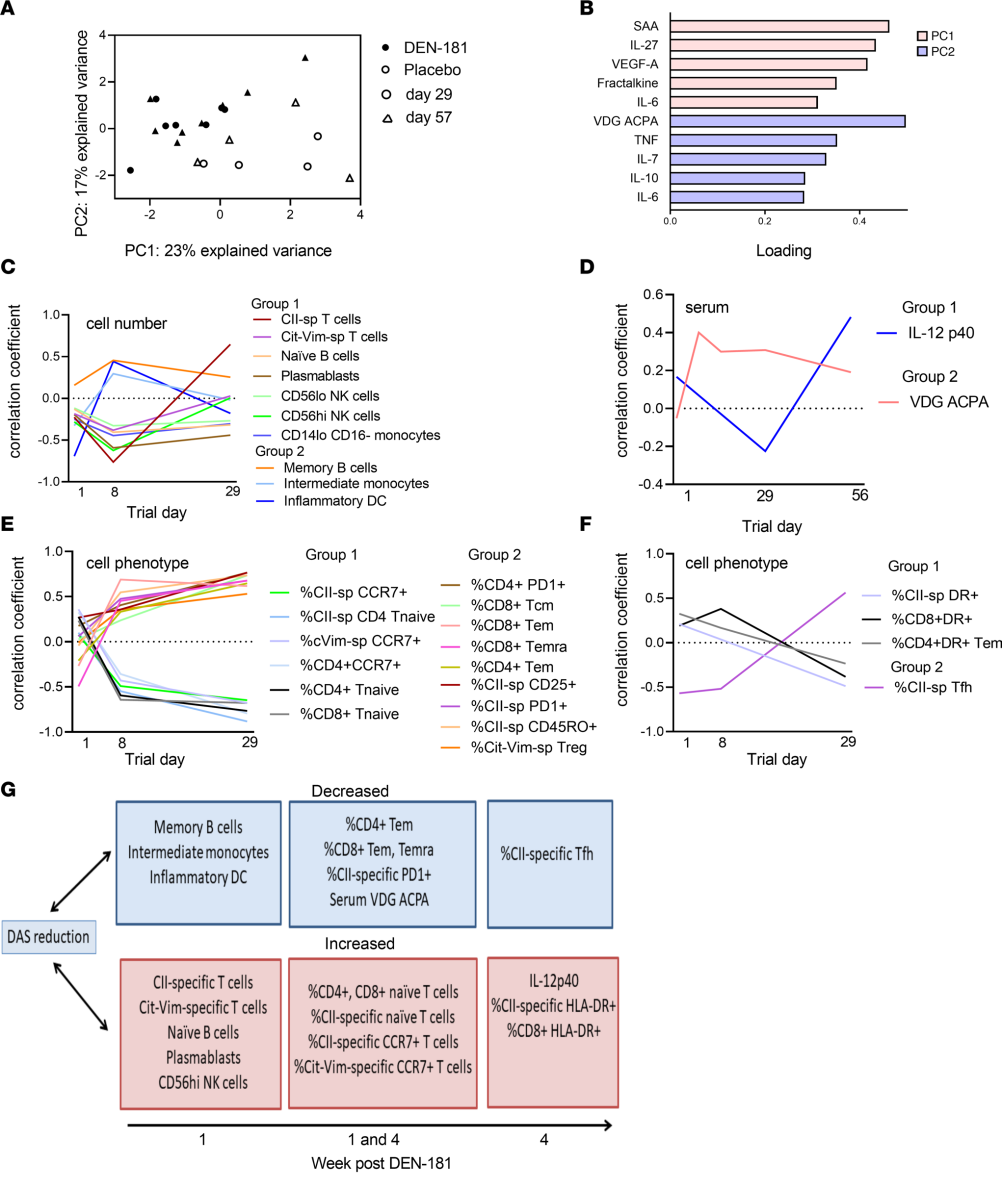
Associations with treatment and with disease activity in treated participants. Principal component analysis (PCA) was used to identify major sources of variation in DEN-181–treated and placebo-treated participants at each collection time point. Samples from serum data were projected onto the first 2 principal components (**A**). Symbols depict the day of data collection, and colors depict DEN-181 or placebo groups. (**B**) Loading coefficients of the top 5 features important for the first and second components of the PCA (which explain the variation between treatment and days). After correlating cell count, phenotype, and serum features with DAS28CRP over time in DEN-181–treated participants, K-medoid clustering identified similar time-associated patterns of correlation with DAS28CRP. (**C**) Cell number: group 1 (group 2) features with stronger negative (positive) correlations at day 8. (**D**) Serum: group 1 (group 2) features with stronger negative (positive) correlations at day 29 (day 8–56). (**E**) Cell phenotype: group 1 (group 2) features with stronger negative (positive) correlation at day 8 and 29. (**F**) Cell phenotype: group 1 (group 2) features with stronger positive (negative) correlation becoming negative (positive) at day 29. (**G**) Schema summarizing key DAS28CRP correlates.

We used principal component analysis to identify treatment-associated immunological impacts. Serum cytokines, including SAA, IL-27, VEGF-A, fractalkine, IL-6, TNF, IL-7, IL-10, and ACPA VDG best explained the variance between DEN-181– and placebo-treated participants at day 29 and 57 (Figure 4, A and B).

### Correlations with disease activity.

To further explore how immunological features potentially related to immunotherapy were associated with clinical outcome, we calculated the correlation of each of the features with DAS28CRP in DEN-181–treated participants across all time points, then clustered features to identify similar changes over time based on their correlation coefficients. After DEN-181 treatment, a reduction in DAS28CRP correlated at day 8 with increases in the number of total CII- and Cit-Vim–specific T cells, naive B cells, plasmablasts, CD56^hi^ and CD56^lo^ NK cells, and CD14^lo^CD16^–^ monocytes and with decreases in memory B cells, intermediate monocytes, and CD14^+^CD1c^+^ inflammatory DCs (Figure 4C). A reduction in DAS28CRP correlated at day 29 with an increase in IL-12p40. A reduction in DAS28CRP correlated at days 15–56 with a decrease in VDG ACPA in DEN-181–treated participants (Figure 4D). A reduction in DAS28CRP correlated at day 8 and 29 with an increase in the percentage of CII-specific, CD4^+^, and CD8^+^ T cells with naive phenotype and of CII-specific, Cit-Vim–specific, and CD4^+^ T cells expressing CCR7 and a decrease in percentages of CII-specific T cells expressing CD25, PD-1, CD45RO, Cit-Vim–specific CD25^+^CD127^–^ T cells, CD4^+^PD-1^+^ T cells, and CD8^+^ Tcm cells (Figure 4E). A reduction in DAS28CRP correlated at day 29 with an increase in the percentage of CII-specific (Figure 4F), CD4^+^ Tem cells and CD8^+^ T cells expressing HLA-DR and a decrease in CII-specific follicular helper T (Tfh) cells. This exploratory analysis suggests that multiple cells were modulated over time as disease activity changed in patients treated with DEN-181. In summary, improved disease activity was associated with early expansion of CII-specific and Cit-Vim–specific T cells; reduction in ACPA VDG, memory B cells, and inflammatory myeloid populations; and sustained enrichment in total CII-specific and Cit-Vim–specific CCR7^+^ and naive T cells. Improved disease activity was also associated with increased HLA-DR^+^ CII-specific and CD8^+^ T cells and serum IL-12p40 at day 29 (Figure 4G), suggesting ongoing antigen-specific and bystander immune responses.

### Exploratory single-cell transcriptomic and TCR clonotypic analysis.

**Figure 5:**
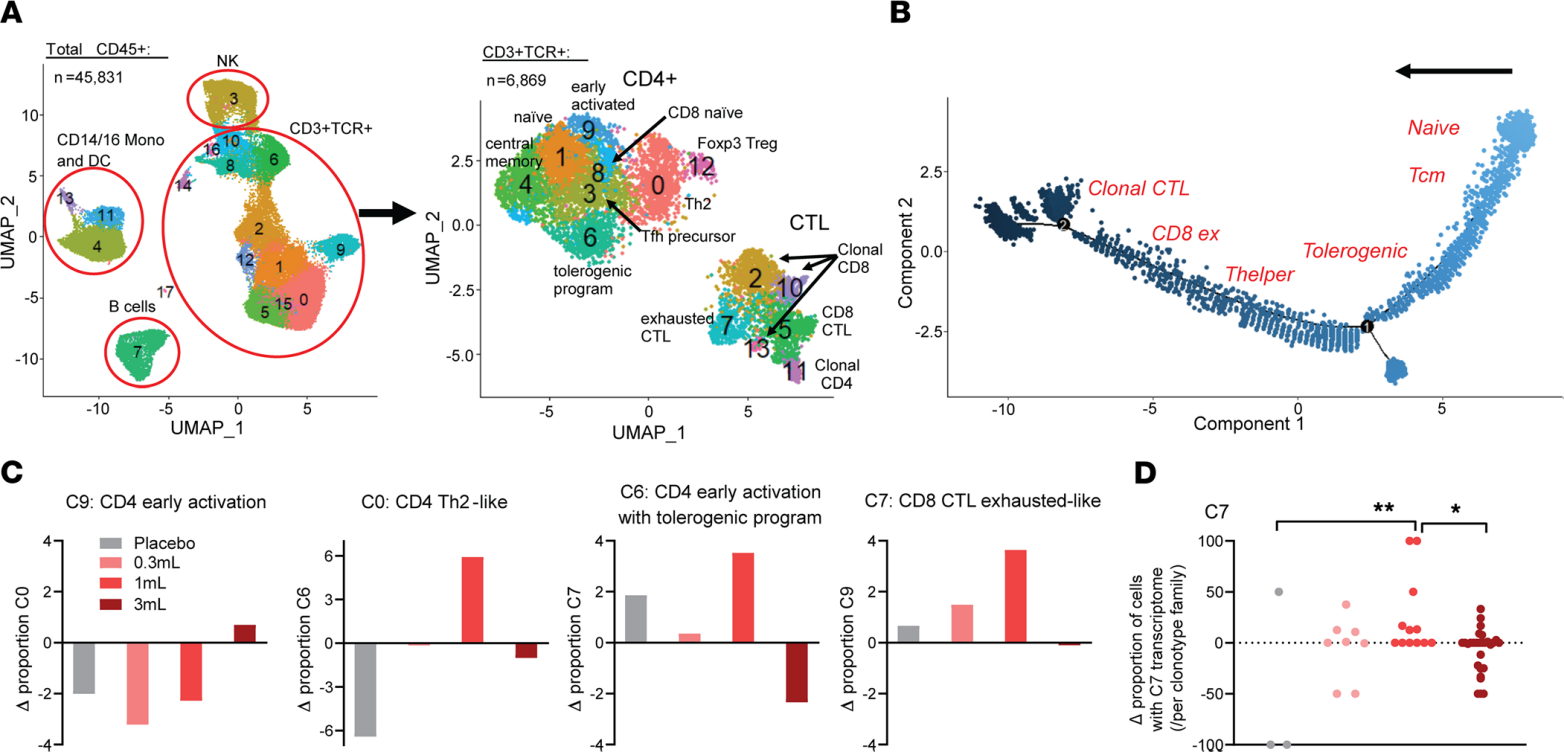
Single-cell TCR/RNA-Seq demonstrates expanded CD4 tolerogenic program and CD8 exhaustion of persistent clonotypes 28 days after 1 mL DEN-181. (**A**) Uniform manifold approximation and projection (UMAP) of all 45,831 CD45^+^ cells from PBMCs from all patients at day 1 and day 29 pooled together, with supercluster annotations assigned by canonical marker gene expression. The CD3^+^TCR^+^ supercluster was reclustered, resulting in the UMAP comprising 6,869 cells on the right, which was manually annotated (Methods, Supplemental Figure 7). (**B**) Pseudotime trajectory analysis using Monocle 2. Manual annotation depicts the approximate position of enrichment of each Seurat cluster on the trajectory. (**C**) The change in specified CD3^+^TCR^+^ subclusters at day 29 relative to day 1 for the placebo, 0.3 mL, 1 mL, and 3 mL dose groups (*n* = 4). (**D**) The change in proportion of persistent clonotypes with C7 transcriptome at day 29 relative to day 1 (*n* = 3–19 C7 clonotypes; 1-way ANOVA with Holm-Šídák multiple comparisons test, *P < 0.05, **P < 0.01).

We further explored the impact of DEN-181 dosing with single-cell RNA-sequencing analysis of the available PBMC samples (*n* = 1 per dose level, including placebo) at day 1 and day 29. All had a fall in DAS28CRP at day 29 compared with day 1, albeit with different trajectories (Supplemental Figure 5). After sorting CD45^+^ leukocytes, we obtained single-cell transcriptomes and TCR information using the 10x 5′ Genomics Chromium platform. With demultiplexing and sample-specific stringent filtering to obtain high-quality cells (Supplemental Table 4), 45,831 single-cell transcriptomes were obtained. Subsequent clustering resulted in 17 distinct cell clusters based on signature marker expression, corresponding to 4 superclusters; CD14/CD16^+^ monocytes and DCs, NK cells, B cells, and T cells (Figure 5A and Supplemental Figure 6). All superclusters contained cells from all patients and from pre- and posttreatment time points (Supplemental Figure 6C).

We focused on T cells and preexisting expanded TCR clonotypes — defined as clonotypes found at both day 1 and day 29 and having a CDR3αβ count greater than 1 at either of the time points (Supplemental Figure 8A). The vast majority of TCR^+^ cells were identified among cells expressing *CD3D*, and TCR expression was minimal in non–T cell clusters (Supplemental Figure 6B). We subclustered cells coexpressing *CD3D* and a productive, paired TCRα/β to obtain higher resolution T cell clonotype information (Supplemental Figure 6B). With unsupervised reclustering of CD3^+^TCR^+^ cells, 14 clusters were identified, which separated into 2 superclusters based on differentially expressed genes (DEGs) — one (predominantly CD4^+^) underexpressing and the second (predominantly CD8^+^) overexpressing cytotoxicity genes, such as *GZMB*, *GZMK*, *GNLY*, and *NKG7* (Figure 5A, Supplemental Table 5, and Supplemental Figure 7A). Based on *TRBV* expression, cluster (C) 2, C10, and C13 were clonal CD8^+^ cytotoxic T lymphocyte–like (CTL-like) and C11 clonal CD4^+^ CTL-like clusters, mostly specific to the participant dosed with 3 mL DEN-181 (disease duration 19 years). We identified 9 additional CD3^+^TCR^+^ clusters, including activated and exhausted CD8^+^ CTL-like, naive, Th2-like, memory, and CD4^+^ Treg clusters (Figure 5A and Supplemental Figure 7, B–D). Clonal CTL was the most differentiated subpopulation, based on trajectory analysis (Figure 5B).

CD4^+^ T cells (C9) expressing early activation genes, e.g., *JUNB*, *CD69*, and amphiregulin (*AREG*), increased, and Th2-like CD4^+^ T cells (C0) expressing *GATA3* and *ANXA1* decreased in the participants who received 0.3 mL and 1 mL DEN-181. After the 1 mL dose, C6 (CD4^+^ T cells with a tolerance and TCR signaling–associated transcriptomic program including *NR4A1* [Nur77], *JUN*, *ICOS*, *AREG*, and genes signifying cell cycling), and C7 (exhausted-like CTLs expressing *NR4A1*, *KLRG1*) increased (Figure 5C; Supplemental Figure 7, D and E; and Supplemental Table 5). Among expanded CTL clonotypes present at day 1, the proportion of exhausted-like cells per clonotype family increased 28 days after 1 mL DEN-181 relative to placebo (*P* = 0.0055) or 3 mL DEN-181 (*P* = 0.0328) (Figure 5D). We noted no other changes to clonotype transcriptomes when compared to placebo (Supplemental Figure 8). These data further support the hypothesis that 0.3 mL and 1 mL of DEN-181 triggered the TCR, with multiple immunomodulatory impacts.

## Discussion

ASIT with relevant self-antigens, individualized to the patient’s genetic background and disease, aims to induce prolonged immunoregulation of autoimmunity and improved outcomes, with few adverse events. However, few such approaches have been translated into clinical trials in autoimmune disease, and there is a general lack of biomarkers to assess immunoregulatory outcomes. As a first step, in this first-in-human phase I study, we wished to explore feasibility, safety, and immunological and clinical effects of a single low, medium, or high dose of CII/calcitriol liposomes in MTX-treated HLA-DRB1*04:01/01:01^+^ RA patients, using a fit-for-purpose flow cytometric assay of antigen-specific T cells previously evaluated for reproducibility (25). From the numbers of participants, the data can be considered mechanism or hypothesis generating, in relation to future studies.

### Safety, tolerability, feasibility, and pharmacokinetics.

Recruitment of ACPA^+^ RA patients with specific HLA-DR genotypes was feasible. A single s.c. administration of 0.3, 1, or 3 mL DEN-181 (12.6 μg, 42 μg, 126 μg CII peptide) was generally well tolerated. A dose of 1 mL DEN-181 was chosen by extrapolating the mouse dose of 0.1 mL to humans based on the estimated difference in lymph node volume. Thus, the administered dose ranged from one-third to 3-fold the estimated tolerogenic dose of coencapsulated peptide and calcitriol. One participant in each of the placebo and 3 mL groups flared. Both participants who flared commenced the trial with a high DAS28CRP. Calcitriol increased in plasma for 4 hours after a dose of 3 mL DEN-181. There were no symptoms associated with this acute increase in plasma calcitriol in participants receiving 3 mL DEN-181. Similar 2-hour increases in plasma liposome-associated and free calcitriol, which decreased by 24 hours, were observed in mice (25).

Previous clinical trials of peptide immunotherapy found that 10 μg C19-A3 proinsulin peptide s.c. every 2 weeks was well tolerated in patients with recent-onset T1D (31), while 60–150 μg weekly s.c. administration of celiac disease–relevant peptides induced dose-dependent gastrointestinal symptoms associated with T cell IL-2 production (32–34). Similarly, proinflammatory adverse effects of anti-CD3 immunotherapy in T1D were dose dependent (35). Taken together, we hypothesize that transient increases in joint symptoms after a 3 mL dose correspond to the strength of TCR signaling.

### Immunoregulatory and clinical effects.

Relative to placebo and normalized to baseline values, Cit-Vim–specific T cells decreased in patients administered medium and high doses of DEN-181. Relative to placebo, percentage of CII-specific PD-1^+^ T cells increased within 28 days of DEN-181 dosing. Exploratory analysis in DEN-181–treated patients found that improved RA disease activity correlated with expansion of CII-specific and Cit-Vim–specific T cells; reduction in ACPA VDG, memory B cells, and inflammatory myeloid populations; and enrichment in proportions of CCR7^+^ and naive T cells in peripheral blood (PB). We identified T cell transcripts associated with tolerogenic TCR signaling and exhaustion, including *JUNB*, *CD69*, *AREG*, *NR4A1*, and *KLRG1* after low or medium doses of DEN-181.

These data support the feasibility of mechanistic analysis of immunotherapy clinical trials with a fit-for-purpose tetramer flow cytometry assay (25). DEN-181 delivers native CII_259-273_ to the peripheral immune system. Upregulation of the PD-1 marker of antigen experience by CII-specific T cells, changes in Cit-Vim–specific T cell numbers, and expansion of Nur77 and exhaustion signatures together suggest effects of a single dose of DEN-181 on the peripheral CII-specific and bystander T cell immune response. Correlation of CCR7^+^ T cells with DAS28CRP reduction is of interest, as CCR7 reflects lymph node trafficking potential where interaction with APCs presenting self-antigens could mediate bystander tolerance.

The reduction in Cit-Vim–specific T cell numbers with higher doses of DEN-181 suggests deletion, potentially through activation-induced cell death (AICD) with higher doses of antigen. Alternatively, cells may have relocated from PB to other tissues. While the majority of CII- and Cit-Vim–specific T cells in PB were naive or Tcm at baseline, Cit-Vim–specific T cells were enriched in memory and PD-1^+^ cells. Thus, we hypothesize they were more susceptible to AICD. T cells under replication stress in RA, as may be triggered by antigen-specific immunotherapy, may be particularly sensitive to AICD due to mitochondrial defects that reduce their viability (27, 28). Deletion of high-affinity Tem cells could also impact survival of lower affinity tolerant T cells through consumption of homeostatic cytokines (36). Improved disease activity was associated with early expansion of CII-specific and Cit-Vim–specific T cells, suggesting that deletion of autoreactive CD4^+^ T cells with 3 mL DEN-181 was detrimental to disease activity while expansion of autoreactive CD4^+^ T cells supported immunoregulation.

In the transition to RA onset, ACPA IgG VDG increases through N-linked glycan addition after somatic hypermutation, likely due to interaction between Th and autoreactive B cells (30, 31). B cell receptor VDG reduces binding strength of ACPA^+^ B cells for Cit antigens, conceivably enhancing their survival and/or selection (32). The trends in ACPA VDG reduction from 2 to 8 weeks after a 0.3 mL dose of DEN-181 and correlation with reduced DAS28CRP and naive B cells intriguingly suggest DEN-181 may have been able to impact this process, potentially through bystander Cit-Vim–specific Tcm cells interacting in follicles with ACPA^+^ memory B cells. Depletion or arrest of B cells/plasma cells producing the most highly glycosylated ACPA, or modulation of the glycosylation/sialylation machinery of interacting B cells, are possible mechanisms. As the sample size here was small, our data set up interesting hypotheses for future trials.

For ASIT-induced long-term disease control, multiple low-dose or escalating-dose TCR activation steps are likely to be required in patients with recent-onset disease or in the late preclinical phase (37). Using such regimens, trials of peptide ASIT or anti-CD3 immunotherapy in T1D identified exhausted CD8^+^ T cells and IL-10 responses to peptide restimulation (31, 38). The CD8^+^ CTL clonotype skew toward an exhausted transcriptomic signature that we observed after 1 mL DEN-181 resembles the impact of anti-CD3 or LFA3-Ig immunotherapy on CD8^+^ T cells in T1D (16). However, T cells in RA are metabolically disturbed and subject to activation-induced pyroptosis (28). Thus, RA-appropriate, antigen-specific T cell tolerance biomarkers, and appropriate doses of TCR-mediated immunotherapeutics in RA, were not known.

Markers of Tregs are limited in humans, which influences the design of PB biomarker readouts for trials of ASIT. Although we used CD25^+^CD127^–^ to identify Tregs (39), not all cells with this phenotype are regulatory (40, 41), and a single dose of ASIT may be insufficient to stabilize Tr1 differentiation (9, 13). Furthermore, preclinical models suggest the focus of Treg activity is in lymphoid organs (13, 42). Markers correlating with DAS28CRP response after DEN-181 (Figure 4G) and transcripts associated with TCR signaling and regulation identified by scRNA/TCR-Seq represent preliminary biomarkers of tolerance that could be assessed further in appropriate cohorts of ACPA^+^ patients or people at risk of RA. Nur77, encoded by *NR4A1*, is a transcription factor whose expression reports antigen-specific TCR signaling in T cells (43). It is upregulated in tolerant CD4^+^ T cells, where it represses genes mediating effector functions. In mice NR4A family members were necessary to maintain a functional Treg pool (44). *AREG*, an epidermal growth factor receptor ligand, is induced in human T cells by TCR ligation, concomitant with CD69 and with reduced cytokine gene expression (45). Treg amphiregulin promotes tissue repair in injured organs (46). It will be of interest to determine in future clinical studies whether repeated doses of ASIT further expand PD-1^+^Nur77^+^ T cells with regulatory phenotype and functions.

Our immune assays suggest multiple immunoregulatory effects of DEN-181 (13), some of which were associated with disease activity. However, these are exploratory studies and are limited by small sample size and interindividual variability. Due to limited sample availability, we did not undertake assays of self-peptide responses to examine in vitro proliferation or cytokine production. Furthermore, we only assessed 1 antigen-specific bystander population. In future trials, it would be valuable to monitor viral and tetanus antigen-specific T cells to compare the impact of ASIT on T cells recognizing infectious antigens.

Our study has limitations. The primary outcomes of the trial were safety and effects on immune function, and we were limited to assessment of a single ascending dose of DEN-181. Changes in antigen-specific T cell responses among treated patients may have been more marked in a multidose trial. The tetramer flow cytometry sample size was limited by the availability of samples, and ACPA glycosylation analysis was limited to samples with sufficient ACPA. However, the inclusion of a double-blinded placebo control facilitated the interpretation of all data. Comparisons of disease activity are limited by the small numbers of patients per group with variability in disease activity, MTX dose instability, variability in disease duration of participants, and intervention with a single DEN-181 dose.

In this exploratory phase I trial, a single s.c. dose of CII/calcitriol liposomes was safe, and we observed dose-associated immunomodulatory effects in HLA-DRB1*04:01/01:01^+^ ACPA^+^ RA patients on MTX. Future studies should assess the impact of repeat dosing of ASIT in people with ACPA^+^ RA or who are at risk, to test that hypothesis that ASIT expands and differentiates PD-1^+^ autoreactive Tregs that control proinflammatory T cells and autoreactive B cells to suppress disease.

## Methods

### Study patients

Inclusion criteria were a diagnosis of RA (American College of Rheumatology 2010 criteria) treated with MTX at a stable dose for at least 4 weeks prior to planned start of trial treatment, positive anti-CCP, and carriage of HLA-DRB1*04:01 and/or HLA-DRB1*01:01. Exclusion criteria were treatment with more than 10 mg prednisone/d within the last 2 weeks; serious infection or major surgery within the previous 28 days; malignancy or significant cardiovascular, renal, liver, neurological, or skin disease; positive hepatitis or HIV viral serology; pregnancy; or drug abuse. The duration of stable dose of MTX was decreased to 2 weeks after commencement of the trial.

### Study design

Between November 9, 2017, and March 31, 2019, we conducted a randomized, single-center, double-blind, first-in-human, placebo-controlled, prospective phase I trial at the Princess Alexandra Hospital, Brisbane, Australia, in which liposomes encapsulating CII and calcitriol, designated DEN-181, were randomized to placebo (PBS) or 1 mL, 0.3 mL, or 3 mL DEN-181 administered once s.c., in 3 cohorts with sentinel dosing (Table 1 and full protocol in Supplemental Data). The primary objective was safety and tolerability, and the secondary objectives were antigen-specific and bystander T cell immunomodulation, preliminary clinical efficacy, and plasma calcitriol concentrations after DEN-181. The study was submitted under the Australian Therapeutic Goods Administration Clinical Trial Notification (CTN) scheme and approved by the Metro South Human Research Ethics Committee.

Patients with RA were recruited and studied according to the protocol and its statistical analysis plan, except — in a protocol violation — the first cohort received 1 mL rather than 0.1 mL DEN-181 in 1 s.c. site (1 mL intended as the highest dose). Given no impact on safety and the exploratory nature of the trial, the investigators agreed to continue with the 0.3 mL (divided dose) cohort followed by a 3 mL (divided dose) cohort. The study flow is outlined in the flowchart (Supplemental Figure 1). Patients’ safety was monitored and assessed according to the CTC Version 2.0 at continuous time points.

### Preparation of DEN-181

DEN-181 was prepared from egg phosphatidyl choline/egg phosphatidyl glycerol lipids, calcitriol, and CII using an extrusion-based method to yield 89 nm negatively charged liposomes encapsulating 42 μg/mL CII and 0.6 μg/mL calcitriol. DEN-181 was manufactured according to current good manufacturing practices (cGMP) standards by Evonik Canada Inc. The manufactured drug was assayed extensively to assess drug safety, potency, purity, and identity. The active pharmaceutical ingredients used in the drug product (CII peptide and calcitriol) were also produced according to cGMP by Auspep Clinical Peptides (Australia) and Dishman BV (the Netherlands), respectively. All features and components remained within specification at –20°C for at least 20 months.

### Pharmacokinetic analysis of calcitriol

Plasma samples were taken at baseline and 1, 2, and 4 hours after DEN-181 dosing, then immediately frozen. Thawed samples were extracted and analyzed for calcitriol concentration using mass spectroscopy (Eurofins DLS).

### Flow cytometric analysis of PBMCs

Thawed PBMCs were rested for 30 minutes at 37°C, then stained with surface markers to assess leukocyte populations (B cells, T cells, NK cells, DCs, monocytes) (Supplemental Table 2). All antibodies were sourced from BD Biosciences and BioLegend. Data were collected on a BD LSRFortessa X-20 flow cytometer, using a validated assay at Precision for Medicine and analyzed from exported files using Kaluza software (Beckman Coulter). Live cells were identified as viability dye negative, and gates were set based on fluorescence minus one controls using a predetermined gating strategy. For the tetramer assay, samples were thawed, stained with tetramer and surface markers (Supplemental Table 2), acquired, and analyzed as previously detailed (25). All had viability greater than 90% after thaw. The numbers of tetramer^+^ cells were calculated per 10^6^ CD4^+^ T cells, as well as percentage gated of each subpopulation.

### Analysis of serum

Serum samples were collected and frozen within 16 hours of collection, then stored at –80°C. Cytokines were analyzed in duplicate in a single batch using Meso Scale Discovery kits using reference standards supplied by the manufacturer in a validated assay (TetraQ). Cytokines and chemokines were selected as relevant to immune response, inflammation, and tolerance and as observed as potential signals of response to modified DC immunotherapy in RA (12).

### CCP2 ELISA

Serum ACPA levels were determined using anti-CCP2 ELISAs as previously described (47), except that precoated streptavidin plates (Microcoat Biotechnologie GmbH) were used in combination with 1 μg/mL CCP2-biotin or its arginine variant (CargP2-biotin). The lower detection limit was set at 70 AU/mL, based on the value at which 9/10 healthy donor (HD) samples were negative; the upper detection limit (1,600 AU/mL) was set at the highest value of the standard curve.

### ACPA VDG

Determining the percentage VDG on ACPA IgG was essentially performed as described previously (48). In short, ACPA IgG was isolated from patient serum and subsequently dried by vacuum centrifugation (explained in ref. 48). Glycans were released by peptide N-glycosidase F (MilliporeSigma), and released glycans were labeled with 2-aminobenzoic acid and 2-picoline borane. Labeled glycans were purified using hydrophilic interaction liquid chromatography–solid phase extraction. Samples were analyzed using ultra-HPLC, and glycan peaks were aligned and annotated (GP1–GP24) using Chromeleon (version 7.1.2.1713), after which chromatograms were exported. Chromatograms were checked blindly, and samples were excluded in case of aberrant peak distribution and/or lacking the 3 most abundant Fc-glycan peaks (GP4, GP8, and GP14).

Data analysis was performed as described earlier (48). In brief, HappyTools (version 0.1-beta1, built 191209a) (49) was used to calibrate, baseline correct, and integrate the peaks. The cutoff was based on all blank samples by taking the average AUC of all peaks + (12 × SD). The percentage VDG was calculated by dividing the summed AUC of the 3 most abundant VDG peaks (GP19, GP23, and GP24) by the summed AUC of the 3 most abundant Fc-glycan peaks (GP4, GP8, and GP14) × 100 (50).

### ACPA Fc-glycosylation

ACPA IgG Fc-glycosylation was analyzed by mass spectrometry essentially as described (51). In short, ACPA IgG was isolated from patient serum, as well as from ACPA-negative HD serum (*n* = 12) to determine the cutoff (48). Samples were dried by vacuum centrifugation, then digested with trypsin, and the resulting glycopeptides were subsequently analyzed by nano–liquid chromatography-mass spectrometry using an ACQUITY UPLC BEH C18 column (130 Å, 1.7 μm; 75 μm × 100 mm; Waters) for the glycopeptide separation.

Mass spectrometric signals of IgG1 glycopeptides (peptide sequence: EEQYNSTYR) were aligned, calibrated, and integrated using LaCyTools (version 1.1.0-alpha, built 190207b) (52). A glycopeptide was determined to be present when fulfilling the following criteria: 1) mass error between –25 and 25 ppm, 2) deviation from theoretical isotopic pattern < 25%, 3) signal-to-noise ratio > 5. The analysis of all samples was continued only with glycopeptides present in >50% of the ACPA^+^ samples. Lower detection limit was set at the summed signal intensity at which 11/12 HD samples were negative.

The signal intensity of each ACPA-glycopeptide sample above the detection limit was normalized to 100%, after which the percentages of fucosylation, bisection, galactosylation, sialylation, and sialylation divided by galactosylation were calculated.

### Single-cell RNA and TCR sequencing

#### Cell sorting and staining.

Cryopreserved PBMCs were thawed as above and 0.5 × 10^6^ cells stained with Live/Dead FVS700 (BD Biosciences) for 20 minutes at 4°C followed by staining with anti–human CD45 FITC (clone HI30, BD Biosciences). The day 1 PBMC samples were then labeled with 0.4 μg TotalSeq-C0256 anti-human Hashtag 6 (LNH-04; 2M2, BioLegend) and day 29 samples with 0.4 μg TotalSeq-C0257 anti-human Hashtag 7 (LNH-04; 2M2, BioLegend) followed by thorough wash steps. Viable (Live/Dead^–^) CD45^+^ leukocytes from PBMCs were collected by FACS (FACSAria II, BD Biosciences) using a 100 μm nozzle directly into 500 μL of 100% FBS.

#### Generation of single-cell libraries and sequencing.

A total of 10,000 cells from each patient’s day 1 (Hashtag6-labeled) and day 29 (Hashtag7-labeled) PBMCs were combined to give a 20,000 cell total for loading into each well. Single-cell suspensions were partitioned and barcoded using the 10x Genomics Chromium Controller (10x Genomics) and the Single Cell V(D)J Library and Gel Bead Kit (10x Genomics; PN-1000165). The cells were loaded onto the Chromium Single Cell Chip G (10x Genomics; PN-1000120 or PN-1000127) to target 20,000 cells or the maximum cell number. Genetically engineered model generation and barcoding, cDNA amplification, T and B cell enrichment, and cell surface protein and library construction was performed according to the 10x Genomics Chromium User Guide. The resulting single-cell transcriptome libraries and V(D)J-enriched libraries contained unique sample indices for each sample. The libraries were quantified on the Agilent BioAnalyzer 2100 using the High Sensitivity DNA Kit (Agilent, 5067-4626). Libraries were pooled in equimolar ratios. Sequencing was performed using the Illumina NovaSeq 6000. The library pool was diluted and denatured according to the standard NovaSeq protocol (Document 1000000106351) and sequenced using an SP 100 cycle flow cell or an S2 100 cycle flow cell as follows: 26 bp (Read1), 8 bp (i7 index), 91 bp (Read2). Sequencing was performed at Microba based at Translational Research Institute. Data preprocessing was performed using cellranger 4.0.0 (10x Genomics) and a human GRCh38 reference. Final read depth was more than 15,000 reads per cell across all samples. Single-cell library preparation and sequencing and the cellranger computations were carried out at the University of Queensland Sequencing Facility based at the Institute for Molecular Bioscience.

#### Demultiplexing and quality control preprocessing.

Single-cell RNA-sequencing data (deposited in NCBI’s Gene Expression Omnibus, GEO; ref. 53) accessible through GEO Series accession number GSE208161 (https://www.ncbi.nlm.nih.gov/geo/query/acc.cgi?acc=GSE208161) were analyzed with R software using the Seurat package (version 3) following steps described by Stuart et al. (54). The day 1 and day 29 PBMC samples from each patient were deconvoluted using the *HTODemux* function with the positive quantile parameter set to 0.95 for 0.3 mL and 1 mL patient samples, 0.99 for 3 mL, and 0.85 for placebo. Identities were assigned based on *HTO_maxID* and singlets subsequently extracted. Putative artifacts and low-quality cells were filtered out from each sample by retaining only transcriptomes that were within 2 SDs of the mean with respect to both unique gene (feature) counts (nFeature_RNA) and proportion of mitochondrial transcripts (percent.mt). The number of cells per sample passing quality control is provided in Supplemental Table 4.

#### Principal component analysis and UMAP clustering.

All day 1 and day 29 demultiplexed samples were log-normalized (using Seurat’s *Normalise* function) followed by *FindVariableFeatures* to determine the 2,000 most variable features within each data set. Following this, data sets were integrated with the *FindIntegrationAnchors* and *IntegrateData* functions as proposed by the Satija lab and thereby also batch corrected (54). The standard workflow for clustering and visualization was then performed with default parameters (*RunUMAP*; dimension of 30, *FindNeighbors* and *FindClusters* functions, using the Louvain algorithm, with dimension of 30 and a resolution of 0.5). Clusters were annotated based on expression of known marker genes, including *CD3D* (T cells), *NKG7*, *GNLY* (NK cells), *CD79A* (B cells), *CD14*, and *FCGR3A* (monocytes/DCs) (Supplemental Figure 6).

#### Processing of single-cell TCR-sequencing libraries and T cell clustering.

For TCR analysis, only cells with full-length, productive, and paired TCRα/β were considered. For subsequent CD3^+^TCR^+^ analysis, the Seurat object containing all transcriptomes was subclustered on cells showing *CD3D* expression (clusters 1, 2, 5, 6, 8, 9, 10, 12, 14, 15, 16) using default parameters. The TCR information was integrated with the CD3^+^ transcriptomic analysis, and only cells that were both CD3^+^ and TCR^+^ were reclustered (Supplemental Figure 6B). CD3^+^TCR^+^ cell clustering was performed as above with a resolution of 1.6 in the *FindClusters* function.

#### CD3^+^TCR^+^ cluster annotation.

T cell clusters were annotated at a first level based on interpretation of T cell–defining markers, using heatmap and FeaturePlots (Supplemental Figure 6). Clusters were also confirmed by identifying differentially expressed marker genes for each cluster and comparing to known T cell type-specific marker genes (Supplemental Figure 7). We also downloaded bulk RNA-Seq count data from immune cell populations identified in previously published studies — Tnaive, Tcm, and Tem (GEO GSE11057) cells; CD8^+^ exhausted T (Tex) and CD4^+^ Tex cells (GEO GSE41870); CD4^+^ Tregs (55); and CD4^+^ CTLs and CD4^+^ Tfh cells (56) — and compared these to the expression profiles from our single-cell clusters using AUCell (57) and GSEABase as previously described (58) (Supplemental Figure 7). Monocle 2 was used to order cells in pseudotime and to generate the cell fate trajectory to further support T cell annotation (59). Finally, we assigned scores for S and G2/M cell cycle phases based on previously defined gene sets (60) using the CellCycleScoring function in Seurat.

### Statistics

The patients were followed longitudinally according to the flowchart (Supplemental Figure 1). One subject in the 3 mL cohort missed visit 4. Not all samples were available for tetramer or single-cell RNA sequencing analysis for each participant and each time point, due to sample depletion. No sera were collected from the 1 mL dose cohort. Analysis of safety, efficacy, and immunological parameters was descriptive, including interparticipant comparisons. Interparticipant and intraparticipant comparisons over time used longitudinal methods, including linear mixed models. Nonclinical variables collected longitudinally were expressed as change (Δ) with respect to the nontreated baseline (day 1). We determined the maximum increase in percentage PD-1^+^ T cell populations (E_max_), as previously described (that is, E_max_ = peak value/baseline value × 100%). Groups were compared over time using a mixed effects model with the Geisser-Greenhouse correction to determine effect of group, time, or group × time interaction. Variance is displayed as median ± interquartile range with all data points plotted. A *P* value less than 0.05 was considered significant.

We conducted principal component analysis for the cell count data (19 variables, 47 samples with individuals measured on days 1, 8, and 29), serum factor concentration data (14 variables, 60 samples with individuals measured on days 1, 29, and 57), and phenotype data (41 variables, 33 samples with individuals measured on days 1, 8, and 29) to identify whether the major sources of variation in the data could be explained by treatment and day (mixOmics R package v 6.16.3) (61). The loading coefficient of each variable indicates its contribution to define each principal component. We then calculated the Spearman’s correlation of each of these variables with the DAS28CRP across their 3 time points. Clustering of the correlation trajectories was performed with K-medoids (partitioning around medoids, ref. 62; fpc R package v2.2-9), using the silhouette coefficient to choose the optimal number of clusters (cell data = 5, serum data = 5, phenotype data = 8). These analyses used the statistical software R 4.1.0.

### Study approval

The study was approved by the human ethics committees of the Metro South Health Service, Brisbane, Queensland, Australia, and University of Queensland, Brisbane, Australia, under the CTN scheme, and written informed consent was obtained from all patients prior to participation. The trial is registered: anzctr.org.au identifier: ACTRN12617001482358, last updated September 8, 2022.

Prior publication: This work was presented at the American College of Rheumatology and the Association for Rheumatology Professionals Annual Meeting in Atlanta, Georgia, USA, on November 12, 2019.

## Author contributions

RT had full access to all of the data in the study and takes full responsibility for the integrity of the data and the accuracy of the data analysis. RT, H Reid, KALC, IG, MR, and KC contributed to the study concept and design. AS, HJN, PW, NR, SP, KAVS, MWB, AM, JT, MT, JR, H Reid, FES, H Roberts, PV, IG, MR, SB, REMT, HUS, KALC, KC, and RT contributed to the acquisition, analysis, or interpretation of data. RT drafted the manuscript. AS, HJN, PW, NR, SP, KAVS, MWB, AM, JT, MT, JR, H Reid, FES, H Roberts, PV, IG, MR, SB, REMT, HUS, KALC, KC, and RT contributed to the critical revision of the manuscript for important intellectual content. RT and REMT obtained funding. HJN, PW, NR, SP, KAVS, MWB, AM, MT, H Reid, JR, SB, KALC, KC, REMT, and HUS provided technical and statistical support. AS, SP, JT, H Roberts, PV, MR, SB, and KC provided clinical and project support. For equal first authors, order was defined as clinical followed by technical contributors, with the latter in alphabetical order.

## Supplementary Material

Supplemental data

Supplemental data 2

ICMJE disclosure forms

Supplemental table 5

## Figures and Tables

**Table 1:**
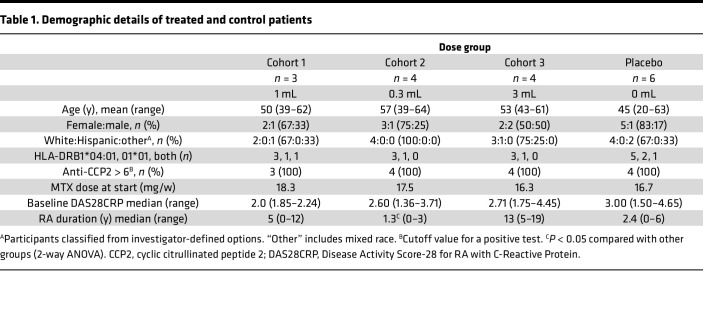
Demographic details of treated and control patients

**Table 2:**
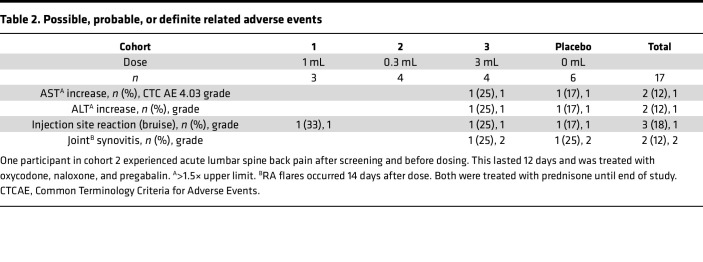
Possible, probable, or definite related adverse events

## Version 1 10/24/2022

Electronic publication
