# Supplementary material for: Randomized phase I trial of antigen-specific tolerizing immunotherapy with peptide/calcitriol liposomes in ACPA+ rheumatoid arthritis
Source: JCI Insight. 2022 Oct 24;7(20):e160964. doi: 10.1172/jci.insight.160964 (PMC9714780; doi:10.1172/jci.insight.160964)
Supplement: Supplemental data [file jciinsight-7-160964-s094.pdf]

Randomized phase-1 trial of antigen-specific tolerizing immunotherapy with peptide/calcitriol liposomes in ACPA+ rheumatoid arthritis

Supplementary materials

Supplemental Table 1. Individual participant medication data and possible infections for the 17 study subjects

Supplemental Table 2. Flow cytometry panels used in analysis of PBMC

Supplemental Table 3. Serum analytes measured in this study

Supplemental Table 4. Quality control threshold values and cell numbers for downstream scTCR/RNA-seq analysis

Supplemental Table 5. DEGs of CD3+TCR+ T cells (FindAllMarkers), with tabs showing each cluster vs all other cells (FindMarkers)

Supplemental Figure 1. Flow chart of the study

Supplemental Figure 2. Representative flow cytometry plots of tetramer assay.

Supplemental Figure 3. Individual trajectories of the number of CII-specific, Cit-Vim-specific and CD4+ T cells; changes in CD4+ and CD8+ T cell sub-populations at day 8 relative to day 1; individual trajectories of DAS28CRP.

Supplemental Figure 4. Changes in ACPA fucosylation, bisection, galactosylation, sialylation and sialylation/galactosylation, cytokine and chemokine concentration and PB non-T cell numbers.

Supplemental Figure 5. DAS28 of participants whose samples were analyzed by single cell transcriptomics

Supplemental Figure 6. Characterisation of the global immune cell landscape

Supplemental Figure 7. Transcriptomic identification of CD4 clusters of naïve, central memory, helper, regulatory and cytotoxic T cells and predominantly clonal activated CD8 CTL

Supplemental Figure 8. Transcriptomic analysis of expanded pre-existing T cell clonotypes before and after treatment

Full protocol

Supplemental Table 1. Individual participant medication data and possible infections

Timing of medication changes and infections is indicated relative to test article dosing.

Coh-ort	#	Duration RA (yr)	wt (kg)	MTX dose (mg)	MTX change before trial	Prednisone	Medication changes	Possible Infections
1	004	0.2	81.8	20	-11mth 20 -> 10mg			
1	005	12.1	116.0	10		prn		
1	010	3.1	55.8	25				
2	011	0.7	78.0	25				+6w common cold
2	013	1.3	79.0	20	-1mth 10-> 20mg			
2	015	3.3	55.9	5				+1mth URTI
2	016	1.7	80.5	20		-5mth 5 mg/d	-12d oxycodone reduction schedule	
3	020		72.2	25			+2w clindamycin started	+2w skin infection
3	021	17.7	49.7	10				
3	022	5.7	73.2	20	-1mth 10-> 20mg		+3d codral flu stopped	cold/flu at dosing
3	025	19.9	64.8	10		+2w 7.5 mg/d	+1d amoxicillin started	LRT at dosing
P	001	1.9	77.8	10				
P	003		72.3	10			-1mth Tacrolimus stopped	
P	012	5.2	74.2	25		+2w 7.5 mg/d	+1w Meloxicam started	+2w flu-like symptoms
P	014	6.4	99.8	20	-1.5mth 10-> 20mg		-1.5mth SSZ, HCQ stopped	
P	019	0.7	105.0	25	-1mth 20-> 25mg sc		-1mth HCQ stopped, -2w meloxicam increased	
P	026	0.5	54.0	10				

Supplemental Table 2. Flow cytometry panels used in analysis of PBMC

T cell and tetramer panel

Marker	Clone	Fluorochrome	Source
DRB1*04:01/DRB1*01:01-Collagen $II_{259-273}$ or DRB1*04:01/DRB1*01:01-Cit64-$Vimentin_{59-71}$	Tetramers	PE	Department of Biochemistry and Molecular Biology, Monash University
CD19	HIB19	FITC	Biolegend
CD14	HC14	FITC	Biolegend
CD16	3.9	FITC	Biolegend
CD11c	3G8	FITC	Biolegend
CD3	UCHT1	BUV737	BD Biosciences
CD4	SK3	BUV395	BD Biosciences
CD25	BC96	BV650	Biolegend
CD127	A019D5	BV421	Biolegend
CCR7	2-L1-A	BV510	BD Biosciences
PD1	EH12.1	BB700	BD Biosciences
CD45RO	UCHL1	APC-H7	BD Biosciences

Non-T cell panel

Marker	Clone	Fluorochrome	Source
CD24	ML5	BUV395	BD Biosciences
CD3	UCHT1	BUV737	BD Biosciences
CD19	HB19	BV421	Biolegend
HLA-DR	G46-6	BV480	BD Biosciences
VIABILITY	FV5 575V	BV605	BD Biosciences
CD1c	F10/21A3	BV650	BD Biosciences
CD27	M-T271	BV711	Biolegend
CD14	M5E2	BV786	Biolegend
CD8	RPA-T8	FITC	Biolegend
CD141	1A4	BB700	BD Biosciences
CD123	9F5	PE	BD Biosciences
CD56	NCAM16.2	PE-CF594	BD Biosciences
IgD	IA6-2	PE-Cy7	Biolegend
CD38	HIT2	APC	Biolegend
CD16	3G8	APC-H7	BD Biosciences

Supplemental Table 3. Serum analytes measured with Mesoscale

CX3CL1 (Fractalkine)

IL-10

IL-12p40

IL-15

IL-27

IL-2Ra (sCD25)

IL-6

IL-7

CXCL11 (ITAC)

Serum amyloid A (SAA)

Tumor necrosis factor (TNF)

Vascular endothelial cell growth factor alpha (VEGFA)

Supplemental Table 4. Quality control threshold values and cell numbers for downstream scTCR/RNA-seq analysis.

Sample	Genes (n_Feature)				Percent.mt			# Cells	
	Mean	SD	Min	Max	Mean	SD	Max	Total	Pass QC
Placebo_day1	766.97	241.61	283.75	1250.19	2.32	2.25	6.82	3686	**3429**
Placebo_day29	1039.46	317.31	404.85	1674.08	1.34	1.04	3.42	5544	**5120**
0.3mL_day1	626.01	167.88	290.24	961.77	1.20	0.78	2.75	7027	**6384**
0.3mL_day29	630.93	163.59	303.74	958.11	1.23	0.81	2.85	5747	**5224**
1mL_day1	714.66	195.16	324.35	1104.98	1.41	0.88	3.17	5025	**4568**
1mL_day29	712.30	205.56	301.18	1123.42	1.64	1.03	3.70	6328	**5821**
3mL_day1	860.40	242.26	375.88	1344.92	1.78	0.97	3.72	8649	**7871**
3mL_day29	859.26	237.47	384.32	1334.19	1.75	1.11	3.97	8046	**7414**

Supplemental Table 5. List of key defining differentially expressed genes in CD3+TCR+ dataset.

See Excel spreadsheet (other supporting files)

Supplemental Fig. 1. Flow chart of the study

After pre-screening 56 ACPA+ RA patients on methotrexate (MTX) for HLA-DR, 26 carrying HLA-DRB1*04:01 or *01:01 were screened for trial eligibility. Seventeen were included in the study and assigned to cohorts 1 (1ml), 2 (0.3 ml), or 3 (3ml). Screen failures included patients whose MTX was not stable for 4 weeks, whose ACPA titre was below the cut-off and who decided not to participate for other reasons.

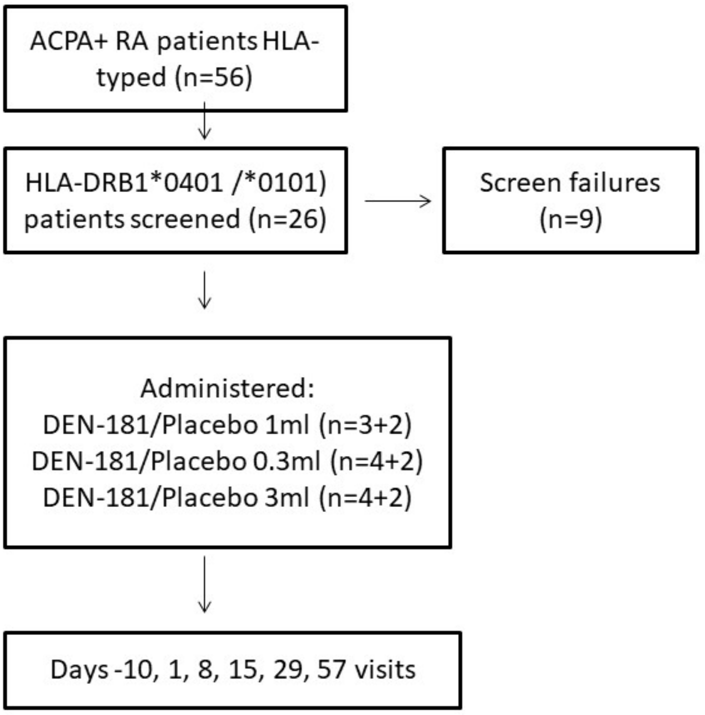

Supplemental Figure 2. Representative flow cytometry plots of tetramer assay.

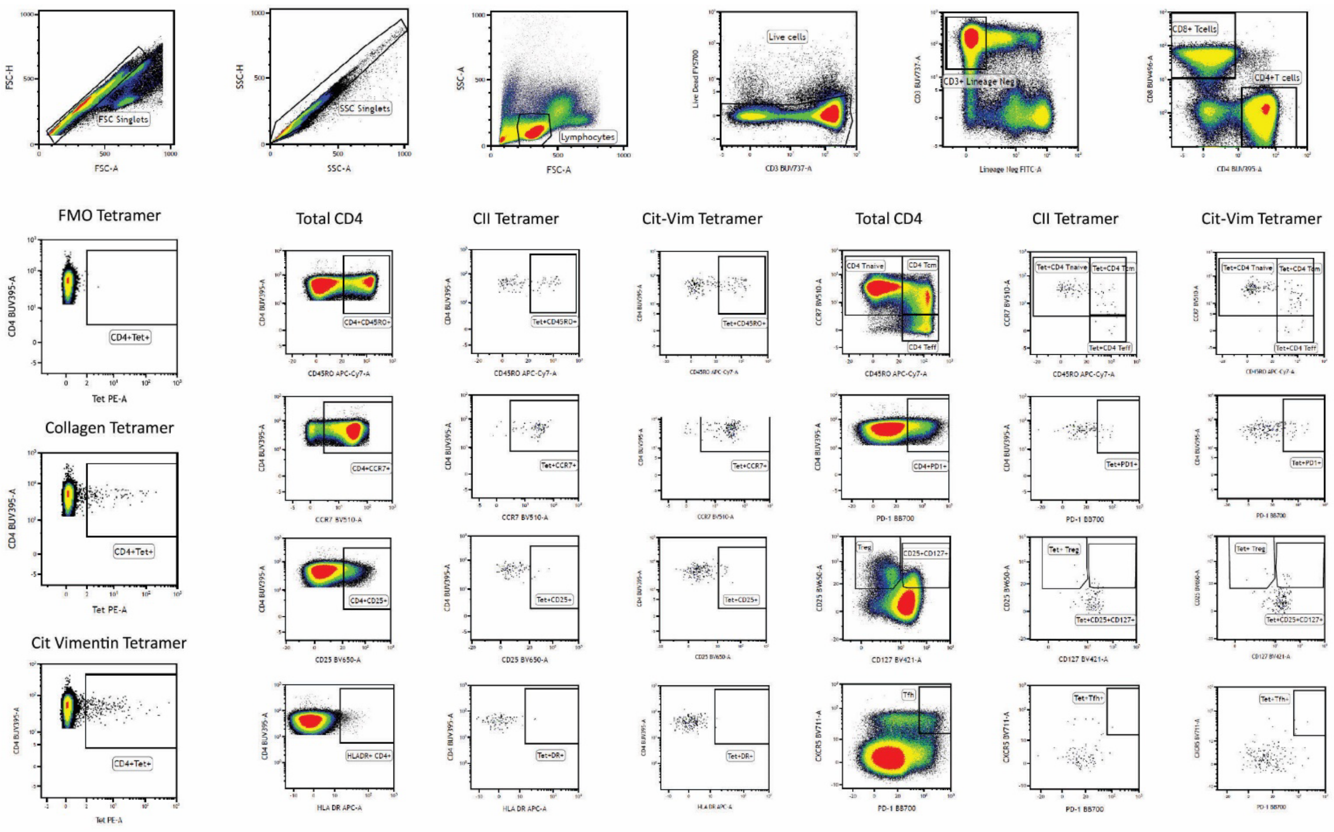

Supplemental Figure 3A. Individual trajectories of the number of CII-specific, Cit-Vim-specific and CD4^{+} T cells

Number of CII-specific and Cit-Vim-specific T cells per 10^{6} CD4^{+} T cells, and CD4^{+} T cells per 10^{6} lymphocytes from day 1 to 29, plotted for each individual across dose groups. CII-specific and Cit-Vim-specific T cells identified by flow cytometry using HLA-DRB1*04:01 and *01:01-$CII_{259\text{-}273}$ and HLA-DRB1*04:01 and *01:01-64Cit-$Vimentin_{59\text{-}71}$ tetramers. DEN-181 dose and tetramer indicated at the top of each graph. Green symbols: HLA-DRB1*01:01 tetramer used.

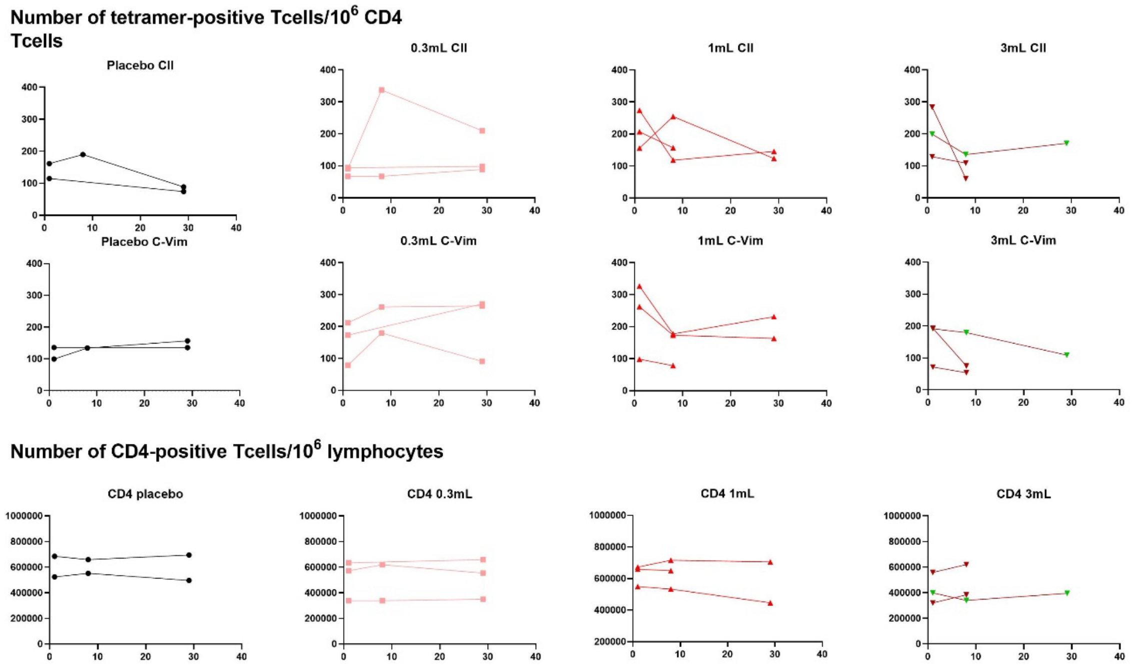

Supplemental Figure 3B. Changes in CD4^{+} and CD8^{+} T cell sub-populations at day 8 relative to day 1

Change in CD4^{+} and CD8^{+} T cell pre-specified phenotypic subset proportion relative to day 1, represented as a heatmap for individuals across dose groups at day 8. Scale +20 to -20%. DEN-181 dose indicated at the bottom of each heatmap.

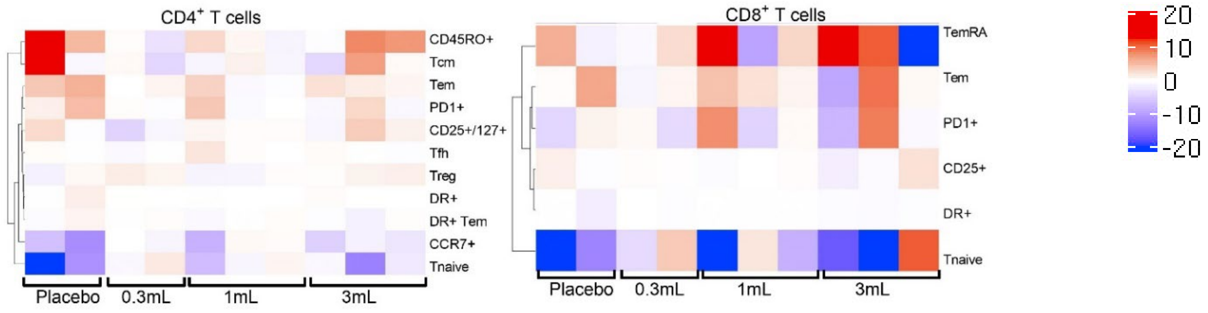

Supplemental Figure 3C. Individual trajectories of DAS28CRP.

DAS28CRP plotted for each individual across dose groups. Light coloured symbols indicate participants who received steroids for flare. DEN-181 dose indicated at the top of each graph. Green symbols denote individuals carrying HLA-DRB1*01:01 and not HLA-DRB1*04:01.

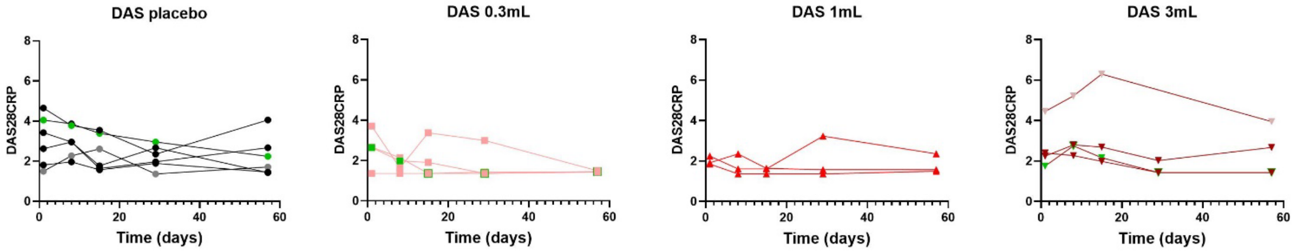

Supplemental Figure 4. Changes in ACPA fucosylation, bisection, galactosylation, sialylation and sialylation/galactosylation, cytokine and chemokine concentration and PB non-T cell numbers.

A. The change in %fucosylation, bisection, galactosylation, sialylation of ACPA Fc, measured by HPLC; B. The change in serum concentrations of fractalkine, IL-12/23p40, IL-7, IL-15, IL-27, IL-2Ra, IL-6, IL-10, ITAC, serum amyloid A, TNF and VEGF-A, measured by electrochemiluminescence; and C. the numbers of B cells, NK cells, monocytes and DCs per 10^6 $CD45^+$ cells, analyzed by flow cytometry. All plotted at days 8, 15, 29 and 56 relative to day 1 for individuals across placebo, 0.3mL, 1mL (cells only) and 3mL DEN-181 dose groups. Each box represents the range (min-max), including all data points.

A.

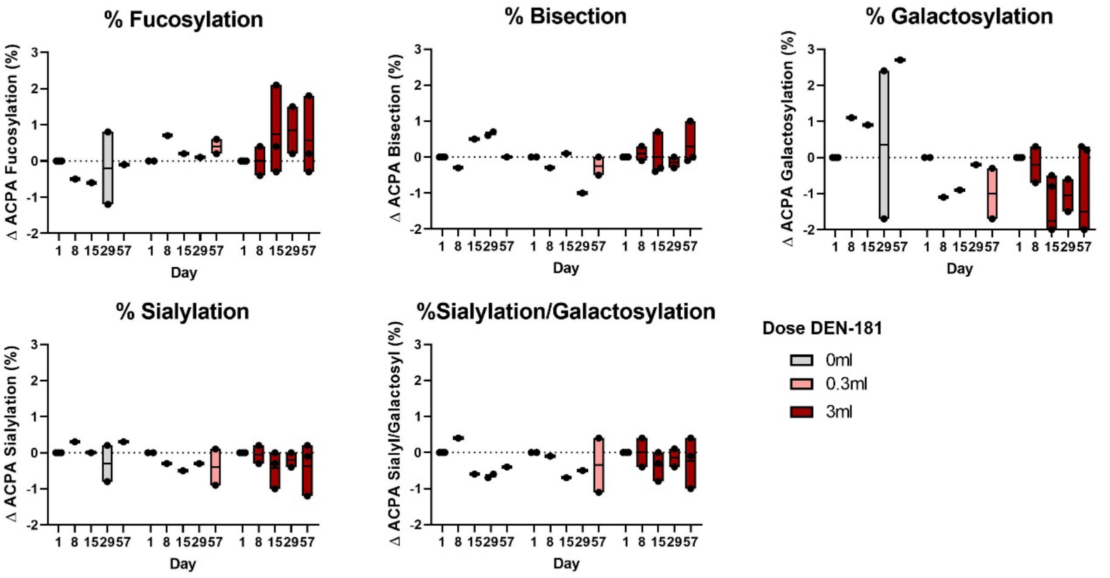

B.

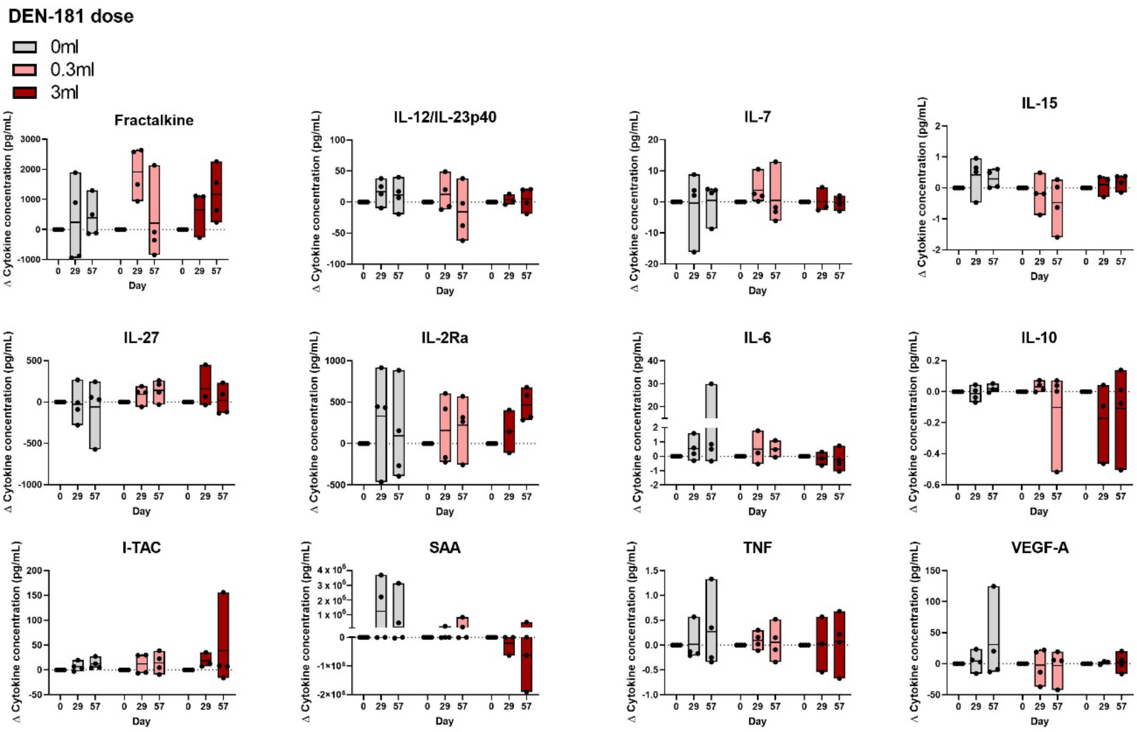

C.

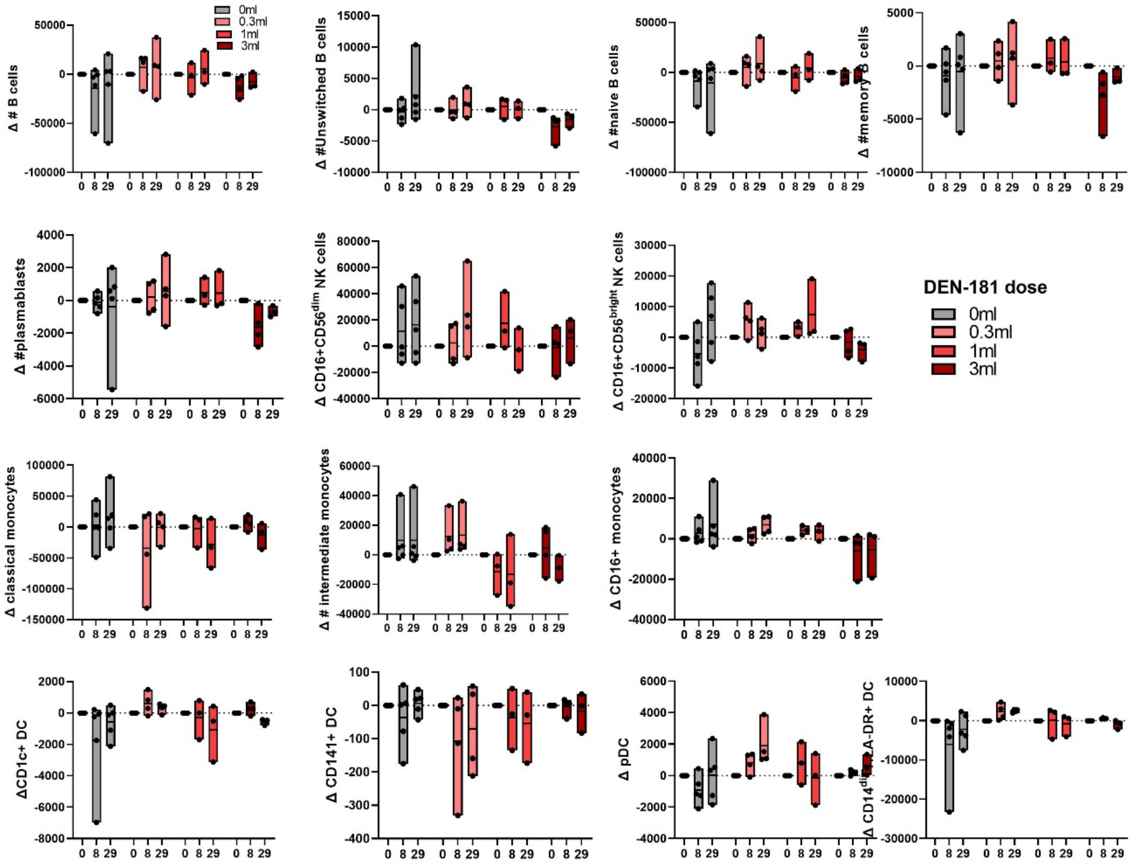
0ml
0.3ml
1ml
3ml
Δ # B cells
Δ #Unswitched B cells
Δ #naïve B cells
Δ #memory B cells
Δ #plasmablasts
Δ CD16+CD56^{dim} NK cells
Δ CD16+CD56^{bright} NK cells
DEN-181 dose
Δ classical monocytes
Δ # intermediate monocytes
Δ CD16+ monocytes
ΔCD1c+ DC
Δ CD141+ DC
Δ pDC
0 8 29

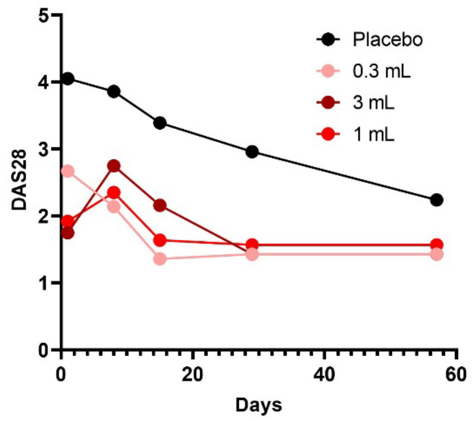

Supplemental Figure 5. DAS28 of participants whose samples were analyzed by single cell transcriptomics

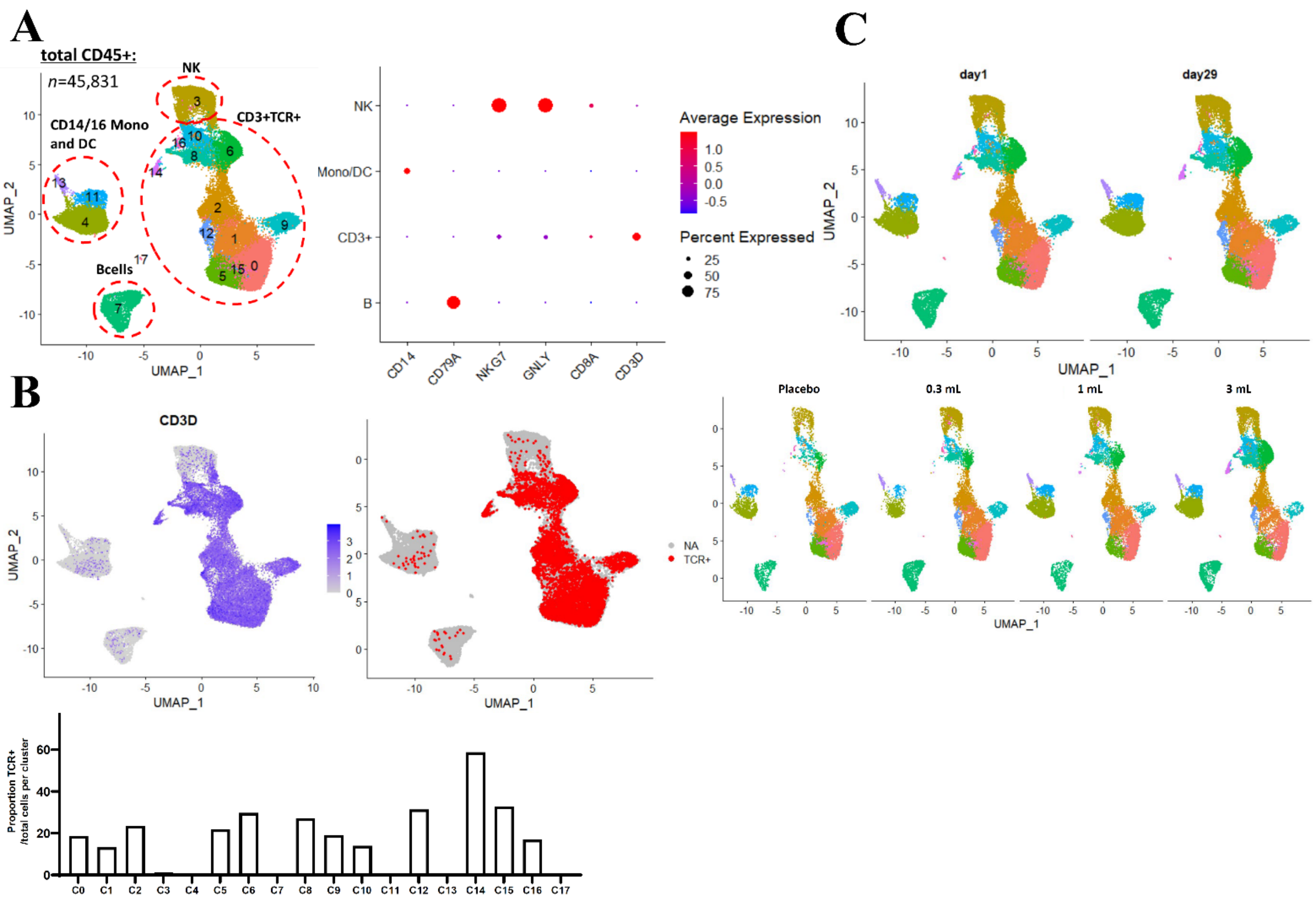

Supplemental Figure 6. Characterization of the global immune cell landscape. A. Uniform manifold approximation and projection (UMAP) of all $CD45^+$ single cells before and after DEN181 treatment in a pooled analysis. Superclusters are annotated based on canonical marker expression depicted in the Dotplot (right). Mono/DC, CD14/16 monocytes and dendritic cells (*CD14*); NK, natural killer cells (*NKG7, GNLY)*; B cells (*CD79A*); and $CD3^+TCR^+$, T cells (*CD3D*). **B.** UMAP depicting *CD3D* expression (left) and cells expressing a productive, paired T cell receptor (TCR)α/β (right). Proportion of TCR^+ cells contained within each cluster are depicted in the bar chart. **C.** UMAP plots split by timepoint (top) and patient (bottom).

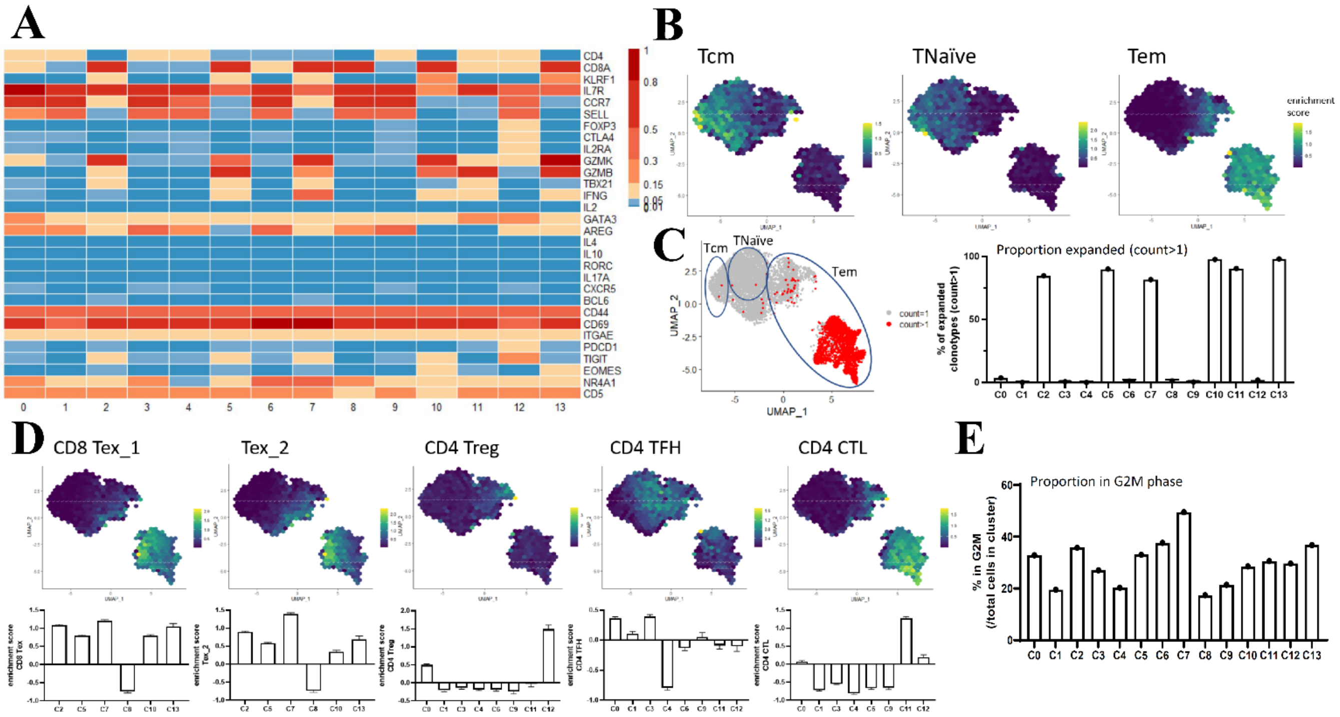

Supplemental Figure 7. Transcriptomics identifies CD4 clusters of naïve, central memory, helper, regulatory and cytotoxic T cells and predominantly clonal activated CD8 CTL. A. Heatmap showing select T cell-defining markers (average gene expression) among the CD3+TCR+ clusters. **B.** UMAP plots displaying the enrichment scores of gene signatures for T central memory (Tcm), T naïve (Tnaïve), and T effector memory (Tem) cells. **C.** UMAP depicting all expanded T cell clonotypes (CDR3 count >1, in red) within the total $CD3^{+}TCR^{+}$ dataset. Bar graph as quantification of UMAP, showing proportion expanded clonotypes per cluster. **D.** Enrichment scores of two exhaustion, CD4 regulatory, CD4 follicular helper and CD4 CTL signatures overlaid onto UMAP. Enrichment scores corresponding to each signature depicted in bar chart below. See methods for more information under *Gene Set Enrichment Analysis* section. **E.** The CellCycleScoring package in Seurat was used to assign cells as either "G2/M" or "S" phase. Proportion of cells within each cluster in G2M phase are shown in the bar chart.

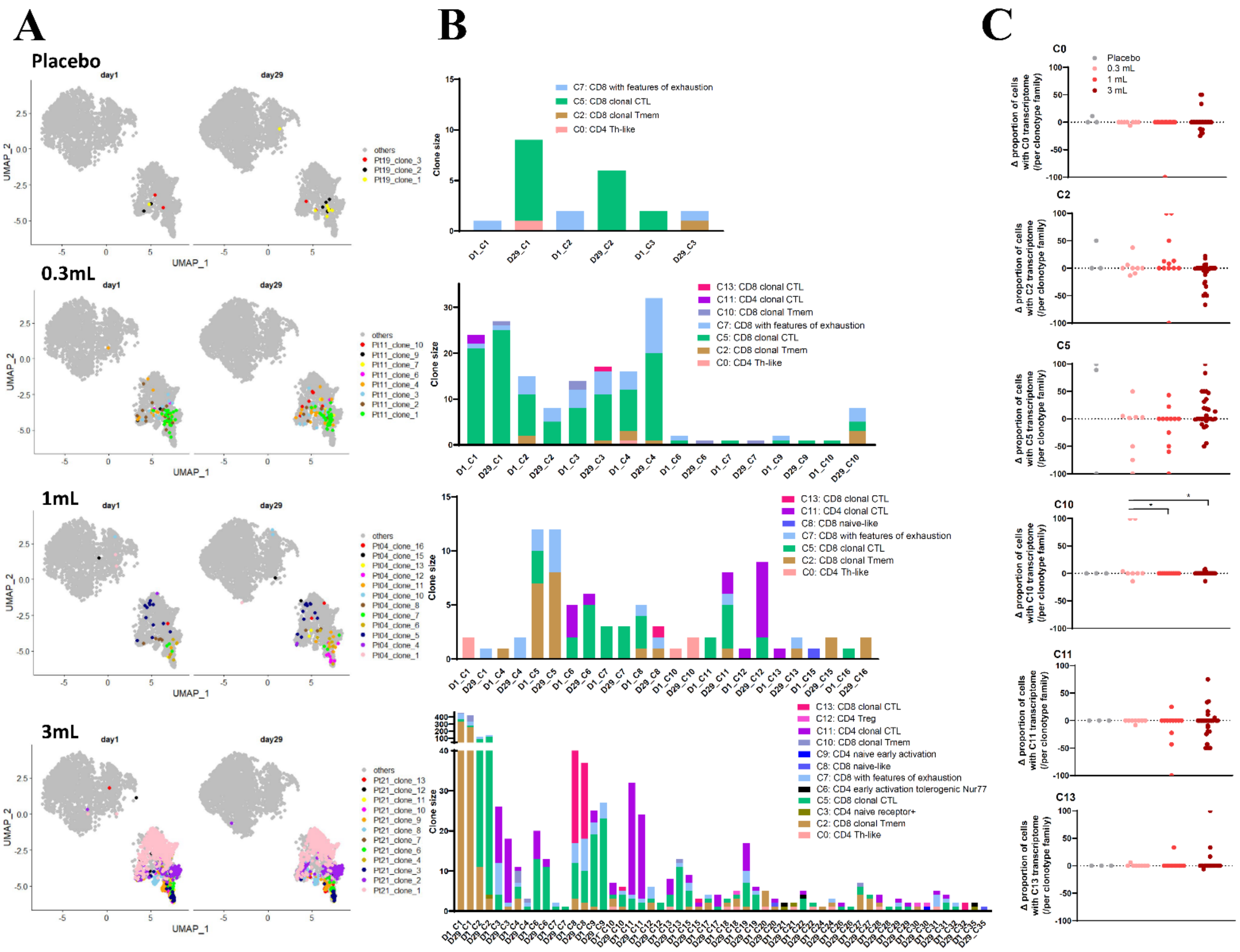

Supplemental Figure 8. Transcriptomic analysis of expanded pre-existing T cell clonotypes before and after treatment. A. UMAP overlay of pre-existing expanded T cell clonotypes (pre-existing defined as being identified at both day 1 and day 29). For 3 mL dose, only top 12 clonotypes are shown on UMAP. **B.** Transcriptomes of single cells within each pre-existing TCR clone family. **C.** The change in T cell clonotype transcriptome within the specified clusters at day 29 relative to day 1 for the placebo, 0.3 mL, 1 mL and 3 mL patients. Ordinary one-way ANOVA with Holm-Sidak's multiple comparisons test were used to compare treatment groups. * p<0.05 for comparison with placebo group as shown.
