# Supplementary material for: Randomized phase I trial of antigen-specific tolerizing immunotherapy with peptide/calcitriol liposomes in ACPA+ rheumatoid arthritis
Source: JCI Insight. 2022 Oct 24;7(20):e160964. doi: 10.1172/jci.insight.160964 (PMC9714780; doi:10.1172/jci.insight.160964)
Supplement: Supplemental data 2 [file jciinsight-7-160964-s095.pdf]

Clinical Study Protocol

**Study Title:**	A Phase I, randomised, double-blind, placebo-controlled, single centre, single-dose escalation and multiple-dose parallel group study to investigate the safety, tolerability, and pharmacodynamics of subcutaneously administered DEN-181 in adult patients with ACPA+ rheumatoid arthritis on stable treatment with methotrexate.
**Sponsor:**	Dendright Pty. Ltd.<br>Registered Office: Level 7, General Purpose South Building<br>Staff House Road<br>The University of Queensland (St Lucia Campus)<br>Brisbane, Queensland, 4072, AUSTRALIA
**Investigational Product:**	DEN-181
**Protocol Number:**	DEN-17-01-RA
**Clinical Phase:**	Phase 1
**Principal Investigator:**	Dr. Phillip Vecchio
**Protocol Version/Date:**	Version 3.0 13 Feb 18

CONFIDENTIALITY STATEMENT

This protocol is the confidential property of Dendright Pty Ltd and is intended solely for the guidance of this clinical investigation. This protocol may not be disclosed to parties not associated with this clinical investigation not used for any other purpose without the prior written consent of Dendright Pty Ltd.

A PHASE I, RANDOMISED, DOUBLE-BLIND, PLACEBO-CONTROLLED, SINGLE-CENTRE, SINGLE-DOSE ESCALATION AND MULTIPLE-DOSE, DOSE-RANGING PARALLEL GROUP STUDY TO INVESTIGATE THE SAFETY, TOLERABILITY, AND PHARMACODYNAMICS OF SUBCUTANEOUSLY ADMINISTERED DEN-181 IN ADULT PATIENTS WITH ACPA+ RHEUMATOID ARTHRITIS ON STABLE TREATMENT WITH METHOTREXATE.

PROTOCOL NUMBER: ***DEN-17-01-RA***

Version 3.0 and Dated 13 February, 2018

This protocol has been approved by the Sponsor. The following signature documents this approval.

HELEN ROBERTS		
Name (Printed)		Signature
Date<br>(dd mmm yyyy)		

INVESTIGATOR STATEMENT

I have read the protocol, including all appendices, and I agree that it contains all necessary details to conduct this study as described. I will conduct this study as outlined herein and will make all reasonable efforts to complete the study within the time designated.

I will provide all study personnel under my supervision copies of the protocol and access to all information provided by the Sponsor. I will discuss this material with them to ensure that they are fully informed about the investigational medicinal products and the study.

PHILLIP VECCHIO		
Principal Investigator's Name (Printed)		Signature
TRI-01		
Site number		Date (dd mmm yyyy)

STUDY SYNOPSIS

Protocol No.:	DEN-17-01-RA
Study Title:	A Phase I, randomised, double-blind, placebo-controlled, single-centre, single-dose escalation and multiple-dose, dose-ranging parallel group study to investigate the safety, tolerability, and pharmacodynamics of subcutaneously administered DEN-181 in adult patients with ACPA+ rheumatoid arthritis on stable treatment with methotrexate (MTX).
Investigational Product:	DEN-181 (liposomal formulation of calcitriol and Pro273Hyp collagen$_{259-273}$ II peptide – GIAGFKGEQGPKGE-Hyp)
Indication:	Rheumatoid Arthritis (RA)
Development Phase:	Phase 1
Rationale:	Current treatments to control pathological immune responses during autoimmunity use broad immunosuppressive drugs associated with undesirable side effects. Alternative strategies to induce antigen-specific immunological tolerance to control both cellular and humoral immune responses are desirable. One such strategy is to use the natural process of antigen presentation by dendritic cells (DCs) to control the balance of pathogenic effector T and B cells versus regulatory T cells (Treg). The investigational product DEN-181 is designed to deliver RA-specific auto-antigenic peptide to lymph node DCs in the presence of the NF-kB inhibitor, calcitriol, with the goal of inducing antigen (Ag)-specific immune regulation.
Investigator(s):	Dr Phillip Vecchio<br>Dr Amee Sonigra
Primary objectives:	To determine the safety and tolerability of single and multiple doses of DEN-181 in patients with ACPA+ RA.
Secondary objectives:	• To determine the ability of DEN-181 to modulate the total T-naïve, T-effector and T-regulatory cell populations in peripheral blood;<br>• To determine the antigen-specific immune response to DEN-181 including the determination of collagen II peptide specific T-naïve, T-effector and T-regulatory cell populations;<br>• To determine preliminary clinical efficacy of DEN-181 in MTX-treated early ACPA+RA patients;<br>• To monitor plasma concentrations of calcitriol
Exploratory objectives:	To investigate the effects of administration of DEN-181 on immune responses, disease biomarkers and the microbiome using various exploratory assays in blood, urine and the gastrointestinal tract.
Primary endpoints:	Clinical safety observations including changes in vital signs, adverse events, serious adverse events, laboratory abnormalities and withdrawals from study. Adverse event severity will be graded according to Common Terminology Criteria for Adverse Events (CTCAE, Version 4.03). Any exacerbation of RA will be evaluated by the DAS28CRPv4 (Disease Activity Score 28 including CRP assessment 4 variable calculation) scoring system.
Secondary endpoints:	• Proportion, total number and phenotype of naïve, effector and regulatory T-cell populations (i.e. total, collagen II peptide-specific, haemagglutinin (HA) peptide-specific) measured by flow cytometry and major histocompatibility complex (MHC) II tetramer analysis pre- and post-administration of DEN-181;<br>• Mean changes in DAS28CRPv4 from baseline in treatment and control groups;<br>• Determination of the concentration of calcitriol in plasma.

Exploratory endpoints:	• Various exploratory immune assays. These measures of humoral and T-cell responses, may include, but are not limited to, the following:<br>o Proportion, number and phenotype of naïve, effector and regulatory T cell populations of other RA autoantigens (e.g., citrullinated vimentin peptide-specific assessments);<br>o Peripheral blood interferon-gamma (IFN-γ) enzyme-linked immunospot (ELIspot) assays to common infectious antigens (cytomegalovirus, Epstein-Barr virus, influenza virus);<br>o Proportions and numbers of myeloid (monocyte and DC), B cell, Natural Killer (NK) cell and T follicular helper (Tfh) cell peripheral blood populations;<br>o Antibody titres of RA autoantigens including collagen, modified collagen and ACPA;<br>o Ex-vivo re-stimulation assays (e.g., as measured by cytokine secretion and tetramer analysis);<br>o T-cell receptor sequencing of specific single cell populations.<br><br>• Various exploratory biomarker readouts which may include but are not limited to the following: cytokine levels, tryptophan/kynurenine levels (HPLC), metabolomics, measures of the cellular transcriptome and the gastrointestinal microbiome.
Study design:	Randomised, double-blind, placebo-controlled, single centre, single-dose escalation and multiple dose, dose-ranging parallel group study to be performed in 2 parts.<br>• Part A: Randomised double blind, placebo-controlled, single centre, single-dose escalation study;<br>• Part B: Randomised double blind, placebo-controlled, single centre, multiple dose, dose-ranging parallel group study at two dose levels.
Number of participants:	<u>Part A</u>:<br>Total=18<br>Dose Level 1 (4 active, 2 placebo)<br>Dose Level 2 (4 active, 2 placebo)<br>Dose Level 3 (4 active, 2 placebo)<br><u>Part B</u><br>Total=40<br>Dose Group A (15 active, 5 placebo)<br>Dose Group B (15 active, 5 placebo)
Number of centres:	Single centre
Inclusion criteria:	1. Diagnosis of RA made by a rheumatologist;<br>2. HLA-DRB1*0401+, HLA-DRB1*0101+ or HLA-DRB1*0401+HLA-DRB1*0101+ heterozygotes, homozygotes or compound heterozygotes and ACPA+;<br>3. Treatment with MTX at the same dose for at least 2 weeks prior to planned start of trial treatment;<br>4. Any swollen joint count at screening (0-28);<br>5. Age 18-75 years, inclusive;<br>6. Male or female. Females of child-bearing potential must agree to use two effective forms of contraception from enrolment to completion of the study;<br>7. Patients must be informed of the investigational nature of this study and give written informed consent in accordance with the institutional and hospital guidelines;<br>8. Blood glucose, CBC, haemoglobin, platelets, creatinine, bilirubin, and AST/ALT not greater than 1.5 x out of normal laboratory ranges at entry and clinically insignificant in opinion of investigator;<br>9. Patients agree to forego vaccinations during the course of the study.

Exclusion criteria:	1. Malignancy;<br>2. An active inflammatory disease other than RA;<br>3. Currently receiving or have received treatment with >10mg prednisone daily within the last 2 weeks prior to screening;<br>4. Current or recent treatment (< 2 weeks prior) with any disease-modifying anti-rheumatic drugs (DMARDS) other than MTX;<br>5. Serious infection requiring hospitalisation within last 28 days;<br>6. Receipt of any live attenuated vaccines within 4 weeks prior to entry;<br>7. Major surgery within last 28 days;<br>8. Significant cardiovascular, renal, liver, neurological or skin disease;<br>9. Positive serology for HIV, or infection with HBV or HCV;<br>10. Current treatment with cytotoxic or immunomodulatory therapies such as radiotherapy, cyclophosphamide, mycophenolate, tacrolimus, PUVA, acitretin cyclosporine or azathioprine;<br>11. Any known or suspected allergies to the study drug or its constituents including egg products;<br>12. Inadequate venous access to allow collection of blood samples;<br>13. History of drug or alcohol abuse;<br>14. Participation in any other clinical trial;<br>15. If, in the opinion of the PI, the subject appears not to be able to perform the needed responsibilities of participation in the clinical study.
Part B Specific Inclusion Criteria	Diagnosis of RA made by a rheumatologist within the previous 2 years.
Investigational Product:	DEN-181 is a suspension of egg phosphatidylcholine (EPC) and egg phosphatidylglycerol (EPG) liposomes (approx. 100 nm diameter, lipid molar ratio 90:10) containing collagen II peptide and calcitriol in 2 mL amber glass vials for single use. Each vial contains 1.0 mL extractable volume of DEN-181 containing 0.6 µg/mL calcitriol and 45µg/mL collagen II peptide.
Comparator:	Placebo is sterile saline for injection (0.9% NaCl).
Duration of treatment per participant:	Screening: Up to 21 days<br>Study period: 56 days<br>Subjects will receive either: Part A- a single administration of DEN-181 with 56 days (8 weeks) of post treatment follow up or Part B- three administrations of DEN-181 at weekly intervals with 42 days (6 weeks) of post treatment follow up (on study period of up to 11 weeks for each participant from the start of Screening to the end of follow-up)

	Please refer to Table 1<br><br>This is a randomised, double blind, placebo-controlled, single centre, single-dose escalation and multiple dose, dose-ranging parallel group study to be performed in 2 parts.<br><br>Part A: Randomised double blind, placebo-controlled, single centre, single-dose escalation study at three dose levels;<br>Part B: Randomised double blind, placebo-controlled single centre multiple dose, dose- ranging parallel group study at two dose levels.<br><br>The 2 dose levels to be tested in the 2 groups of Part B will be based upon review of the data in respect of safety and immunomodulatory assessments of the three dose levels tested in Part A by a Safety Monitoring Committee (SMC).<br><br><u>Screening</u><br>Potential participants will be required to provide written informed consent prior to any study-specific screening procedures being performed.<br><u>Study Period</u><br>Eligible subjects will return at Baseline (Day 1) to be randomised to receive the first treatment<br>Part A<br>Up to 18 eligible participants will be randomised into Part A of the study. Three dose levels will be assessed in a single dose escalation study design Participants will be assigned to a dose level. Dose Level 1 (4 active, 2 placebo), Dose Level 2 (4 Active, 2 placebo), Dose Level 3 (4 active, 2 placebo). At each dose level two sentinel subjects (1 active, 1 placebo) will be initially randomised. The remainder of the participants will be dosed no earlier than 7 days after dosing the sentinel group. Safety and tolerability at each dose level will be assessed by a Safety Monitoring Committee<br>14 days after the dose given to the final cohort participant before proceeding to a progressively higher dose level.<br>Part B<br>Up to 40 eligible participants (30 active, 10 placebo) will be randomised into Part B of the study. The dose levels will be determined based on the results of Part A. Eligible subjects will be administered investigational medicinal product (IMP) on Days 1, 8 and 15 in Part B.
Study procedures:	Vital signs will be recorded prior to all Investigational Medical Product administrations and at 15 minute intervals for 30 minutes following each dose.<br>Vital signs will include supine blood pressure (systolic blood pressure (SBP) and diastolic blood pressure (DBP), heart rate, respiratory rate, and oral temperature.<br>Subjects will be required to remain in the clinic for at least 4 hours after all Investigational Product administrations. Blood will be drawn for limited PK analysis pre-dose and at 1, 2 and 4 hours post administration of IMP in both parts A and B.<br><br>Participants will be encouraged to visit the study site on the same day every week (± 2 days) to ensure the lengths of the weeks are similar<br>Physical examinations, adverse events, vital signs, clinical laboratory parameters (haematology, chemistry, urinalysis) and concurrent medications will be recorded throughout the study.<br>Serum pregnancy testing will be conducted for female participants at Screening and urine pregnancy tests before administration of each dose of IMP. If urine pregnancy testing is positive, a serum pregnancy test will be conducted and the IMP will not be administered until the result is available. If the serum pregnancy test is negative, administration of IMP will continue.

	If the result of the serum pregnancy test is positive, the participant will be withdrawn from the study and a Pregnancy Notification Form completed.<br>Blood samples will be collected and shipped to nominated laboratories for preparation of serum, plasma and peripheral blood mononuclear cells (PBMCs) for use in immunological analyses.<br>Subjects will be withdrawn from the study if deemed necessary by the PI if their condition cannot be sufficiently controlled with minor adjustments in treatment. All adjustments in treatment will be recorded.
Contraindications:	Severe local or systemic reactions that preclude further administration.
Safety parameters:	Refer to Table 1
Clinical procedures / assessments:	Refer to Table 1
Specialised analyses:	T-cell responses against RA autoantigens (e.g., MHC II tetramer analyses, tetramer-stimulated analyses for intra-cellular cytokines, ELIspot analyses)
Sample size determination:	No formal sample size calculation is proposed for this clinical trial. This clinical trial is a proof-of-principle study and results from this clinical trial will be utilised for power calculations for subsequent studies.
Statistical analyses:	Planned analyses: A statistical analysis plan (SAP) will be generated prior to database lock that describes the planned statistical analysis of the study. All data will be provided in data listings sorted by treatment group, time since baseline, and subject number. Categorical data will be summarised by the number and percent of subjects falling in each category. Continuous variables will be summarised by descriptive statistics including mean, standard deviation, median and minimum and maximum. Comparisons will be made between post-treatment and pre-treatment values in the DEN181-treated group and between DEN181-treated and placebo-treated groups. Relationships between DAS28CRPv4 and descriptive data will be determined in univariate and multivariate analyses. Multilinear regression models will assess longitudinal trends.<br>Safety will be assessed by summarising adverse events and clinical laboratory tests. Adverse events will be listed and summarised by system organ class and preferred terms (current version of MedDRA) per treatment group. Vital signs and clinical laboratory tests will be tabulated and summarised per treatment group. Adverse event severity will be graded according to NIH CTCAE Version 4.03.
Committees / guidelines:	DEN-181 Safety Monitoring Committee
Special protocol requirement:	None

Table 1-1 Table of Assessments

Assessment	Screening	On-Study Period				Exit Evaluation
	Day –35 to -1	D 1	D8	D15	D29	D57
	Visit 1	Visit 2	Visit 3	Visit 4	Visit 5	Visit 6
Written Informed Consent	X					
Eligibility Assessment/Confirmation	X	X				
Medical History	X					
Physical Examination (symptom directed)	X	X	(X)	(X)	(X)	(X)
Vitals Signs/Body Weight/Height [1,2,3,4]	X	X	X	X	X	X
Electrocardiogram	X					
Pregnancy Test [5] (Part B only)	X	X	(X)	(X)		X
HIV & Hepatitis Serology	X					
HLA Typing (if required)	X					
anti-CCP2	X				X	X
Rheumatoid Factor (RF)	X					
Issue Injection Site Reaction Diary (Part B only)		X	(X)	(X)		

Assessment	**Screening**	**On-Study Period**				**Exit Evaluation**
	**–35 to -1**	**D1**	**D8**	**D15**	**D29**	**D57**
	**Visit 1**	**Visit 2**	**Visit 3**	**Visit 4**	**Visit 5**	**Visit 6**
Review Injection Site Reaction Diary (Part B only)			X	(X)	(X)	
Haematology including ESR	X	X	X	X	X	X
Chemistry including CRP	X	X	X	X	X	X
Urinalysis	X	X	X	X	X	X
DAS28CRPv4 evaluation	X	X	X	X	X	X
PBMC processing and storage		X	X		X	
Tetramer Analysis (collagen II and HA)		X	X		X	
Exploratory immune and biomarker assays		X	X		X	X
Plasma Calcitriol assay[6] (Part B only)		X	(X)	(X)		
Oral swab; microbiome analysis		X				X
Dispense IMP (Part B only)		X	(X)	(X)		
Administration of IMP (Part B only)		X	(X)	(X)		
Adverse Event		X	X	X	X	X
Concurrent Medications Assessment	X	X	X	X	X	X

[1] Height and weight to be performed at Visit 1 only

[2] Vital signs include resting BP, heart rate, respiratory rate and oral temperature

[3] Safety assessments to be performed prior to IMP

[4] Vital signs to be performed prior to IMP administration and at 15 minutes and 30 minutes following dosing

[5] Serum pregnancy testing will be conducted in female participants of childbearing potential at Screening. Urine pregnancy testing will be conducted before each administration of IMP. If urine pregnancy test is positive, a serum pregnancy test will be conducted and the IMP will not be administered until the result of this is received. If the result of the serum pregnancy test is negative, administration of IMP will continue. If the result of a serum pregnancy test is positive, the participant will be withdrawn from the study.

[6] Blood collected for calcitriol assay pre-dose and at 1, 2 and 4 hours after IMP administration

OVERVIEW OF DEN-17-RA-01 PROTOCOL

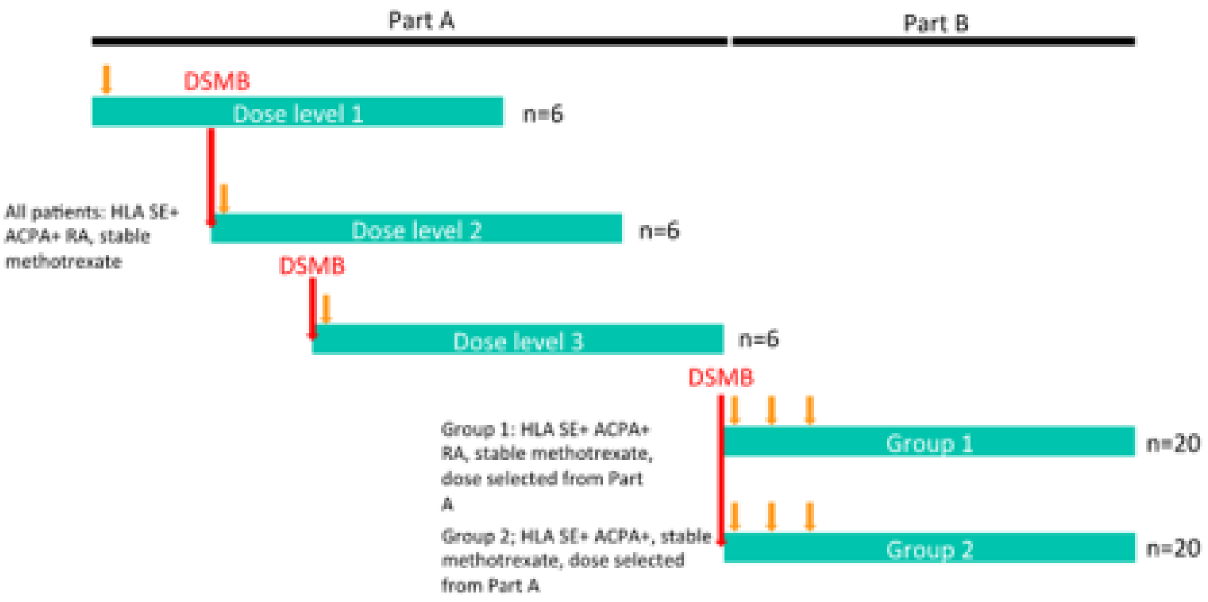

Figure 1 Diagram of study schedule

Table of Contents

1 LIST OF ABBREVIATIONS 4
2 STUDY CONTACTS 8
3 INTRODUCTION 10
3.1 Disease 10
3.2 DEN-181 – An Antigen-specific Tolerance Strategy 10
3.2.1 Pharmacology 11
3.2.1.1 In vitro pharmacology 11
3.2.1.2 In vivo pharmacology 12
3.2.2 Biodistribution 12
3.2.3 Preclinical safety 13
3.2.4 Pharmacokinetics and Toxicokinetics 13
3.3 Rationale for study and dose selection 14
4 OBJECTIVES AND ENDPOINTS 16
4.1 Objectives 16
4.1.1 Primary Objectives 16
4.1.2 Secondary Objectives 16
4.1.3 Exploratory Objectives 16
4.2 Endpoints 16
4.2.1 Primary endpoints: 16
4.2.2 Secondary endpoints: 16
4.2.3 Exploratory endpoints: 17
5 STUDY DESIGN 18
5.1 Study Design 18
5.2 Dosing regimens 19
5.3 Study sites 19
5.4 Estimated duration of the study 20
6 SUBJECT POPULATION 21
6.1 Selection and number of subjects 21
6.2 Inclusion criteria 21
6.3 Exclusion criteria 22
6.3.1 Part B Specific Inclusion criteria 22
6.4 Other study eligibility criteria considerations 22
6.4.1 Contraception 22
6.4.2 Renal Function 23
6.4.3 Rescreening 23
7 SCHEDULE OF ASSESSMENTS AND PROCEDURES 24
7.1 Study schedule of evaluations 24
7.2 Visit windows 24
7.3 Study procedures / assessment period 24
7.3.1 Visit 1 Screening visit 24
7.3.2 Visit 2 (Day 1) baseline evaluations, randomisation, and study medication dispensing 25
7.3.3 On-study clinic visits 26
7.3.3.1 Visit 3 (Day 8) 26
7.3.3.2 Visit 4 (Day 15) 26
7.3.3.3 Visit 5 (Day 29) 27
7.3.4 Visit 6 (Day 57) exit evaluation 28
7.4 Details of scheduled assessments 28
7.4.1 Demographic data, medical history, physical examination, vital signs 28
7.4.2 Electrocardiograms 28
7.4.3 Blood and urine samples for laboratory tests 29
7.4.4 Handling and processing of biological specimens 29
7.4.5 Diary booklets 30
7.4.6 Pregnancy tests 30
7.4.7 DAS28CRPv4 evaluation 31
7.4.8 Microbiome Testing 31
8 INVESTIGATIONAL MEDICINAL PRODUCT 32
8.1 Randomisation process 32
8.2 Blinding 32
8.3 Treatment allocation 33
8.4 Un-blinding 33
8.4.1 Medical emergency 33

8.4.2 End of study ... 33
8.5 Formulation ... 33
8.5.1 Investigational medicinal products ... 33
8.5.2 Supply, packaging and labelling, storage and handling ... 34
8.5.3 Dosage and administration of test drugs ... 35
8.5.4 Dispensing and accountability ... 35
9 CONCURRENT MEDICATIONS AND TREATMENTS ... 37
9.1 Special dietary requirements ... 37
9.2 Concurrent medications/treatments not permitted ... 37
9.2.1 Prior to study entry ... 37
9.2.2 During the study dosing period ... 37
10 ADVERSE EVENTS AND TOXICITY MANAGMENT ... 38
10.1 Safety parameters ... 38
10.2 Adverse events ... 38
10.2.1 Assessment of AEs ... 38
10.2.2 Adverse event reporting period ... 39
10.3 Serious adverse events ... 39
10.3.1 Serious adverse event definition ... 39
10.3.2 Clarification of serious adverse events ... 40
10.3.3 Serious adverse event reporting requirements ... 41
10.3.3.1 All SAEs ... 41
10.3.3.2 Investigator reporting requirements for SAEs ... 41
10.4 Follow up of serious and non-serious adverse events ... 41
10.5 Clinical Laboratory Abnormalities and Other Abnormal Assessments as AEs or SAEs ... 41
10.6 Toxicity Management ... 42
10.7 Guidance for dose modification or discontinuation of treatment ... 42
10.8 Warnings and precautions ... 43
10.9 Risks for women of childbearing potential or during pregnancy ... 43
10.10 Procedures to be followed in the event of pregnancy ... 43
11 DATA SAFETY MONITORING ... 45
12 SUBJECT COMPLETION/WITHDRAWAL ... 46
12.1 Subject completion ... 46
12.2 Criteria for premature withdrawal from treatment or the study ... 46
12.3 Withdrawal of subjects from study product ... 46
12.4 Withdrawal of subjects from the study ... 47
12.5 Replacement of withdrawn subjects ... 47
12.6 Premature termination of the study ... 47
13 STATISTICAL ANALYSIS ... 48
13.1 Hypothesis ... 48
13.2 Sample size determination ... 48
13.3 Randomisation ... 48
13.4 Criteria for evaluation of study objectives ... 48
13.4.1 Definition of evaluation of safety ... 48
13.4.2 Definition of evaluation of secondary study objective(s) ... 48
13.4.3 Analysis populations ... 49
13.4.3.1 Group comparability ... 49
13.4.3.2 Data analysis methods ... 49
13.5 Statistical and analytical plan ... 49
13.6 Final analyses ... 50
13.6.1 Analysis of demographics ... 50
13.6.2 Analysis of Immunomodulation ... 50
13.6.3 Analysis of safety ... 50
13.6.4 Adverse events ... 50
13.6.5 Vital signs, laboratory evaluations and DAS28CRPv4 ... 50
13.6.6 Other exploratory biomarkers and analysis of immunomodulatory effects ... 50
13.6.7 Analysis of calcitriol levels in plasma ... 51
13.7 Interim analysis ... 51

13.8 Analysis of Part A Data and Dose selection for Part B ... 51
14 GENERAL STUDY ADMINISTRATION ... 52
14.1 Ethical aspects ... 52
14.1.1 Local regulations/declaration of Helsinki ... 52
14.1.2 Informed consent ... 52
14.1.3 Premature withdrawal ... 52
14.1.4 Institutional review boards or ethics committees ... 52
14.1.5 Conditions for modifying the protocol ... 52
14.1.6 Conditions for terminating the study ... 53
14.2 Study documentation, CRFs and record keeping ... 53
14.2.1 Investigator’s files/retention of documents ... 53
14.2.2 Background data ... 54
14.2.3 Audits and inspections ... 54
14.2.4 Case report forms ... 54
14.3 Monitoring the study ... 54
14.4 Confidentiality of trial documents and subject records ... 54
14.5 Publication of data and protection of trade secrets ... 55
14.6 Anticipated subject accrual and duration of the study ... 55
15 REFERENCES ... 56
16 APPENDICES ... 57
16.1 Appendix 1 NCI CTCAE v4.03 ... 57
16.2 Appendix 2 DAS28CRPv4 Scoring Tool and Calculator ... 58

1 LIST OF ABBREVIATIONS

ABBREVIATION	DESCRIPTION
ACPA	Anti-Citrullinated Peptide Antibody
AE	Adverse Event
ADR	Adverse Drug Reaction
ALDH	(retin)aldehyde dehydrogenase
ALP	Alkaline Phosphatase
ALT	Alanine Transaminase
AST	Aspartate Aminotransferase
Beta-HCG	Beta Human Chorionic Gonadotropin
BMI	Body Mass Index (Weight in kg divided by height in $m^{2)}$
BP	Blood Pressure
cGMP	Current Good Manufacturing Practice
C_{max}	Observed maximum concentration
CBC	Complete Blood Count
CCP	Cyclic citrullinated peptide
CI	Confidence Interval
cit	citrullinated
CMI	Cell-mediated Immunity
CMV	cytomegalovirus
COA	Certificate of Analysis
CII	Collagen type II protein
CII Peptide	Peptide corresponding to amino acids 259 to 273 of human Collagen II with hydroxyproline in the 273 position. Amino Acid sequence: GIAGFKGEQGPKGE-Hyp
CRF	Case Report Form
CRP	C-reactive protein

ABBREVIATION	DESCRIPTION
CRO	Clinical Research Organisation
CSR	Clinical Study Report
CTN	Clinical Trial Notification
d	Day
DAS28CRPv4	Disease Activity Score for 28 joints plus CRP assessment (4variable calculation)
DBP	Diastolic Blood Pressure
DC	Dendritic Cell
EBV	Epstein-Barr Virus
ECG	Electrocardiogram
EPC	Egg Phosphatidylcholine
EPG	Egg Phosphatidylglycerol
ELISA	Enzyme-linked immunosorbent assay
ELISPOT	Enzyme-linked immunospot assay
ESR	Erythrocyte sedimentation rate
FBC	Full Blood Count
GCP	Good Clinical Practice
GGT	Gamma Glutamyltransferase
GLP	Good Laboratory Practice
GMP	Good Manufacturing Practice
HA peptide	Influenza Hemagglutinin peptide aa306-318
Hb	Haemoglobin
Hct	Haematocrit
HIV	Human Immunodeficiency virus
HED	Human Equivalent Dose
Hep B	Hepatitis B
Hep C	Hepatitis C

ABBREVIATION	DESCRIPTION
HLA-DR	An MHC class II surface receptor encoded by the human leukocyte antigen complex
HR	Heart Rate
HREC	Human Research Ethics Committee
IB	Investigator's Brochure
IFNγ	Interferon γ
ICH	International Council on Harmonisation of Technical Requirements for Registration of Pharmaceuticals for Human Use
IMP	Investigational medicinal product
Inj	Injection
IUD	Intrauterine device
ITT	Intention to Treat
LN	Lymph nodes
LFT	Liver Function Test
mcg	Microgram
MHC II	Major Histocompatibility Complex Class II
MTX	Methotrexate
NHMRC	National Health and Medical Research Council
NIH CTCAEv4.03	National Institutes of Health Common Terminology Criteria for Adverse Events v4.03
NOAEL	No Observable Adverse Effect Limit
PBMC	Peripheral blood mononuclear cells
PUVA	Psoralen and Ultraviolet A Radiation
RA	Rheumatoid Arthritis
RT	Room temperature
SAE	Serious Adverse Event
SAP	Statistical Analysis Plan

ABBREVIATION	DESCRIPTION
SC	Subcutaneous
SMC	Safety Monitoring Committee
SOC	System Organ Class
SUSAR	Serious Unexpected Serious Adverse Reaction
TEAE	Treatment Emergent Adverse Event
Teff	T effector cells
Tfh	T follicular helper
T_{max}	Time of observed maximum concentration
Treg	T regulatory cells
Trm	T resident memory
TG	transgenic
TGA	Therapeutic Goods Administration

2 STUDY CONTACTS

STUDY SPONSOR
Dendright Pty. Ltd.
Registered Office: Level 7, General Purpose South Building
Staff House Road
The University of Queensland
St. Lucia, 4067
AUSTRALIA

PRINCIPAL INVESTIGATOR
Dr Phillip Vecchio MBBS (Hons) FRACP, MRACMA, MBA, GCEd,
Department Head of Rheumatology
Princess Alexandra Hospital
Ipswich Road
Woolloongabba, 4102
AUSTRALIA

STUDY MEDICAL MONITOR
Dr John Gillies MBChB, FRACP, FRCPC, FAAP
Clinical Network Services (CNS) Pty Ltd
Level 4, 88 Jephson Street
Toowong, 4066
AUSTRALIA

INVESTIGATIONAL SITE
Translational Research Institute Clinical Research Facility
Princess Alexandra Hospital
Ipswich Road
Woolloongabba, 4102
AUSTRALIA

SPONSOR SIGNATORY
Helen Roberts MBA
Chief Executive Officer
Dendright Pty Ltd
Level 7, General Purpose South Building
Staff House Road
The University of Queensland
St. Lucia, 4067
AUSTRALIA

LABORATORY ANALYSIS (safety bloods)
Pathology Queensland
Princess Alexandra Laboratory
Princess Alexandra Hospital
Ipswich Road
Woolloongabba, 4102
AUSTRALIA

HLA-typing (Screening – only if required**)**
Victorian Red Cross
23-47 Villiers Street
North Melbourne, 3051
AUSTRALIA

PHARMACODYNAMIC ANALYSIS (FACS phenotyping of peripheral blood)
Precision for Medicine
8425 Progress Drive
Frederick, MD 21701
USA

PHARMACOKINETIC ANALYSIS (calcitriol concentration in plasma)
Eurofins ADME BIOANALYSES
75 Chemin de Sommières
30310 Vergèze,
FRANCE

ADDITIONAL EXPLORATORY IMMUNE ANALYSES
Thomas Laboratory
UQ Diamantina institute
Translational Research Institute
37 Kent Street
Woolloongabba, 4102
AUSTRALIA

3 INTRODUCTION

3.1 Disease

Rheumatoid arthritis (RA) is an incurable autoimmune disease affecting 1 in 100 adults worldwide. RA reduces survival by an average of 10 years due to cardiovascular complications [1]. Seropositive RA is linked to the *HLA-DRB1* high risk allele, and is associated with the presence of rheumatoid factor (RF) and/or anti-citrullinated peptide antibodies (ACPA). *HLA-DRB1* alleles share a common motif called the "shared susceptibility epitope (SE)". The SE accommodates self-epitopes for presentation by antigen presenting dendritic cells (DCs) to the immune system [2-4]. HLA alleles associated with ACPA-positive RA, include HLA-DRB1*04:01 and *01:01. In the immediate pre-RA period, ACPA isotype diversity and titre have been shown to increase – a process associated with antigen-specific CD4+ T cell help for affinity maturation of antibody in germinal centres [5]. *HLA-DR SE* are associated with ACPA+ RA rather than presence of ACPA, implying that presentation of Cit-autoantigens bound to HLA-DR SE molecules to CD4+ T cells is associated with diversification and amplification of ACPA as well as RA development in at-risk individuals carrying *HLA-DR SE*.

Collagen type II (CII) is exclusively expressed in articular cartilage. Mice, rats and monkeys immunized with bovine, chick or human CII develop collagen-induced arthritis (CIA). CII-specific autoreactive CD4 T cells and B cells have been demonstrated in mice and humans. The dominant T cell epitope ($CII_{259-273}$) has been elucidated in HLA-DRB1*04:01 and HLA-DRB1*01:01 transgenic (TG) mice immunized with bovine or human CII [6, 7]. The minimal T cell epitope is mapped to positions 262-270 [8]. Phenylalanine 263 and the negatively charged glutamate 266 provide strong anchors at specific sites in the HLA-DR-SE binding site [7]. T cells from DRB1*04:01^{+} RA patients have been shown to respond to the dominant collagen II epitope as well as post-translationally modified epitopes [9]. In HLA-DR4 TG mice, the native rather than glycosylated CII epitope was immunodominant [6]. We have demonstrated collagen II peptide-specific CD4+ T cells in peripheral blood (PB) of DRB1*04:01^{+} and DRB1*01:01^{+} RA patients, first-degree relatives and healthy controls, using specific major histocompatibility type II (MHC II) tetramer staining reagents.

Multiple forms of CII tolerising immunotherapy have been demonstrated to suppress disease severity and T cell responses in H-2q, DR1-TG and DR4-TG mice, including tolerogenic DCs and $collagen_{259-273}$-HLA-DR4 coated nanoparticles [10-12]. CII-specific autoantibodies are directly pathogenic upon transfer to mice [13]. This occurs through complement fixation and direct binding of the autoantibodies to articular cartilage, leading to a local inflammatory response which models the effector phase of autoimmune arthritis. Autoantibodies recognizing an immunodominant conformational epitope known as $C1^{III}$ (residues 359-369) were significantly elevated in early RA patients compared to healthy controls, and titres were higher in HLA-DR-SE patients [14].

In summary, the development of ACPA+ RA in individuals carrying a specific set of high-risk HLA-DR alleles, the identification of dominant self-epitopes binding to and presented to T cells by those HLA-DR molecules, and their role in RA autoimmune pathogenesis sets the stage to specifically modulate the immune response towards immunodominant self-epitopes in favour of tolerance using antigen-specific tolerance strategies.

3.2 DEN-181 – An Antigen-specific Tolerance Strategy

Current treatments to control pathological immune responses during autoimmunity use broadly immunosuppressive drugs which can be associated with undesirable side effects. Alternative strategies to induce antigen-specific immunological tolerance to control both cellular and humoral immune responses are desirable.

Antigen-directed immunomodulation of antigen-presenting cells to induce immune regulation has previously been evaluated in the setting of RA. In a phase 1 clinical trial, the safety and immune effects of intradermally-injected autologous DCs, modified ex vivo through exposure to an NF-kB inhibitor and citrullinated peptides were assessed. This treatment reduced circulating effector T cells and increased the ratio of regulatory to effector T cells in RA [15].

To simplify and operationalise antigen-directed immunomodulation, Dendright has developed a strategy for passive targeting of antigen presenting cells (APC) in situ after systemically administering a nanoparticle-based immunotherapy (DEN-181), which exerts pharmacological effects on target immune cells following uptake. DEN-181 is a subcutaneously administered combination drug product comprising liposomal nanoparticles encapsulating a fixed dose of two biomolecules: (1) 1,25-dihydroxyvitamin D3 (calcitriol), an endogenous vitamin D compound and known inhibitor of NF-kB activation, and (2) a non-conformational linear peptide corresponding to an RA-associated peptide self-antigen, the 259 - 273 region of human collagen II protein.

The hypothesis which underlies the rationale for DEN-181 therapy is that APC uptake and cellular processing of the active components of DEN-181 will lead to immune modulation/regulation of activated collagen II-specific effector T cells resulting in clinical benefit. Theoretically, DEN-181 should offer higher specificity of effect and lower toxicity than current treatments for RA in HLA-DRB*0401+ and *0101+ individuals, given the lack of broadly suppressive effects on the immune system. To study the effects of DEN-181 therapy on immune cell populations, tetramer-based immune-monitoring assays involving staining, enumeration and phenotypic evaluation of antigen-specific and non-antigen specific cells will be conducted.

Included below is a summary of the preclinical and clinical profile of DEN-181. More detailed information is available in the DEN-181 Investigator’s Brochure.

3.2.1 Pharmacology

A range of *in vitro* and *in vivo* pharmacodynamic studies have demonstrated that liposomal nanoparticles co-delivering an antigenic peptide and calcitriol are taken up by target DCs and are able to modulate DC phenotype and bias the antigen-specific T effector cell / T regulatory cell ratio towards the induction of antigen-specific T cell tolerance in model immunologic systems.

3.2.1.1 In vitro *pharmacology*

In vitro uptake studies have demonstrated that liposomes co-delivering antigenic peptide and calcitriol are taken up by target immune cells, particularly DCs. Calcitriol-containing liposome formulations inhibit the transcription factor, NF-κB, in isolated human DCs to induce a suppressive phenotype. Specifically, retinaldehyde-specific dehydrogenase (ALDH) expression is increased and co-inhibitory and regulatory markers are upregulated by (CD1c+) DCs from human RA PBMCs. Treatment with calcitriol–liposomes containing CII peptide induced CII peptide-specific T cell responses in human RA PBMCs, including upregulated expression of markers of antigen experience: PD1, CD40-L, CD69 and CD25.

3.2.1.2 **In vivo** ***pharmacology***

In vivo studies have utilised liposome treatments encapsulating CII peptide as well as liposomes containing various model antigens relevant to specific animal models allowing investigation of human autoimmunity. This is because the human T cell epitope contained in DEN-181 and to be used in clinical studies, is not immunogenic in standard animal models. Opportunities to assess the *in vivo* pharmacology of DEN-181 are limited to transgenic (TG) mice incorporating HLA-DRB1. In various other nonclinical studies, calcitriol-containing liposomes were formulated without peptide or with ovalbumin (OVA) peptide corresponding to position 323-339 of the OVA protein sequence ($OVA_{323-339}$), or with aggrecan peptide corresponding to position 89-103 of the aggrecan protein ($Aggrecan_{89-103}$). These liposomal formulations have been used to demonstrate induction of antigen-specific T cell tolerance following adoptive transfer of T cells in DO11 mice responsive to $OVA_{323-339}$, and PGIA-induced arthritis in mice primed/boosted with human aggrecan (proteoglycan) protein.

In vivo uptake studies have demonstrated that liposomes co-delivering antigenic peptide and calcitriol delivered SC are taken up by target immune cells, particularly DCs, which in turn present peptide to autoreactive T cells.

Preclinical studies of DEN-181 demonstrate that after SC injection, a proportion of the dose rapidly disperses via afferent lymphatics to draining lymph nodes (LN), where liposomes carrying antigen and calcitriol payloads are taken up by skin migratory DCs and – under inflammatory conditions – by inflammatory DCs. Antigen-specific Treg significantly increased within 6 days of a single administration of calcitriol-OVA liposomes and increased further after 2 injections of calcitriol-OVA liposomes. After subcutaneous administration of calcitriol-OVA liposomes, expression of regulatory and costimulatory molecules including ALDH, PDL1, CD40 and CD86, increased in draining LN DCs taking up liposomes. Antigen-specific T cells responding to delivered antigen expressed the PD1 receptor, which is known to down-regulate T cell function. After liposome-mediated induction of Treg, expansion and cytokine production of a new cohort of antigen-specific effector T cells was suppressed in vivo, consistent with an “infectious” process whereby memory differentiation of antigen-specific T cells is regulated by antigen-specific Treg. In support of this conclusion, in a preclinical model of proteoglycan antigen-induced inflammatory arthritis, antigen-specific suppression of disease severity by calcitriol-aggrecan peptide liposomes was correlated with an increased proportion of aggrecan-specific T cells with a naïve phenotype. Reduced proportions of aggrecan-specific effector populations including follicular-helper T cells (Tfh) were observed after treatment with calcitriol-aggrecan peptide but not calcitriol-OVA peptide liposomes.

3.2.2 Biodistribution

Tissue distribution following subcutaeous administration of a liposome formulation, in which the lipid was trace labelled with ^{14C}-DPPC, and loaded with ^{3H}-labelled calcitriol was evaluated in mice. The study sought to investigate the fate of calcitriol and liposomes upon SC administration and determine whether calcitriol was retained by the liposomes. The disposition of lipid can also be viewed as a surrogate for peptide localisation, as the high hydrophilicity of the peptide and encapsulation in the liposomal core ensures it is unlikely to partition to/through the lipid bilayer until the liposome shell is degraded.

Most of the radioactivity attributable to ^{3H}-calcitriol was retained at the injection site. This may indicate that calcitriol largely stays with the liposomes at the site of injection. Calcitriol entered the blood stream more avidly than the liposomes indicating some separation of the calcitriol from the liposomes. However, the amount of radiolabel entering the bloodstream was low and the drug was largely confined to the plasma component. Distribution of liposomes to draining lymph nodes is expected to be the clearance mechanism for intact liposomes and encapsulated drug and this was evidenced by levels of 5-10% of dosing counted in the draining LN within 5 minutes of administration.

3.2.3 Preclinical safety

A GLP-compliant 22-day repeated dose toxicity study in the Sprague-Dawley rat was undertaken to assess the toxicity and toxicokinetic profile of DEN-181 following 4 once-weekly subcutaneous injections (Days 1, 8, 15 and 22) at low, intermediate and high dose calcitriol (i.e., 0.06, 0.18, and 0.6 µg/animal/dose) and CII peptide (i.e.,4.5, 13.5, and 45 µg/animal/dose). The objective was to assess the persistence, delayed onset or reversibility of any test item-related effects following a 14-day recovery period. Vehicle control, liposomal control, liposomal peptide control, and liposomal calcitriol control items were also administered subcutaneously to groups of rats once weekly (Days 1, 8, 15, and 22).

All main and recovery animals survived until their respective scheduled termination (Day 23 and 36, respectively). Once-weekly subcutaneous injections of DEN-181 at all dose levels in Sprague-Dawley rats for 4 weeks was tolerated and did not result in any signs of overt toxicity. There were no DEN-181-related clinical observations, neurological, autonomic or behavioural effects, nor changes in body weights, food consumption, ophthalmology, hematology, coagulation, urinalysis, organ weights, and macroscopic evaluations that could be attributed to the subcutaneous injection of DEN-181 at any dose level.

Non-adverse dose-related increase in serum calcium and phosphorus levels were noted in DEN-181-treated animals, as well as in liposomal calcitriol control animals at calcitriol levels up to 0.6µg/animal/dose, but reversed following 14 days of recovery. Microscopically, minimal to moderate renal tubular mineralization in males and females, and minimal mineralization in the stomach glandular mucosa of males were noted, and were attributed to the pharmacodynamic effect of calcitriol up to 0.6 µg/animal/dose with or without collagen II peptide up to 45 µg/animal/dose. The kidney and stomach lesions in males persisted at 0.6µg/animal/dose of calcitriol (DEN-181 and liposomal calcitriol controls) through the recovery period, while kidney lesions in the females demonstrated reversibility following 14 days of recovery. At this dose, mineralization of renal tubules in males and females and mineralization of the glandular stomach in males were observed, but were minimal in severity, and therefore, considered non-adverse.

Based on these observations and the mild severity of microscopic findings, the no observed adverse effect level (NOAEL) for DEN-181 was considered to be 0.18 µg/animal/dose of calcitriol with 13.5 µg/animal/dose of collagen II peptide.

3.2.4 Pharmacokinetics and Toxicokinetics

Following a single subcutaneous dose of DEN-181 at 100, 300 and 1000 µL in the Sprague Dawley Rat, the maximal plasma concentrations of calcitriol (C_{max}) of 1.94 ± 0.04 ng/mL, 4.50 ± 0.23 ng/mL and 10.54 ± 0.64 ng/mL were reached at 3.3, 4 and 4 hours (T_{max}) after dosing, respectively. Analysis of vehicle-treated controls revealed endogenous calcitriol levels of 211 – 333 pg/mL.

The toxicokinetics of DEN-181 were assessed in Sprague-Dawley rats treated with DEN-181 (at low, intermediate or high dose), once weekly (on Day 1, 8, 15 and 22) over 22 days. Samples for toxicokinetic analysis were collected at Day 1 and Day 22. C_{max} was 1.2 to 1.6 ng/mL for the lowest dose, 2.5 to 2.9 ng/mL for the intermediate dose, and 6 to 9 ng/mL for the highest dose with no significant difference between Day 1 and Day 22, and no gender difference observed. While at Day 1, C_{max} was measured 2 hours after administration, generally T_{max} was slightly later at Day 22 (2 – 6 hours). AUC_t was a slightly lower value for males than for females after low dose delivery (17.80 ng/mL*h to 27.16 ng/mL*h), but there was no difference between Day 1 and Day 22. No difference between genders or Day 1 and Day 22 was observed for AUC_t at the intermediate dose (38.02 to 42.20 ng/mL*h). At high doses, females showed higher AUC_t than males (87.00 to 114.1 ng/mL*h). C_{max} and AUC_t showed a less than proportional increase in function of the administered doses for both days and genders. This may indicate a depot like effect, wherein liposomes are retained at the

site of subcutaneous injection for over 24 hours. However, the ratios of C_{max} and AUC_t on Day 1 and Day 22 indicate that there is no accumulation of the drug after repeated dose.

At the NOAEL, exposures to calcitriol (represented by the mean C_{max} and AUC_t) on Day 22, was approximately 2.5 ng/mL and 38.0 hr*ng/mL, respectively, in males and approximately 2.7 ng/mL and 40.0 hr*ng/mL, respectively in females.

3.3 Rationale for study and dose selection

Current RA treatments include disease modifying anti-rheumatic drugs, such as MTX, and inhibitors of TNF and IL-6, as well as biologic therapies such as CTLA4-Ig and anti-CD20, which reduce symptoms of RA and pathological tissue inflammation. However, these treatments are associated with adverse effects, are not curative, and fail to fully control disease in the majority [16].

DEN-181 is designed to target the dysregulated immune system that underlies autoimmunity to specific RA-associated self- antigens. DEN-181 is a novel fixed dose combination drug product which targets cells of the immune system through liposomal delivery. Uptake and cellular processing of DEN-181 by target immune cells is intended to re-program the immune system towards immune tolerance and deliver improved patient outcomes and minimal side effects.

Clinicians believe that there is a chance to change the course of disease if effective treatments are used early in the disease process before immune system mediated damage to organs and tissues becomes chronic. DEN-181 is the first therapeutic approach to specifically target ACPA+ patients carrying specified MHC II HLA-DRB1 RA-susceptibility gene variants to reprogram their immune system towards tolerance to a specific self-antigen, thereby addressing underlying autoimmunity and promoting long-term remission. DEN-181 will be specifically targeted to ACPA+ RA patients who are positive for the shared HLA susceptibility epitopes (SE+) 0401 and 0101.

Dendright plans to conduct a two-stage phase 1b clinical trial study with DEN-181 in clinically relevant populations of RA patients to investigate safety, tolerability, immunomodulatory effects and preliminary efficacy of the product. The study will test the hypothesis that co-delivery of an NF-κB inhibitor with a self-antigen in a liposome will promote antigen-specific immune tolerance.

Therefore, in the current trial of DEN-181 we will evaluate whether DEN-181, delivered subcutaneously, will:

- Reduce the frequency of collagen II peptide-specific CD4 T cells in peripheral blood relative to baseline and relative to placebo-dosed patient samples. We posit that HA-specific CD4 T cells in peripheral blood should remain unchanged;
- Increase the proportion of collagen II peptide-specific CD4 T cells with a naïve ($CD45RA^+$) phenotype and decrease collagen II peptide-specific CD4 T cells with (i) memory phenotype ($CD45RO^{+)}$, (ii) activated phenotype ($HLA\text{-}DR^+$, $CD40L^+$) and differentiated effector phenotypes including tissue resident memory (Trm $CD45RO^+CCR7^+CD69^+$), Tfh,($CCR7^{lo}PD1^{h}iCXCR5^+$) Th17 ($CCR6^+$) or Th1 ($CXCR3^+$) cells relative to baseline and relative to placebo-dosed patients, while HA-specific CD4 T cells will remain unchanged;
- Increase PB collagen II-specific CD4 T cells with a Treg ($CD25^{hi}CD127^-$) phenotype and expression of effector Treg markers (CD45RO, CD15s, TIGIT, PD1, CD73) and increase the ratio of PB collagen II-specific $CD25^{hi}CD127^-/CD25^+CD127$ CD4+ T cells relative to baseline and relative to placebo-dosed patients, while HA-specific CD4 T cells will remain unchanged.

DEN-181 is an immunomodulatory combination drug product which mediates its effect through modulation of immune cells in primary lymphoid tissue to generate a system-wide immunological effect.

In mouse models of antigen-specific tolerance, it has been demonstrated that calcitriol-peptide liposomal formulations delivering 0.4-0.6 µg/mL calcitriol and 10-50 µg/mL peptide in an administered volume of 100 µL generated a biological effect. Moreover, in a toxicity study in rats assessing four administrations of once-weekly DEN-181, the no observed adverse effect level (NOAEL) for DEN-181 was considered to be a dose of 0.18 µg/animal/dose of calcitriol with 13.5 µg/animal/dose of collagen II peptide. The average calcitriol dose relative to body weight at the NOAEL was 0.45 and 0.71 µg/kg for males and females respectively, or 0.58 µg/kg for the combined sexes. This average dose is equivalent to a human dose of 0.093 µg/kg, after interspecies scaling relative to body surface area. The proposed maximum dose of calcitriol in DEN-181 in the clinical trial is 0.6 µg, or 0.0086 µg/kg for a 70-kg person, which is approximately 11 times lower than the human equivalent dose of calcitriol at the rat NOAEL.

We have proposed a starting dose of 0.1mL DEN-181 (4.5µg collagen II peptide, 0.06µg calcitriol) and highest dose of 1.0mL DEN-181 (45µg collagen II peptide, 0.6µg calcitriol) for this study based on the adequate safety margin seen in rat toxicology studies as outlined above (see Investigator’s Brochure for additional detail).

4 OBJECTIVES AND ENDPOINTS

4.1 Objectives

4.1.1 Primary Objectives

The primary objective of this study is:

- To determine the safety and tolerability of single and multiple doses of DEN-181 in patients with ACPA+ RA.

4.1.2 Secondary Objectives

The secondary objectives of this study are:

- To determine the ability of DEN-181 to modulate the total T-naïve, T-effector and T-regulatory cell count in peripheral blood;
- To determine the antigen-specific immune response to DEN-181 including the determination of collagen II peptide-specific T-naïve, T-effector and T-regulatory cells;
- To determine preliminary clinical efficacy of DEN-181 in MTX-treated early ACPA+ RA patients;
- To monitor plasma concentrations of calcitriol.

4.1.3 Exploratory Objectives

The exploratory objectives are:

- To investigate the effects of administration of DEN-181 on immune responses, disease biomarkers and the microbiome using various exploratory assays in blood, urine and gastrointestinal tract.

4.2 Endpoints

4.2.1 Primary endpoints:

- Clinical safety observations including changes in vital signs, adverse events, serious adverse events, laboratory abnormalities and withdrawals from study. Adverse event severity will be graded according to CTCAE, Version 4.03. Any exacerbation of RA will be evaluated by using the DAS28CRPv4 scoring system.

4.2.2 Secondary endpoints:

- Proportion, total number and phenotype of naïve, effector and regulatory T-cells measured by flow cytometry and MHC II tetramer analysis pre- and post-administration of DEN-181;
- Mean changes in DAS28CRPv4 from baseline in treatment and control groups;
- Determination of the concentration of calcitriol in plasma.

4.2.3 Exploratory endpoints:

Various exploratory immune assays. These measures may include, but are not limited to measures of humoral and T-cell responses such as the following:

- Proportion and phenotype of naïve, effector and regulatory T-cells specific to other autoantigens (e.g., responses to other autoantigens including cit-vimentin);
- T-cell responses as determined by interferon-gamma (IFN-γ) enzyme linked immunospot (ELIspot) assays to common infectious antigens (CMV, EBV, influenza);
- CD4 T-cell responses as measured by intracellular cytokine staining (ICS) (e.g., IL-2 and IFN-γ);
- Antibody titres of RA autoantigens including collagen, modified collagen and ACPA;
- Ex-vivo re-stimulation assays (e.g., as measured by cytokine secretion or tetramer analysis);
- T-cell receptor type as measured by sequence analysis on specific single cell sorted cellular populations.

Biomarker readouts may include, but are not limited to the following:

- cytokine levels;
- tryptophan/kynurenine levels (HPLC);
- metabolomics in urine;
- transcriptome analysis;
- microbiome analysis.

5 STUDY DESIGN

5.1 Study Design

A Phase I, randomised, double-blind, placebo-controlled, single-centre, single-dose escalation and multiple dose, dose-ranging parallel group study to investigate the safety, tolerability and pharmacodynamics of subcutaneously administered DEN-181 in adult patients with ACPA+ rheumatoid arthritis on stable treatment with methotrexate. The study is to be performed in two parts.

Part A: A randomised double blind, placebo-controlled, single centre, single dose escalation study.

Up to 18 eligible participants will be randomised into Part A of the study. Three dose levels will be assessed in a single dose escalation study design.

Participants will be assigned to a dose level. Dose Level 1 (4 active, 2 placebo), Dose Level 2 (4 active, 2 placebo), Dose Level 3 (4 active, 2 placebo). At each dose level two sentinel subjects (1 active, 1 placebo) will be initially randomised. The remainder of the participants in each dose level will be dosed no earlier than 7 days after the sentinel patients. The decision to continue dosing will be made upon review of individual safety data available from both participants at visit 2 (D8) by the Investigator. The Investigator may confer with members of the Safety Management Committee (SMC) if warranted. Overall assessment of safety and tolerability at each dose level will be made by the SMC by review of available safety results in aggregate from each cohort. A dose-escalation decision will be made no earlier than 14 days after the dose given to the final cohort participant before proceeding to a progressively higher dose level.

Decision rules and conduct of the SMC will be described in the SMC Charter Document.

Part B: Randomised double blind, placebo-controlled single centre multiple dose, dose-ranging parallel group study at two dose levels.

Up to 40 eligible participants will be randomised into two groups, Group A (15 active, 5 placebo) and Group B (15 active, 5 placebo). Eligible subjects will be administered IMP on Days 1, 8 and 15.

The dose levels to be tested in Part B will be based upon review of aggregated data for safety and immunomodulatory effects, if any, for all dose levels tested in Part A by the Safety Monitoring Committee.

Study participants in Part B will receive three administrations of IMP at weekly intervals (Day 1, Day 8, Day 15). Dose levels will be selected from 100µL, 300uL or 1000µL DEN-181 containing 0.6 µg/mL calcitriol and 45µg/mL collagen II peptide. Doses will be administered by subcutaneous injection.

Study participants in both Parts A and B will be monitored for 56 days after first administration of IMP.

There are two primary reasons for inclusion of the placebo groups:

- To maintain the blind; and
- To describe any natural variation in immunological readout pre- and post- administration of the placebo; and
- To serve as a comparator for safety and tolerability assessments.

5.2 Dosing regimens

Part A

Group 1

- Single 100µL subcutaneous injection of DEN-181 containing 0.6 µg/mL calcitriol and 45µg/mL collagen peptide (4 patients);
- Single 100µL subcutaneous injection of placebo containing sterile saline for injection (2 patients).

Group 2

- A total of 300µL given by two 150µL subcutaneous injections of DEN-181 containing 0.6 µg/mL calcitriol and 45µg/mL collagen peptide (4 patients);
- A total of 300µL given by two 150µL subcutaneous injections of placebo containing sterile saline for injection (2 patients).

Group 3

- A total of 1000µL given by four 250µL subcutaneous injections of DEN-181 containing 0.6 µg/mL calcitriol and 45µg/mL collagen peptide (4 patients);
- A total of 1000µL given by four 250µL subcutaneous injections of placebo containing sterile saline for injection (2 patients).

Part B

Study participants will receive three administrations of IMP at weekly intervals (Day 1, Day 8, Day 15). There will be two patient cohorts in Part B of the study with each of these groups receiving a different dose level of DEN-181. 15 patients will receive DEN-181 and 5 patients will receive placebo. Up to 40 patients will be enrolled in Part B of the study. The Dose levels to be utilised will be either 100µL or 300µL or 1000µL subcutaneous injection of DEN-181 containing 0.6 µg/mL calcitriol and 45µg/mL collagen peptide. Final dose selection will be based upon results from Part A of the study with pre-defined selection criteria by a Safety Monitoring Committee.

For both Parts A and B, the different dosage volumes of IMP will be administered in the following way:

- Subjects will receive 100µl subcutaneous injections approximately 10 cm above the elbow joint overlying the deltoid muscle of the left upper arm. Where possible the same arm will be injected throughout the course of the study.
- For 300µl subcutaneous injections, 150µl will be delivered 10 cm above the elbow joint overlying the deltoid muscle of each upper arm.
- For 1000µl subcutaneous injections, 250µl will be delivered 10 cm above the elbow joint overlying the deltoid muscle of each upper arm and approximately 5 cm from the groin on each upper thigh.

5.3 Study sites

This is prospectively designed as a single centre study but may be revised to a multi-centre study after the initial rate of recruitment is assessed by the Sponsor.

5.4 Estimated duration of the study

Part A

It is anticipated that the duration of Part A will be 8 months:

- 5 months for screening and the on-study period;
- 3 months for sample analysis and data review to support selection of dose levels to be employed in Part B.

Part B

It is anticipated that the duration of Part B will be 12 months:

- 8 months for screening and the on-study period;
- 4 months for sample analysis and study close out.

6 SUBJECT POPULATION

The study population will be patients with ACPA+ rheumatoid arthritis on stable treatment with methotrexate. Any swollen joint count at screening is acceptable (0-28). Patients will be asked about the frequency and duration of intermittent joint swelling. The study population in Part B will restrict patients to diagnosis of RA within the last 2 years.

The nature of the study and the potential risks will be explained to all candidates. Written informed consent will be obtained from each subject prior to performing screening procedures.

Following these assessments, subjects who meet all of the inclusion and none of the exclusion criteria will be eligible to continue in the study.

There will be no exemptions and subjects must satisfy all eligibility criteria in order to participate.

6.1 Selection and number of subjects

Part A

After screening, up to 18 eligible subjects will be enrolled and randomly assigned to DEN-181 or placebo. Sentinel dosing will be included in each dose cohort;

Part B

After screening, up to 40 eligible subjects will be enrolled and randomly assigned to DEN-181 or placebo.

The randomisation numbers will be generated as described in Section 8.1

Subjects enrolled and randomised to treatment in this study are not permitted to be re-enrolled for a second course of treatment.

6.2 Inclusion criteria

The criteria for entry into the study are:

1. Diagnosis of rheumatoid arthritis made by a rheumatologist;
2. HLA-DRB1*0401+, HLA-DRB1*0101+ or HLA-DRB1*0401+HLA-DRB1*0101+ heterozygotes, homozygotes or compound heterozygotes; and ACPA+ (anti-CCP2>6);
3. Treatment with MTX at the same dose for at least 2 weeks prior to planned start of trial treatment;
4. Any swollen joint count at screening (0-28);
5. Age 18-75 years (male or female) inclusive;
6. Patients must be informed of the investigational nature of this study and give voluntary written informed consent in accordance with the institutional and hospital guidelines;
7. Male or Female. Females of child-bearing potential must agree to use two effective forms of contraception from enrolment to completion of the study;
8. Blood glucose, CBC, haemoglobin, platelets, creatinine, bilirubin, and AST/ALT not greater than 1.5 times out of normal range at entry and clinically insignificant in the opinion of the investigator;
9. Patients agree to forego vaccinations during the course of the study;

6.3 Exclusion criteria

The criteria for exclusion from the study are:

1. Malignancy;
2. An active inflammatory disease other than RA;
3. Currently receiving or have received treatment with >10mg prednisone daily within the last 2 weeks prior to screening;
4. Current or recent treatment (< 2 weeks prior) with any disease-modifying anti- rheumatic drugs other than methotrexate;
5. Serious infection requiring hospitalization within last 28 days;
6. Receipt of any live attenuated vaccines within 4 weeks prior to entry;
7. Major surgery within last 28 days;
8. Significant cardiovascular, renal, liver, neurological or skin disease;
9. Positive serology for HIV or infection with HBV or HCV;
10. Treatment with cytotoxic or immunomodulatory therapies such as radiotherapy, cyclophosphamide, mycophenolate, tacrolimus, PUVA, acitretin, cyclosporine or azathioprine;
11. Any known or suspected allergies to the study drug or its constituents including egg products;
12. Inadequate venous access to allow collection of blood samples;
13. History of drug or alcohol abuse;
14. Participation in another clinical study;
15. If, in the opinion of the PI, the subject appears not to be able to perform the needed responsibilities of participation in the clinical study.

6.3.1 Part B Specific Inclusion criteria

Diagnosis of rheumatoid arthritis made by a Rheumatologist within the previous 2 years

6.4 Other study eligibility criteria considerations

In order to assess any potential impact on subject eligibility with regard to safety, the Investigator must refer to the relevant document(s) for detailed information regarding warnings, precautions, contraindications, adverse events, and other significant data pertaining to the study product being used in the study. Such documents may include, but not limited to the Clinical Investigator’s Brochure (IB) or equivalent document provided by the Sponsor.

6.4.1 Contraception

All women of child bearing potential (defined as sexually mature women who have had menses within the preceding 24 months and have not undergone hysterectomy, bilateral oophorectomy or tubal ligation) must have a negative serum pregnancy test (with a sensitivity of at least 50 International Units/mL) performed at Screening and Baseline and prior to scheduled administration of investigational product.

Women of child bearing potential must agree not to attempt to become pregnant or undergo *in vitro* fertilisation and, if participating in sexual activity that could lead to pregnancy, must use two reliable methods of contraception simultaneously while receiving protocol-specified medication and for 28 days after stopping the medication. Male subjects must agree to use two reliable methods of contraception simultaneously while receiving protocol-specified medication and for 28 days after stopping the medication if their partner is of child bearing potential.

A combination of two of the following methods must be used:

- Condoms (male or female) with or without a spermicidal agent;
- Diaphragm or cervical cap with spermicide;
- Intra Uterine Device;
- Hormonal-based contraception.

Women who are not of child bearing potential are not required to use contraception.

6.4.2 Renal Function

Renal function eligibility may be determined by either estimated creatinine clearance calculated by Cockroft-Gault or serum creatinine, as clinically indicated.

The Cockcroft-Gault Formula to be used is:

$$\text{Creatinine clearance (mL/min)} = \frac{\text{(140-age) x (Bodyweight in kg) x (0.85 if female)}}{\text{(72 x Serum creatinine (mg/dL)}}$$

6.4.3 Rescreening

Subjects with laboratory abnormalities that are exclusionary, but inconsistent with previous history, may be re-tested twice (3 total tests) within a window of 56 days. Rescreening of these subjects must be discussed and agreed with the Sponsor or designee prior to enrolment in the study.

7 SCHEDULE OF ASSESSMENTS AND PROCEDURES

7.1 Study schedule of evaluations

The schedule of assessments is presented in Table 1.

7.2 Visit windows

Every effort should be made to keep to the study schedule.

Study Period	Acceptable Window
Screening	± 3 days
On-study (dosing)	± 2 days
Follow-up	± 7 days

The Sponsor or designee must approve all visits outside of these windows.

7.3 Study procedures / assessment period

The study procedures to be conducted for each subject enrolled in the study are listed below and in Section 7.4.

All laboratory tests on blood and urine samples will be performed at the designated local laboratory. Immunological assessments to support study objectives (non-exploratory endpoints) and measurements of calcitriol concentrations in plasma will be performed at central laboratories. Refer to the Study Reference Manual.

Additional visits and/or assessments may be conducted as clinically indicated.

7.3.1 Visit 1 Screening visit

Subjects will be screened within 35 days prior to randomisation to determine eligibility for participation in the study. The following will be performed and documented at Screening:

- Obtain written informed consent. (This must be completed prior to any other procedure)
- A screening medical history (see Section 7.4.1)
- A complete physical examination including: (see Section 7.4.1)
  - Vital signs
  - Bodyweight
  - Height
  - Assessment of all appropriate body systems to determine study eligibility
- Electrocardiogram (See section 7.4.2)
- Concurrent medication assessment (See Section 9)
- Blood samples for
  - Haematology including ESR (See section 7.4.3)
  - Clinical chemistry including CRP (See section 7.4.3)

  - Viral serology for HIV, HBV and HCV (See section 7.4.3)
  - Pregnancy testing (See Section 7.4.6)
- Blood samples for immunological assessments including:
  - HLA typing (if not already part of the participant's medical record; see Section 7.4.3)
  - Anti-CCP2 titre (See Section 7.4.3)
  - Rheumatoid Factor (See Section 7.4.3)
- Urine samples for urinalysis (see Section 7.4.2)
- DAS28CRPv4 evaluation (See section 7.4.7)

Subjects meeting the inclusion criteria and none of the exclusion criteria will return to the clinic within 35 days after screening for randomisation. If the patient misses randomisation and requires rescreening within a new 35 day window, blood samples for hematology and clinical chemistry including CRP, and pregnancy testing (if relevant) only are required to be repeated. The other laboratory and clinical data from the previous screen may be used, and updated with any relevant medical history/examination and medications.

7.3.2 Visit 2 (Day 1) baseline evaluations, randomisation, and study medication dispensing

At Visit 2 the following tests will be performed and documented on the Case Report Form (CRF) prior to administration of the IMP:

- Review of inclusion/exclusion criteria (see Section 6.2 and 6.3)
- Urine samples for urinalysis and pregnancy testing (see Section 7.4.3 and 7.4.6)
- A complete physical examination (see Section 7.4.1)
- Vital signs (see Section 7.4.1)
- Concurrent medication assessment (see Section 9)
- Adverse event questioning (see Section 10)
- DAS28CRPv4 evaluation (see section 7.4.7)
- Blood samples for:
  - Haematology including ESR (see Section 7.4.3)
  - Clinical chemistry including CRP (see Section 7.4.3)
- Blood samples for immunological assessments including (See sections 7.4.3 and 7.4.4):
  - Tetramer analysis (Collagen II and HA)
  - Exploratory Immune assays
- Blood samples for calcitriol concentration assessment (See Section 7.4.3)
- An oral swab will be (See Section 7.4.8)

Once the above assessments have been performed the patient will be randomised and the IMP will be administered.

- Subjects will be observed and vital signs will be measured at 15 and 30 minutes following the administration of IMP
- Subjects will remain in the clinic for at least four hours post-administration of IMP
- Blood samples for calcitriol concentration assessment will be taken 1, 2 and 4 hours after IMP administration

- The subject will be issued with an Injection Site Reaction Diary and instructed to view their injection site (s) at the same time each day and record their findings every day for 3 days.(See Section 7.4.5)

7.3.3 On-study clinic visits

7.3.3.1 Visit 3 (Day 8)

The subject will return to the clinic 7 days after administration of the IMP for visit 3. The following procedures will be performed:

- Symptom directed physical examination (See Section 7.4.1)
- Vital Signs (See Section 7.4.1)
- DAS28CRPv4 Evaluation (See Section 7.4.7)
- Review of Injection Site Reaction Diary (See Section 7.4.5)
- Blood samples for:
    - Haematology including ESR (See Section 7.4.3.)
    - Clinical chemistry including CRP (See Section7.4.3)
- Blood Samples for Immunological Assessment including (see Sections 7.4.3 and 7.4.4):
    - Tetramer analysis
    - Exploratory Immune assays;
- Blood Samples for Pharmacokinetic (Calcitriol) assessment (Part B only, See Section 7.4.3)
- Urine samples for urinalysis (See Section 7.4.3)
- Adverse event and concurrent medication questioning (see sections 9 and 10)
- Urine sample for pregnancy testing (See Section 7.4.6, part B only)

For subjects undergoing Part B of the study, once the above assessments have been performed the IMP will be administered:

- Subjects will be observed and vital signs will be measured at 15 and 30 minutes following the administration of IMP
- Subjects will remain in the clinic for at least four hours post-administration of IMP
- Blood samples for calcitriol concentration assessment will be taken 1, 2 and 4 hours after IMP administration
- The subject will be issued with an Injection Site Reaction Diary and instructed to view their injection site (s) at the same time each day and record their findings every day for 3 days (See Section 7.4.5).

7.3.3.2 Visit 4 (Day 15)

The subject will return to the clinic fourteen days following the first administration of the IMP for visit 4. The following procedures will be performed:

- Symptom directed physical examination (See Section 7.4.1)

- Vital Signs (See Section 7.4.1)
- DAS28CRPv4 Evaluation (see Section 7.4.7)
- For Subjects in Part B, review of Injection Site Reaction Diary (See Section 7.4.5)
- Blood samples for:
  - Haematology including ESR (See Section 7.4.3.)
  - Clinical chemistry including CRP (See Section 7.4.3)
- Blood Samples for Pharmacokinetic (Calcitriol) assessment (Part B only, See Section 7.4.3)
- Urine samples for urinalysis (See Section 7.4.3)
- Adverse event and concurrent medication questioning (see Sections 9 and 10)
- Urine sample for pregnancy testing (Part B only)

For subjects undergoing Part B of the study, once the above assessments have been performed the IMP will be administered:

- Subjects will be observed and vital signs will be measured at 15 and 30 minutes following the administration of IMP
- Subjects will remain in the clinic for at least four hours post-administration of IMP
- Blood samples for calcitriol concentration assessment will be taken 1, 2 and 4 hours after IMP administration
- The subject will be issued with an Injection Site Reaction Diary and instructed to view their injection site (s) at the same time each day and record their findings every day for 3 days (See Section 7.4.5).

7.3.3.3 Visit 5 (Day 29)

The subject will return to the clinic 21 days following the first administration of the IMP for visit 5. The following procedures will be performed:

- Symptom directed physical examination (See Section 7.4.1)
- Vital Signs (See Section 7.4.1)
- DAS28CRPv4 Evaluation (see Section 7.4.7)
- For Subjects in Part B, review of Injection Site Reaction Diary (See Section 7.4.5)
- Blood samples for:
  - Haematology including ESR (See Section 7.4.3.)
  - Clinical chemistry including CRP (See Section 7.4.3)
- Blood Samples for Immunological Assessment including (See Sections 7.4.3 and 7.4.4):
  - Tetramer analysis
  - Anti-CCP2 titre (See Section 7.4.3)
  - Exploratory immune assays
- Urine samples for urinalysis (See Section 7.4.3)
- Adverse event and concurrent medication questioning (see sections 9 and 10)

7.3.4 Visit 6 (Day 57) exit evaluation

The end of study visit (Visit 6) will be conducted in the clinic 56 days following the first administration of IMP. The following tests will be performed and documented as indicated on the CRF:

- Symptom directed physical examination (See Section 7.4.1)
- Vital Signs (See Section 7.4.1)
- DAS28CRPv4 Evaluation
- Blood samples for:
  - Haematology including ESR (See Section 7.4.3)
  - Clinical chemistry including CRP(See Section7.4.3)
- Urine samples for urinalysis and pregnancy testing (See Section7.4.3)
- An oral swab will be taken (See Section 7.4.8)
- Blood Samples for Immunological Assessment including (see Sections 7.4.3 and 7.4.4):
  - Anti-CCP2 (See Section 7.4.3)
- Adverse event and concurrent medication questioning (see sections 9 and 10)

7.4 Details of scheduled assessments

7.4.1 Demographic data, medical history, physical examination, vital signs

Demographic data will include sex, race/ethnicity and date of birth.

The medical history will include any diagnosed medical conditions or surgical history.

A complete physical examination (including head, ears, nose, throat, lungs, lymph nodes, heart, abdomen and skin) at screening will be conducted to determine study eligibility. A modified physical examination will also be performed at Days 1, 8, 15, 29 and 57 and at the early termination visit if applicable. The symptom directed physical examinations will be performed based on signs and symptoms the subject has reported verbally and/or documented in the diary booklets. A symptom directed physical examination may also be performed if in the opinion of the Investigator if it is required.

Vital signs measured:

- Body temperature (degrees Celsius) aural
- Respiratory rate
- Pulse rate (sitting)
- Blood pressure (sitting)

Height and body weight will be measured and documented at the Screening visit.

7.4.2 Electrocardiograms

The ECG recordings in this clinical trial will be performed in a standardised manner as outlined in the Study Reference Manual. Repeat measurements will be performed if there are any clinical abnormalities observed or artefacts are present. All ECG recordings will be reviewed by the Investigator or nominee.

The ECG recordings will be performed once the subject has been resting semi-supine for at least 10 minutes and will be measured in triplicate over approximately 3 minutes. The following parameters will be reported: QRS, QT, QTcB, RR and PR intervals.

The formula for calculating the corrected QT interval in respect to the heart rate will also be calculated using either Bazett or Fredericia formulas. If this isn't automatically calculated by the ECG machine, the following formulas will be used:

- Bazett's formula: QT / $(RR)^{0.5}$

  [Observed QT interval divided by root of RR interval, in seconds]
- Fridericia's formula: QT / $(RR)^{0.33}$

  [Observed QT interval divided by cube root of RR interval, in seconds]

Any clinically significant out of range or arrhythmia alarms will be printed out and recorded in the subject's CRF and reported as AEs if occurring after screening.

7.4.3 Blood and urine samples for laboratory tests

Blood and urine will be collected at all visits and should be within the visit window (see 7.2) Detailed instructions on the handling and processing of specimens are located in the Study Reference Manual. The following will be collected at the times specified in Section 7.3:

Haematology:	Haemoglobin, haematocrit, red blood cell (RBC) count , white blood cell and differential white blood cell count, platelet count, mean corpuscular volume (MCV), mean corpuscular haemoglobin concentration (MCHC), erythrocyte sedimentation rate (ESR).
Chemistry:	Sodium, potassium, chloride, bicarbonate, BUN, creatinine, total protein, albumin, serum amylase, phosphorus, AST, ALT, GGT, alkaline phosphatase, total bilirubin, glucose, CPK, calcium, uric acid, C-reactive protein (CRP)
Viral Serology:	HIV, HCV and HBV (at screening). HIV testing will be repeated after screening if there is a clinical suspicion that a subject may be at risk of infection.
Immunological:	HLA typing if required. HLA typing does not need to be repeated if part of medical record from an appropriately accredited laboratory, anti-CCP2 titre (if required), Rheumatoid Factor, Tetramer analysis (Collagen II peptide and other RA autoantigens), Exploratory immune assays (measures of humoral and T cell responses e.g. Interferon-gamma (IFN-γ) enzyme linked immunospot (ELIspot) assays, T-cell responses specific for RA autoantigens)
Urinalysis:	Protein, glucose, blood and microscopy (if abnormal).
Urine Pregnancy:	Human chorionic gonadotropin.
Serum pregnancy:	Human chorionic gonadotropin.
Pharmacokinetics:	Calcitriol concentration in plasma and pharmacokinetic indices

7.4.4 Handling and processing of biological specimens

All personnel involved in collecting and handling biological specimens should follow appropriate standardised operating procedures for handling biohazardous materials including those procedures recommended by the national regulatory authority.

Laboratory safety evaluations, including haematology, clinical chemistry, urinalysis and coagulation will be performed by the sites local laboratory.

Immunological testing will be performed by:

- Tetramer Analysis: Precision for Medicine
- HLA Typing: Victorian Red Cross
- Rheumatoid factor: Pathology Queensland
- Anti-CCP : Pathology Queensland
- ELISpot Analysis : Precision for Medicine
- Exploratory Immune Assays: Thomas Laboratory
- Microbiome analysis: Australian Centre for Ecogenomics at UQ

Pharmacokinetic analysis (plasma calcitriol concentration) will be performed by: Eurofins ADME BIOANALYSIS.

All contact details for laboratories are outlined in Section 2.0 of this protocol and in the Study Reference Manual.

7.4.5 Diary booklets

Subjects will be provided with a diary after each administration of DEN-181 to collect injection site reactions.

Injection Site Reaction Diary:

The aim of this diary is to collect information about injection site reactions for 3 days following the administration of IMP. Information that is to be collected includes the presence of:

- Pain/tenderness;
- Swelling/induration;
- Erythema; or
- Other findings.

Subjects will be provided with a ruler to enable the subject to measure and record the size of any injection site reactions that occur. Subjects will measure the longest diameter of any reaction area. Instructions on the measurement of reactions will be provided to the subjects within their diary booklet.

The Injection Site Reaction Diaries will be issued at visit 2 and collected at visit 3 for Parts A and B. In part B only, Diaries will also be issued at visit 3 (collected at visit 4) and visit 4 (collected at visit 5). The site staff will check that the diary is complete and legible while the patient is present after collection to ensure any clarifications can be performed.

7.4.6 Pregnancy tests

Serum pregnancy testing will be conducted for female subjects of childbearing potential at screening (visit 1). Before administration of the IMP (visit 2), visit 3 (Part B only), visit 4 (Part B only) and at the end of the study (visit 6) a urine pregnancy test will be performed.

If the urine pregnancy test is positive at visit 2, visit 3 (Part B only) or visit 4 (Part B only) a serum pregnancy test will be conducted to confirm the urine-based test and the IMP will not be administered until a negative result is obtained. If the serum pregnancy test is positive, the patient will be excluded or withdrawn from the study.

If the result of the urine pregnancy test is positive at visit 6, a serum pregnancy test will be conducted to confirm the urine-based test. If the serum-based test is positive at any visit, procedures for reporting a Clinical Trial Pregnancy will be performed.

7.4.7 DAS28CRPv4 evaluation

The DAS28CRPv4 is a frequent outcome measure used in rheumatoid arthritis therapeutic trials and is also used to guide treatment decisions. The DAS28CRPv4 related assessments and score calculation will be performed at all visits. See Appendix 2.

7.4.8 Microbiome Testing

Oral swabs will be taken at Baseline (visit 2) and at visit 6 so as to allow testing for the types of microbial communities found in these areas. Sequencing will be undertaken at Australian Centre for Ecogenomics at UQ.

8 INVESTIGATIONAL MEDICINAL PRODUCT

8.1 Randomisation process

This is a Phase I, randomised, double-blind, placebo-controlled, single-centre, single-dose escalation and multiple dose, dose-ranging parallel group study to investigate the safety, tolerability and pharmacodynamics of subcutaneously administered DEN-181 in adult patients with ACPA+ rheumatoid arthritis on stable treatment with methotrexate. The study is to be performed in two parts.

Part A is a single centre, randomised, double blind placebo controlled single-dose dose escalation study. Three cohorts will be dosed. Subjects in each cohort will be randomised to receive either DEN-181 or placebo in a 2:1 ratio of active to placebo with the respective groups having the following sample sizes:

- Group 1: 6 (4 active, 2 placebo)
- Group 2: 6 (4 active, 2 placebo)
- Group 3: 6 (4 active, 2 placebo)

A randomisation scheme will be prepared in advance by the study statistician using a computer generated system according to relevant SOPs. The scheme will have the property that within each dose cohort the initial sentinel cohort of 1 active and 1 placebo will be randomised prior to randomising the remaining subjects. That is:

- Group 1: Sentinel (1 active, 1 placebo) followed by 3 active and 1 placebo
- Group 2: Sentinel (1 active, 1 placebo) followed by 3 active and 1 placebo
- Group 3: Sentinel (1 active, 1 placebo) followed by 3 active and 1 placebo

Part B is a single-centre, randomised, double blind placebo controlled multiple-dose, dose-ranging parallel-group study. Two groups will be dosed. Subjects in each group will be randomised to receive either DEN-181 or placebo in a 3:1 ratio of active drug to placebo with the respective groups having the following sample sizes:

- Group 1: 20 (15 active, 5 placebo)
- Group 2: 20 (15 active, 5 placebo)

A randomisation scheme will be prepared in advance by the study statistician using a computer generated system according to relevant SOPs.

8.2 Blinding

Since DEN-181 is white and the placebo is clear, it will not be possible to blind the clinical staff who administer the dose. At the commencement of the study, the un-blinded staff who will administer IMP and back-up personnel will be identified and trained. Staff who administer IMP will not be permitted to be involved in patient assessments or management throughout the trial. Additionally they will not discuss what they believe the subject is receiving with either the subject or other staff members.

All other site staff and the subject will be blinded to the study allocation between DEN-181 and placebo. The Sponsor and its representatives will also be blinded to the treatment allocation throughout the course of the study. The investigational product administrators will make every attempt to ensure that both the subject and the blinded team members do not observe the subcutaneous injection as this may threaten the integrity of the blind. The subject may be asked to turn away during the injection. Similarly, other blinded site staff will be instructed to not observe the administration of the investigational product or placebo. This will be highlighted during the site initiation visit and throughout the study.

An un-blinded monitor will perform drug accountability according to the Monitoring Plan.

The dose level will not be blinded.

8.3 Treatment allocation

The pharmacist or responsible party will prepare the treatment corresponding to the subject number in the randomisation plan. A period of no less than 7 days will elapse between administration of IMP to the sentinel patients in Part A and the start of treatment for remaining patients of the same cohort.

8.4 Un-blinding

8.4.1 Medical emergency

Code break envelopes will be provided to the clinical trial site. The Investigator may request the treatment assignment in emergency situations only. The breaking of the blind will only be sanctioned where knowledge of the study medication treatment will affect patient management. The Investigator will discuss the need to un-blind with the Sponsor prior to any un-blinding taking place, unless immediate knowledge of the treatment allocated is required for patient care.

If the code is broken, a note to file will be generated by the individual who broke the code explaining the reason and date that the blind was broken. Details of the person who authorised the code break, and the identity of the study product allocated to the subject must be included in this note and countersigned by the Investigator.

Reason(s) for un-blinding will be clearly documented and the details included in the study report.

8.4.2 End of study

The randomisation code will be broken by the study statistician once data entry has been completed, the database locked, and the per-protocol population for analysis established and the Statistical Analysis Plan (SAP) finalised and approved. The Sponsor will provide written permission to the study statistician prior to the breaking of the randomisation code.

8.5 Formulation

8.5.1 Investigational medicinal products

DEN-181 is a sterile suspension of egg phosphatidylcholine (EPC) and egg phosphatidylglycerol (EPG) liposomes (approx. 100 nm diameter, lipid molar ratio 90:10) containing collagen II peptide (Pro273Hyp collagen $_{259\text{-}273}$) and calcitriol formulated in sucrose-buffered histidine buffer. Product is filled in 2 mL amber glass vials for single use. Each vial contains 1.0 mL extractable volume of DEN-181 containing 0.6 µg/mL calcitriol and 45µg/mL collagen II peptide. DEN-181 was manufactured by Evonik Canada Inc, (Burnaby, British Columbia, Canada) according to cGMP.

The placebo is sterile saline for injection (0.9%NaCl).

Product specifications and the associated manufacturing process are provided in the IB and a summary is provided below:

Table 8-1 Summary of DEN-181

**Name:**	**DEN-181**
Characteristics & Physical State:	Suspension of egg phosphatidylcholine (EPC): and egg phosphatidylglycerol (EPG) liposomes (approx. 100 nm diameter, lipid molar ratio 90:10) containing 0.6 µg/mL calcitriol and 45µg/mL collagen II peptide. The material will be provided frozen.
Supplied by:	Evonik Canada Inc. (Burnaby, British Columbia, Canada)
Storage Conditions:	Store at minus 20 degrees Celsius +/- 5 degrees
Package	2 mL amber vial

8.5.2 Supply, packaging and labelling, storage and handling

The batch number, expiry date and documented compliance with product release specification criteria will be included in the DEN-181 Certificate of Analysis. Evidence of cGMP will also be made available. The IMP will be supplied to the study site after receipt of required documents in accordance with the Sponsor authorised release procedures. Clinical trial labels will comply with Annex 13 of the Australian Code of Good Manufacturing Practice for Medicinal Products – Manufacture of Investigational Medicinal Products.

The following information will appear as secondary labelling i.e., on the label of the outermost container of the study investigational product vials:

- Dendright Pty Ltd, St. Lucia, QLD, Australia
- Protocol number: DEN-17-01-RA
- DEN-181 containing 0.6 µg/mL calcitriol and 45µg/mL collagen peptide
- Lot number
- Investigator Name
- Sterile aqueous suspension for subcutaneous injection
- Fill volume: 1.1 ml
- For Clinical Trial Use Only
- Store at: -20°C (±5°C)
- Date of manufacture
- Retest date.

The following information will appear as primary labelling i.e., on the label of the study investigational product vials:

- Dendright Pty Ltd.
- Protocol number: DEN-17-01-RA
- DEN-181 containing 0.6 µg/mL calcitriol and 45µg/mL collagen peptide
- Lot number
- Retest Date
- Fill volume: 1.1 ml

- Participant identification number
- Sterile aqueous suspension for subcutaneous injection.
- Store at: -20°C (±5°C)
- For Clinical Trial Use Only.

The packaging lot numbers will be recorded on the investigational product accountability record.

DEN-181 must be stored in a secure area with access limited to the pharmacist or responsible party and authorized staff. The vials and outer packaging will be stored frozen at -20 +/-5 ^{o}C, and thawed to room temperature (RT) prior to use. DEN-181 should be used within 4-6 hours of equilibration to RT. The DEN-181 vials contain 0.6 µg calcitriol and 45µg collagen peptide per 1.0 mL.

The Investigational Product must be prepared under aseptic conditions. Further details regarding preparation and administration of IMP are supplied in the Pharmacy Manual. The clinical study personnel must maintain a temperature log for the freezer which houses the investigational product. Daily minimum and maximum temperatures will be recorded in the temperature logs.

8.5.3 Dosage and administration of test drugs

For both Parts A and B, the different dosage volumes of IMP will be administered in the following way:

- Subjects will receive 100µl subcutaneous injections approximately 10 cm above the elbow joint overlying the deltoid muscle of the left upper arm. Where possible the same arm will be injected throughout the course of the study.
- For 300µl subcutaneous injections, 150µl will be delivered 10 cm above the elbow joint overlying the deltoid muscle of each upper arm.
- For 1000µl subcutaneous injections, 250µl will be delivered 10 cm above the elbow joint overlying the deltoid muscle of each upper arm and approximately 5 cm from the groin on each upper thigh.

The injection site will be cleaned prior to administration of the IMP by cleaning with an alcohol swab and air dried.

The participant must remain under supervision at the trial site for at least 4 hours after administration of the IMP. Vital signs including heart rate, supine pulse rate, temperature and supine blood pressure (SBP and DBP) and local reaction at the injection site will be assessed following IMP administration.

8.5.4 Dispensing and accountability

DEN-181 is supplied for use only in this clinical study and should not be used for any other purpose. The Investigator’s nominee will prepare the investigational product according to the procedure set out in the Pharmacy Manual.

The Investigator will be responsible for maintaining accurate records for all study medications dispensed and returned. The inventory must be available for inspection by the study monitor. Study product supplies, including partially used or empty (describe unit/container e.g. syringe, tablets, vials, bottle, pack) and the dispensing logs, must be accounted for by the study monitor and returned to the drug repository for destruction at the end of the study.

When requested in writing by the Sponsor, unused study medication supplies may be destroyed by the Investigator provided such disposition can be performed safely. Records shall be maintained by the Investigator or any such alternate disposition of the study medication. These records must show the identification and quantity of each unit disposed of, the method of destruction (taking into account the requirements of local law), and the person who disposed of the test substance. Such records shall be submitted to the sponsor.

9 CONCURRENT MEDICATIONS AND TREATMENTS

At each study visit, the Investigator should question the subject about any medication taken including vitamin supplements and herbal remedies. Any concurrent medications will be recorded in the subject's records and the CRF. Any changes in doses or introduction of a new medication during the course of the study will also be recorded.

9.1 Special dietary requirements

There are no special dietary requirements.

9.2 Concurrent medications/treatments not permitted

9.2.1 Prior to study entry

Refer to exclusion criteria in Section 6.3 with particular note to the following medications and treatments that are not permitted:

- Currently receiving >10mg prednisone/day;
- Receipt of any live attenuated vaccines within 4 weeks prior to entry;
- Treatment with cytotoxic or immunomodulatory therapies such as radiotherapy, cyclophosphamide, mycophenolate, tacrolimus, PUVA, acitretin, cyclosporine or azathioprine;
- Concurrent treatment or treatment within 2 weeks of trial commencement with any DMARD other than MTX;
- Other investigational drugs.

9.2.2 During the study dosing period

Throughout the study medication dosing period, subjects may not receive any of the following concomitant medications:

- Immunomodulating agents (including immunosuppressive agents, interferon or other immune or cytokine-based therapies),and/or systemic chemotherapeutics;
- Chronic treatment with immune-suppressive therapy (stable use of asthma inhalers and topical corticosteroids are permitted);
- Subjects also should not receive any other vaccines during their participation in this study;
- Other investigational drugs.

NSAID and steroid therapy (e.g. intra-articular joint injection or daily oral prednisone at> 10 mgs/day) may be utilised, if required, after Day 29 of the study. Subjects can receive prednisone at < 10 mgs/day either stably or intermittently during the study.

All concurrent medications, including vitamin supplements and herbal remedies, must be recorded in the appropriate section of the CRF.

10 ADVERSE EVENTS AND TOXICITY MANAGMENT

10.1 Safety parameters

Safety parameters will include adverse events, vital signs, RA disease severity and clinical laboratory tests.

10.2 Adverse events

An adverse event or adverse experience (AE) is any untoward medical occurrence in a subject or clinical investigation subject administered an investigational product (whether it is the experimental product of the control) and which does not necessarily have a causal relationship with the Investigational Product. An AE can therefore be any unfavourable and unintended sign, symptom, or disease temporally associated with the use of a medicinal product, whether or not considered related to the medicinal product. Pre-existing events, which increase in frequency or severity or change in nature during or as a consequence of use of a drug in human clinical trials, will also be considered as adverse experiences. AEs may also include pre- or post-treatment complications that occur as a result of protocol-mandated procedures (e.g. invasive procedures such as biopsies).

Any AE (i.e. a new event or an exacerbation of a pre-existing condition) with an onset date after IMP administration up to the last day on study (including the follow-up, off study medication period of the study), should be recorded as an AE on the appropriate CRF page(s).

An AE **does not** include:

- Medical or surgical procedures (e.g. surgery, endoscopy, tooth extraction, transfusion); the condition that leads to the procedure is an adverse event;
- Pre-existing diseases or conditions present or detected prior to start of study product administration, that do not worsen;
- Situations where an untoward medical occurrence has not occurred (e.g. hospitalisation for elective surgery, social and/or convenience admissions);
- Overdose of either study product or concomitant medication without any signs or symptoms unless the subject is hospitalised for observation.

10.2.1 Assessment of AEs

All AEs will be assessed by the Investigator and recorded on the appropriate CRF page, including the date of onset and resolution, severity, relationship to study product, outcome and action taken with study medication. See Appendix 1 for details of the NCI CTCAEv4.03 toxicity grading scale.

In addition, injection site reactions will be graded by an Investigator or designee, according to severity. It is anticipated that the subjects may find it difficult to differentiate pain and tenderness and for this reason pain and tenderness have been combined in the following grading scale:

Table 7 – Grading for Pain and Tenderness at injection sites

**Mild**<br><br>**Grade 1**	**Moderate**<br><br>**Grade 2**	**Severe**<br><br>**Grade 3**
Awareness of symptoms but easily tolerated	Discomfort enough to cause interference with usual activities	inability to do work or usual activities

For all other reported adverse events, severity should be recorded and graded as:

**Grade**	**Severity**	**Comments**
1	Mild	Aware of sign or symptom, but easily tolerated. Concomitant medication is not ordinarily indicated for relief of mild AEs
2	Moderate	Discomfort enough to cause interference with usual activities. Concomitant medication may be indicated for relief of moderate AEs
3	Severe	Incapacitating with inability to work or perform usual activities. Concomitant medication may be indicated for relief of severe AEs

The relationship to study drug should be assessed using the following definitions:

**Causality**	**Comment**
Unrelated	AE is clearly due to extraneous causes (e.g. underlying disease, environment, known effect of another drug)
Unlikely	The temporal association between the AE and study drug is such that study drug is not likely to have any reasonable association with the AE
Possible	The AE could have been produced by the subject's clinical state or study drug
Probable	The AE follows a reasonable temporal sequence from the time of study drug administration, abates upon discontinuation of the study drug and cannot be reasonably explained by the known characteristics of the subject's clinical state
Definite	The AE follows a reasonable temporal sequence from the time of study drug administration, abates upon discontinuation of the study drug and/or reappears when study drug is re-introduced

These criteria in addition to good clinical judgment should be used as a guide for determining the causal assessment. If it is felt that the event is not related to study product, then an alternative explanation should be provided.

10.2.2 Adverse event reporting period

All adverse events, regardless of severity, causality or seriousness must be reported from the date of informed consent until the end of the study or 28 days after the last dose of study medication, whichever is later. However, any adverse event that the investigator believes is at least possibly related to study medication should be reported regardless of time elapsed from the final dose.

10.3 Serious adverse events

10.3.1 Serious adverse event definition

A **serious adverse event** (SAE) is defined as follows:

Any adverse drug experience occurring at any dose that results in any of the following outcomes:

- death
- life-threatening situation (subject is at immediate risk of death)
- inpatient hospitalisation or prolongation of existing hospitalisation (excluding those for study therapy or placement of an indwelling catheter, unless associated with other serious events)
- persistent or significant disability/incapacity
- congenital anomaly/birth defect in the offspring of a subject who received study product
- Other: Important medical events that may not result in death, be immediately life-threatening, or require hospitalisation, may be considered a SAE when, based upon appropriate medical judgment, they may jeopardise the subject and may require medical or surgical intervention to prevent one of the outcomes listed in this definition. Examples of such events are:
    - intensive treatment in an emergency room or at home for allergic bronchospasm
    - blood dyscrasias or convulsions that do not result in hospitalisation
    - development of drug dependency or drug abuse

10.3.2 Clarification of serious adverse events

Death is an outcome of an AE, and not an AE in itself. In reports of death due to “Disease Progression”, where no other information is provided, the death will be assumed to have resulted from progression of the disease being treated with the study product(s).

All deaths, regardless of cause, must be reported to Sponsor for subjects on study and for deaths occurring within 30 days of dosing of last study drug or within 30 days of last study evaluation, whichever is longer.

“Occurring at any dose” does not imply that the subject is receiving study drug at the time of the event. Dosing may have been given as treatment cycles or interrupted temporarily prior to the onset of the SAE, but may have contributed to the event.

“Life-threatening” means that the subject was at immediate risk of death from the event as it occurred. This does not include an event that might have led to death, if it had occurred with greater severity.

Complications that occur during hospitalisations are AEs. If a complication prolongs hospitalisation, it is a SAE.

“In-patient hospitalisation” means the subject has been formally admitted to a hospital for medical reasons, for any length of time. This may or may not be overnight. It does not include presentation and care within an emergency department.

The Investigator should attempt to establish a diagnosis of the event based on signs, symptoms and/or other clinical information. In such cases, the diagnosis should be documented as the AE and/or SAE and not the individual signs/symptoms.

10.3.3 Serious adverse event reporting requirements

10.3.3.1 All SAEs

The Sponsor has responsibilities for expedited reporting of SAE's meeting specific requirements to worldwide regulatory authorities; therefore, all appropriate parties must be notified immediately regarding the occurrence of any SAE that occurs after the first dose of study product or placebo has been administered. The procedures for reporting all SAEs, regardless of causal relationship, are as follows:

- Complete the "Serious Adverse Event Report" CRF page
    - Email the SAE report to the CRO SAE email address within 24 hours of the Investigator's knowledge of the event at the numbers below [Please refer to the Study Reference Manual for the designated email address]
    - for fatal or life-threatening events, also fax copies of hospital case reports, autopsy reports, and other documents when requested and applicable

The Sponsor may request additional information from the Investigator to ensure the timely completion of accurate safety reports.

The Investigator must take all therapeutic measures necessary for resolution of the SAE. Any medications necessary for treatment of the SAE must be recorded onto the concurrent medication section of the subject's CRF.

10.3.3.2 Investigator reporting requirements for SAEs

An SAE may qualify for reporting to regulatory authorities if the SAE is considered to have a possible causal relationship to the study product, and is unexpected (Suspected Unexpected Serious Adverse Reaction [SUSAR]) based upon the current Investigator's Brochure. In this case for multi-centre studies, all Investigators will receive a formal notification describing the SAE.

Where this is required by local regulatory authorities, and in accordance with the local institutional policy, the Investigator should notify (in writing) the Human Research Ethics Committee (HREC) which approved the study of the SAEs, according to HREC requirements as soon as is practical.

10.4 Follow up of serious and non-serious adverse events

Follow-up of serious and non-serious AEs will continue through the last day on study (including the follow-up, off study medication period of the study), until the Investigator and/or the Sponsor determine that the subject's condition is stable, or up to 30 days after the last dose of Study Product, whichever is longer. The Sponsor may request that certain AEs be followed until resolution.

10.5 Clinical Laboratory Abnormalities and Other Abnormal Assessments as AEs or SAEs

All laboratory values must be reviewed in real time by the Investigator. Given that all laboratory data are collected and statistically analysed according to their respective toxicity gradings, laboratory abnormalities that occur without related clinical symptoms and signs should generally not be recorded as AEs unless they represent a clinically significant event. Where possible, the overall diagnosis rather than the laboratory abnormality should be recorded on the AE CRF. This will avoid duplication of laboratory abnormalities in both the AE and laboratory reports. Abnormal laboratory results that are of clinical significance should be reviewed by the Medical Monitor.

Any laboratory test result that meets the criteria for a SAE (refer to Section 10.3) should be recorded as an AE, the AE page of the CRF completed and a SAE form also completed in order for the sponsor to collect additional information about that abnormality, including information regarding relationship to study product or other causes, any action taken and resolution.

10.6 Toxicity Management

AEs and abnormal laboratory values, if appropriate, should be graded according to the NCI CTCAE v4.03 grading scale (Appendix 1). Where parameters are not addressed within the *TGS,* laboratory abnormalities should be assessed as “Clinically Significant” or “Not Clinically Significant”. All abnormal values should be confirmed by repeat testing as soon as possible, preferably within 3 calendar days of receipt of results prior to dose interruption or discontinuation, unless such a delay is not consistent with good medical practice.

For the purpose of monitoring toxicities, the baseline value is defined as the last value prior to the administration of the first dose of study product. This value must be obtained from the local laboratory. All management is based on changes from this value.

All results that are classified as “Clinically Significant” should be documented in the AE form of the CRF.

10.7 Guidance for dose modification or discontinuation of treatment

To date, no specific toxicities have been identified that result from DEN-181 treatment. Any toxicities and/or abnormal laboratory findings should be investigated for aetiology and graded according to Appendix 1.

The following toxicity management will be followed:

- Grade 1 or 2: Patients may continue study treatment/placebo (except if they have an elevated temperature of >38 degrees Celsius or inter-current systemic illness). Dosing may be delayed for up to 7 days. Any dosing delays of >7 days may require that the subject is withdrawn after discussion with the Medical Monitor. Safety assessments will occur immediately prior to each dosing of IMP;
- Grade 3: Patients with any Grade 3 toxicity considered to be at least possibly related to treatment (i.e. treatment related events) should be evaluated carefully by the Investigator prior to continuing IMP treatment. Investigators may discuss individual cases with the Medical Monitor;
- Grade 4: Patients developing any Grade 4 toxicity should have treatment interrupted. The Investigator should contact the Medical Monitor to discuss the subject’s withdrawal.

Subjects will be withdrawn from treatment permanently should any of the following occur:

- Treatment related Grade 4 toxicity
- The need to take medication which could interfere with study measurements
- Subject unwilling to proceed and/or consent is withdrawn.

Where possible subjects who are withdrawn from treatment will remain on the study and attend the scheduled study visits, provided the Investigator deems it appropriate and the Sponsor approves.

Dosing will be stopped if suspected adverse drug reactions, changes to vital signs or clinical laboratory results are observed and these changes pose a significant health risk.

A corresponding adverse event will be captured within the CRF.

For any subject experiencing any event (irrespective of severity) which, in the opinion of the Investigator, contraindicates further dosing in that subject and the event is considered to be at least possibly related to treatment (or where causality to study treatment cannot be ruled out – see Section 10.2.1), continued dosing of the subject should be interrupted. In all cases, the final treatment decisions, made in response to toxicity, are the responsibility of the Investigator. A careful evaluation of the potential risk/benefit will dictate the optimal therapeutic course. Should treatment be interrupted, re-initiation will follow review of the available safety data and in agreement with the Sponsor. There will be no dose reduction or modification.

If the Investigator deems that management of the patient's medical condition requires knowledge of the study treatment regimen, then the Medical Monitor must be contacted for consultation and the procedures described in Section 7.3 followed. If a patient dies from an event that is considered to be at least possibly related to treatment, continued dosing of all study participants should be interrupted until the SMC has reviewed the data.

Clinically significant suspected adverse drug reactions, and serious adverse events considered to be related to study procedures will be followed until resolved or considered stable. All subjects who experience a study drug related AE should be followed until resolution of the AE, even if the subject has discontinued study drug.

If an unscheduled interruption of study drug occurs, the study site should notify the Sponsor at the earliest possible time. In the event that a subject requires an unscheduled interruption of study drug under conditions other than those associated with toxicity, the case will be reviewed by the Sponsor to determine whether such a subject will be allowed to resume study drug.

Subjects withdrawn from study drug will be treated as deemed appropriate by the Investigator. Follow- up procedures should be performed and the appropriate CRFs should be completed.

The study may be terminated at any point in time at the discretion of the Sponsor.

10.8 Warnings and precautions

This is a first in man study of DEN-181 so the adverse event profile is not characterised and cannot be reliably predicted. The preclinical data suggest an acceptable safety profile and a safety margin in excess of 10x is associated with the highest dose selected for study. Facilities and staff for resuscitation and treatment of other medical emergencies will be provided if required

10.9 Risks for women of childbearing potential or during pregnancy

The risks of treatment with Study Product during pregnancy have not been evaluated. Pre-menopausal women of childbearing potential will follow a medically prescribed birth control regimen or agree to abstain from heterosexual intercourse while participating in the study and for 30 days following the last dose of Study Product.

10.10 Procedures to be followed in the event of pregnancy

The subject must be instructed to inform the Investigator immediately if she becomes pregnant during the study or in the case of male subjects, their partners become pregnant during the study. The Investigator should report all pregnancies to the CRO within 24 hours of becoming aware of the pregnancy. Pregnancies should be reported using the form for reporting the occurrence and outcome of pregnancies in subject enrolled in the study.

Monitoring of the subject should continue until conclusion of the pregnancy. The outcome of the pregnancy should be reported to the CRO if the study is still in progress at the end of the pregnancy. If the end of the pregnancy occurs after the study has been completed, the outcome should be reported to Sponsor.

11 DATA SAFETY MONITORING

The Sponsor has convened a Safety Monitoring Committee charged with the review of safety data and dosing decisions during the conduct of the study.

The SMC will be responsible for reviewing safety data in Part A so as to allow dose escalation and enrolment of the next dose level. Dose escalation will be determined after each cohort has been enrolled and data are available through Day 15.

The SMC will also be responsible for a full review of all available safety data upon administration of Investigational Product to all subjects so as to assess whether it is safe to initiate Part B of the study. This safety review will be initiated upon receipt of safety data minimally up to at least the Day 15 visit for all study participants in Part A. At this time, all available data will be reviewed by the SMC and agreement to proceed to Part B formalised in writing.

Ad-hoc meetings will be scheduled immediately should any safety concerns arise at any time during the conduct of the study.

The treatment blind will not be broken for the purposes of SMC review, and the SMC will review only data blinded to treatment allocation.

Meetings will be convened to review aggregate and individual patient level data by injection regimen prepared as listings and tables. At a minimum, baseline patient characteristics, vital signs and adverse events will be provided.

Membership of the SMC will include the Investigator(s), Medical Monitor for the study and one company representative. An expert advisor on rheumatoid arthritis and/or clinical immunology will be invited, as appropriate. The Clinical Project Manager or other Sponsor designated staff will be invited to facilitate information dissemination and preparation of documentation of meeting outcomes and decisions.

Details of the membership function and decision making processes of the SMC for this study are set out in a separate SMC Charter.

12 SUBJECT COMPLETION/WITHDRAWAL

12.1 Subject completion

A subject will be deemed to have completed the study once all trial procedures have been conducted. Any AEs or SAEs still ongoing at the time of the exit evaluation will be followed in accordance with Section 10.

12.2 Criteria for premature withdrawal from treatment or the study

Subjects have the right to withdraw from treatment or the study at any time for any reason. The Investigator must make every reasonable effort to keep each subject in the study except where termination or withdrawal is for reasons of safety. The Investigator also has the right to withdraw patients from treatment or the study in the event of concurrent illness, AEs, pregnancy, treatment failure after a prescribed procedure, protocol violations, administrative reasons or other reasons.

It is understood by all concerned that an excessive rate of withdrawals from the study can render the study difficult to interpret; therefore, unnecessary withdrawal of subjects from the study should be avoided.

The reasons for withdrawal of the subject must be recorded on the CRF. The following are considered justifiable reasons for subject withdrawal:

- The need to take medication which may interfere with study measurements
- Intolerable/unacceptable adverse experiences
- Major violation or deviation of study protocol
- Non-compliance of subject with protocol
- Subject unwilling to proceed and/or consent is withdrawn
- Withdrawal from the study is, in the Investigator’s judgement, in the subject’s best interest
- Pregnancy of female study subject at any time during the study period (if applicable).

12.3 Withdrawal of subjects from study product

Investigational medicinal product is administered once during Part A of this protocol and three times during the conduct of Part B of this protocol.

Section 10.7 provides guidance for dose modification or discontinuation of IMP in the event of AEs or abnormal laboratory values.

If a subject permanently discontinues dosing with IMP, for example as a result of an AE, every attempt should be made to keep the subject in the study and continue to perform the required study related follow-up and procedures.

The ideal is to continue to follow the subject for the full study period or until resolution of the adverse event. If it is not possible for the subject to remain in the study for the full study period, every attempt should be made to keep the subject in the study up to the Exit Evaluation at Day 57. If the subject remains in the study but off study treatment, all study procedures should continue as per protocol.

If the subject will not remain in the study for all study related procedures, the Exit Evaluation should be performed wherever possible.

All subjects who discontinue study medication dosing should be followed for at least 30 days after the last dose of IMP in order to monitor subjects for possible post-treatment events which may occur after IMP has been discontinued.

12.4 Withdrawal of subjects from the study

Should a subject decide to withdraw from the study, all efforts will be made to complete and report the observations as thoroughly as possible.

The Investigator should contact the subject either by telephone or through a personal visit, or a responsible relative must be contacted to determine, if possible, the reason for withdrawal. A complete final evaluation at the time of the subject withdrawal should be made with an explanation of why the subject is withdrawing from the study.

If the reason for removal of a subject from the study is an AE or an abnormal laboratory test result, the principal reason will also be recorded on the CRF. Where possible, subjects should be followed until the AE is resolved or the abnormal laboratory test has returned to normal.

12.5 Replacement of withdrawn subjects

Any subjects who discontinues in a clinical study of their own volition or by the Investigator are defined as “withdrawals”. There will be no replacement of subjects withdrawing from the study.

12.6 Premature termination of the study

The Sponsor reserves the right to terminate the study at any time. Reasons will be provided in the event of this happening.

13 STATISTICAL ANALYSIS

13.1 Hypothesis

Given this is a first in human study with the focus on safety and proof of concept testing, the focus of the study will be descriptive rather than hypothesis testing. If inferential statistical tests of hypotheses are employed, they will be viewed as exploratory and descriptive rather than confirmatory.

13.2 Sample size determination

No formal sample size calculations were performed for this study.

13.3 Randomisation

This is a randomised, double-blind, placebo-controlled, single-centre, single-dose escalation and multiple-dose, dose-ranging parallel group study to investigate the safety, tolerability, and pharmacodynamics of subcutaneously administered DEN-181 in adult patients with ACPA+ rheumatoid arthritis on stable treatment with methotrexate (MTX). It is to be performed in two parts:

Part A is a randomised, double-blind, placebo controlled single-dose escalation study. Within each dosing cohort, subjects will be randomized to receive DEN-181 or placebo in a 2:1 allocation ratio. A sentinel dose will be administered to 2 patients who will be randomized to receive study drug or placebo.

Part B is a randomised double blind, placebo-controlled single centre multiple-dose, dose-ranging parallel group study at two dose levels. Within each group subjects will be randomized to receive DEN-181 or placebo in a 3:1 allocation ratio.

The randomisation process in respect of DEN-181 or placebo will be double blinded in both parts of the study.

13.4 Criteria for evaluation of study objectives

13.4.1 Definition of evaluation of safety

The primary objective of this study is to evaluate the safety and tolerability of single and multiple doses of DEN-181 in adult patients with ACPA+ RA on stable treatment with methotrexate. The primary endpoints for evaluating safety and tolerability are changes in vital signs, adverse events, laboratory abnormalities and withdrawal from the study. Changes in RA disease severity will also be evaluated using the DAS28CRPv4 scoring system.

13.4.2 Definition of evaluation of secondary study objective(s)

The secondary objectives of this study are to:

- To determine the ability of DEN-181 to modulate the total T-naïve, T-effector and T-regulatory cell count in peripheral blood;
- To determine the antigen-specific immune response to DEN-181 including the determination of collagen II peptide specific T-naïve, T-effector and T-regulatory cells;
- To determine preliminary clinical efficacy of DEN-181 in MTX-treated early ACPA+ RA patients;
- Monitor plasma concentrations of calcitriol.

13.4.3 Analysis populations

Per Protocol Analysis Set: The per-protocol analysis set will consist of all randomized subjects receiving study drug who are compliant with inclusion/exclusion criteria, study drug administration, study procedures, and measurement follow-up. Further details including the criteria for compliance will be detailed in the SAP.

Intent-to-Treat Set (ITT): The ITT analysis set will include all randomized subjects. Subjects will be analysed according to their randomized treatment group regardless of exposure to study drug or the actual treatment received.

Safety Analysis Set: The safety analysis set will include all subjects who receive at least one injection of study drug. Subjects will be included in the treatment group according to the actual treatment received regardless of their randomized assignment.

13.4.3.1 Group comparability

Demographic and baseline information including disease status and medical conditions, will be summarised and tabulated by treatment group. The purpose of these summaries is (1) to characterise the study population, and (2) to identify any baseline imbalances of clinical significance that may require consideration in the evaluation of efficacy and safety.

13.4.3.2 Data analysis methods

The intent-to-treat population will be used for analyses of immune and biomarker responses to DEN-181, and the safety population will be used for safety analyses.

Details of the analysis methods will be included in the SAP.

13.5 Statistical and analytical plan

A detailed SAP will be prepared prior to inspection of any data by the biostatistician and prior to database lock.

The SAP will include details of the following:

- Definitions of analysis populations
- Description of all data transformations and data derivations to be used together with rationale and references
- Details of hypotheses to be tested (if any), together with treatment effects and corresponding confidence intervals to be estimated
- Details of methods for checking the appropriateness of the chosen statistical model
- Discussion of the use of baseline data to improve precision or to adjust estimates for potential baseline differences (e.g. analysis of covariance)
- Discussion of multiplicity and adjustment procedures if required
- Methods of dealing with missing values and outliers together with details of any sensitivity analyses to be performed
- Specification of any subgroup or interaction analyses

- Details of any planned interim analyses
- Example table and listing shells to indicate the proposed presentation of the data.

13.6 Final analyses

At the completion of the study a final clinical/statistical study report will be prepared.

13.6.1 Analysis of demographics

Descriptive statistics with regard to demographic and other baseline characteristics will be described by assigned group using the ITT analysis set. Tables of descriptive statistics for subject demographics and baseline characteristics will also be provided.

13.6.2 Analysis of Immunomodulation

The primary variables associated with immunomodulation are the proportion and phenotype of effector and regulatory T-cells (i.e. total and antigen-specific) measured by flow cytometry and MHC II tetramer analysis.

The primary endpoint associated with immunomodulation will be mean change in proportion and phenotype of effector and regulatory T-cells (i.e. total and antigen-specific) measured by flow cytometry and MHC II tetramer analysis before and after administration of DEN-181.

13.6.3 Analysis of safety

All subjects receiving study medication will be included in the safety analyses.

13.6.4 Adverse events

The primary analysis of adverse events will consider only treatment-emergent AEs (TEAEs), events occurring for the first time, or worsening, during or after the first administration of study drug. The analysis will focus on subject incidence, although for TEAEs of special interest, the number of events may also be provided. Tables summarizing TEAEs will be displayed by preferred term and system organ class (SOC). The analysis will also include the analysis of serious adverse events (SAEs) and the categorization of AEs by severity and relationship to study drug. The analysis of AEs leading to study withdrawal or deaths on-study will also be provided in tables and/or listings. Further details will be provided in the SAP.

13.6.5 Vital signs, laboratory evaluations and DAS28CRPv4

The analysis of vital signs and laboratory parameters will include tabulations by dose/injection regimen. Descriptive statistics for each parameter and/or the change from baseline at each time point will be provided. For endpoints measured on a categorical scale the number of subjects and percentages will be provided. Further details will be provided in the SAP.

13.6.6 Other exploratory biomarkers and analysis of immunomodulatory effects

Various exploratory immune assays will be performed as measures of humoral and T-cell responses.

These may include, but are not limited to:

- Proportion and phenotype of naïve, effector and regulatory T cells for other autoantigens (e.g. cit-vimentin)

- T-cell responses as determined by interferon-gamma (IFN-γ) enzyme-linked immunospot (ELIspot) assays;
- CD4 T-cell responses as measured by intracellular cytokine staining (ICS) (e.g., IL-2 and IFN-γ).
- Antibody titres to RA autoantigens including collagen, modified collagen and ACPA

Various exploratory biomarker readouts will be performed including measurements of cytokine levels.

The exploratory biomarker endpoints will be mean change from baseline using assay read-outs measured before and after administration of DEN-181.

13.6.7 Analysis of calcitriol levels in plasma

The level of calcitriol in the plasma will be measured pre-administration of IMP and one, two and 4 hours post-administration on Day 1 at Baseline (Parts A and B) and at Days 8 and 15 (Part B only). Calcitriol levels pre- and post-administration of DEN-181 will be tabulated and reported using standard pharmacokinetic indices.

13.7 Interim analysis

No interim analysis is planned

13.8 Analysis of Part A Data and Dose selection for Part B

Part B of the study utilises two different dose levels of DEN-181, these two dose levels to be selected from the three dose levels utilised in Part A based upon analysis of the Part A Safety Data (see 13.6.3, 13.6.4 and 13.6.5) and analysis of immunomodulation data (see 13.6.2). The SMC will also be responsible for this review of all available safety data upon administration of Investigational Medicinal Product to all subjects in Part A so as to assess whether it is safe to initiate Part B of the study and to select doses to be utilised in Part B. This safety review will be initiated upon receipt of safety data minimally up to at least the Day 15 visit for all study participants in Part A.

14 GENERAL STUDY ADMINISTRATION

14.1 Ethical aspects

14.1.1 Local regulations/declaration of Helsinki

The Investigator will ensure that this study is conducted in full conformance with the protocol, the latest version of the “Declaration of Helsinki (and its amendments and with the requirements of national drug and data protection laws of the countries in which the research is conducted).

In other countries, the Sponsor and the Investigators will ensure strict adherence to the provisions of Good Clinical Practice and all applicable and national regulations. The International Council on Harmonisation (ICH) guidelines will apply.

14.1.2 Informed consent

It is the responsibility of the Investigator to obtain written informed consent from each individual participating in this study after adequate explanation of the aims, methods, objectives and potential hazards of the study prior to undertaking any study related procedures. The Investigator must also explain to the subject that they are completely free to refuse to enter the study or to withdraw from it at any time for any reason. The Investigator must utilise an HREC approved consent form for documenting written informed consent.

14.1.3 Premature withdrawal

If subjects are unable to receive the study medication between enrolment and dosing, every attempt should be made to keep the subject in the study and continue to perform the required study related procedures. If this is not possible or acceptable to the subject or Investigator, the subject may be withdrawn from the study. Refer to Section 12 for more detail.

14.1.4 Institutional review boards or ethics committees

This protocol and any accompanying material provided to the subject (such as subject information sheets or descriptions of the study used to obtain informed consent), will be submitted, by the Investigator, to an HREC. Approval from the committee must be obtained before starting the study, and should be documented in a letter to the Investigator specifying the protocol number and version and the date on which the committee met and granted the approval.

Any modifications made to the protocol after receipt of HREC approval must also be submitted by the Investigator to the committee in accordance with institutional procedures and regulatory requirements.

14.1.5 Conditions for modifying the protocol

Protocol modifications to ongoing studies which could potentially adversely affect the safety of participating subjects or which alter the scope of the investigation, the scientific quality of the study, the experimental design, dosages, duration of therapy, assessment variables, the number of subjects treated or subject selection criteria, may be made only after consultation between an appropriate representative of the Sponsor and the Investigator.

Protocol modifications (amendments) must be prepared by a representative of the Sponsor and initially reviewed and approved by the responsible Medical Monitor and (when applicable) the Statistician.

All protocol modifications must be submitted to the HREC in accordance with local requirements. Approval must be obtained before changes can be implemented.

In the event of an emergency, the Investigator may institute any medical procedures deemed appropriate. However, all such procedures must be promptly reported to the Sponsor, the Medical Monitor and the HREC.

Administrative changes of the protocol are defined as minor corrections and/or clarifications that have no effect on the way the study is to be conducted, or on the safety of the subjects. These administrative changes will be agreed upon by the Sponsor and the Investigator, and will be documented in a memorandum. The Investigator will then notify the IEC/IRB of such administrative changes as appropriate.

14.1.6 Conditions for terminating the study

Both the Sponsor and the Investigator reserve the right to terminate the study at any time. Should this be necessary, the procedures will be arranged on an individual study basis after review and consultation by both parties. In terminating the study, the Sponsor and the Investigator will assure that adequate consideration is given to the protection of the subject's interests.

14.2 Study documentation, CRFs and record keeping

14.2.1 Investigator's files/retention of documents

The Investigator must maintain adequate and accurate records to enable the conduct of the study to be fully documented and the study data to be subsequently verified. These documents should be classified into two separate categories: (1) Investigator's Site File, and (2) subject clinical source documents.

The Investigator's Study File will contain the protocol/amendments, Case Report and Query Forms, HREC and governmental approval with correspondence, informed consent, drug records, staff curriculum vitae and authorisation forms and other appropriate documents and correspondence.

Subject clinical source documents (usually defined by the project in advance to record key efficacy/safety parameters independent of the CRFs) would include subject hospital/clinic records, physician's and nurse's notes, appointment book, original laboratory reports, ECG, EEG, X-ray, pathology and special assessment reports, consultant letters, screening and enrolment log, etc. All clinical study documents must be retained by the Investigator until at least 2 years after the last approval of a marketing application in an International Council on Harmonization (ICH) region (i.e. USA, Europe, or Japan) and until there are no pending or contemplated marketing applications in an ICH region; or if no application is filed or if the application is not approved for such indication, until 2 years after the investigation is discontinued and regulatory authorities have been notified. The Investigator must notify the Sponsor prior to destroying any clinical study records.

Should the Investigator wish to assign the study records to another party or move them to another location, the Sponsor must be notified in advance.

If the Investigator cannot guarantee this archiving requirement at the study site for any or all of the documents, special arrangements must be made between the Investigator and the Sponsor to store these in a sealed container(s) outside of the site so that they can be returned sealed to the Investigator in case of a regulatory audit. Where source documents are required for the continued care of the subject, appropriate copies should be made for storage outside of the site.

14.2.2 Background data

The Investigator shall supply the Sponsor, on request, with any required background data from the study documentation or clinic records. This is particularly important when errors in data transcription are suspected. In case of special problems and/or governmental queries or requests for audit inspections, it is also necessary to have access to the complete study records, provided that subject confidentiality is protected.

14.2.3 Audits and inspections

The Investigator should understand that source documents for this trial should be made available to appropriately qualified personnel from the Sponsor or its representative or to regulatory authority or health authority inspectors after appropriate notification. The verification of the CRF data may be by direct inspection of source documents (where permitted by law) or through an interview technique.

14.2.4 Case report forms

For each subject enrolled, CRFs must be completed and signed by the Principal Investigator or co-Investigator. This also applies to records for those subjects who fail to complete the study (even during a pre-randomisation screening period if a CRF was initiated). If a subject withdraws from the study, the reason must be noted on the CRF. If a subject is withdrawn from the study because of a treatment-limiting AE, thorough efforts should be made to clearly document the outcome.

All hardcopy forms should be typed or filled out using a black/blue pen, and must be legible. Errors should be crossed out but not obliterated, the correction inserted, and the change initialled and dated by the Investigator or his/her authorised delegate. The CRFs, as well as the protocol, are confidential. The CRFs remain the property of the Sponsor at all times.

14.3 Monitoring the study

In accordance with International Council on Harmonization Good Clinical Practice (ICH-GCP) guidelines, the study monitor must have direct access to the Investigator's source documentation in order to verify the data recorded in the CRFs for consistency.

It is understood that the responsible monitor as a representative of the Sponsor, will contact and visit the Investigator regularly and that they will be allowed, on request, to inspect the various records of the trial (CRFs and other pertinent data) provided that subject confidentiality is maintained in accord with local requirements.

It will be the monitor's responsibility to inspect the CRFs at regular intervals throughout the study, to verify the adherence to the protocol and the completeness, consistency and accuracy of the data being entered on them. Where local regulations permit, the monitor should have access to laboratory test reports and other subject records needed to verify the entries on the CRF. The Investigator agrees to cooperate with the monitor to ensure that any problems detected in the course of these monitoring visits are resolved.

14.4 Confidentiality of trial documents and subject records

The Investigator must assure the subjects' anonymity will be maintained and that their identities are protected from unauthorised parties. On CRFs or other documents submitted to the Sponsor, subjects should not be identified by their names, but by the subject's initials and an identification code. The Investigator should keep a subject enrolment log showing codes, names and addresses. Documents not for submission to the Sponsor (e.g. subject's written consent forms), should be maintained by the Investigator in strict confidence.

All information concerning the study treatment and the Sponsor and its operation, such as patent applications, formulae, manufacturing processes, basic scientific data and material not previously published are considered confidential and shall remain the sole property of the Sponsor. The Investigator agrees to use this information only in accomplishing the study and will not use it for any other purposes without written consent from the Sponsor.

14.5 Publication of data and protection of trade secrets

In accord with standard editorial and ethical practice, the Sponsor will support publication of multicentre trials only in their entirety and not as individual centre data.

The Sponsor will list the study on a public database listing of clinical trials, for example, the Australian and New Zealand Clinical Trials Registry (www.anzctr.org.au).

The results of this study may be published or presented at scientific meetings. If this is envisaged, the Investigator agrees to submit all manuscripts or abstracts to the Sponsor prior to submission. This allows the Sponsor to protect proprietary information and to provide comments based on information from other studies that may not yet be available to the Investigator.

Any formal publication of the study in which input of Sponsor personnel exceeded that of conventional monitoring will be considered as a joint publication by the Investigator and the appropriate Sponsor personnel. Authorship will be determined by mutual agreement prior to the start of the study. The number of subjects enrolled by respective sites will be taken into consideration in the selection of authors. Additional authors will be agreed prior to the completion of the study.

14.6 Anticipated subject accrual and duration of the study

The study will recruit and randomise 58 patients. The study will be conducted in two parts, Part A and Part B. A pre-screening study (HREC17/QPAH118) will support recruitment of patients into both Part A and Part B. The Sponsor has a target date for recruitment of all patients within 16 months of the site initiation visit. The Investigator should continually compare the actual and expected accrual rates, and make every effort to ensure that they are as closely matched as possible. If the Investigator anticipates major problems with recruitment, or delay in the expected completion date, he/she should discuss this with the Sponsor as early as possible.

15 REFERENCES

1 Goodson N, Marks J, Lunt M, et al. Cardiovascular admissions and mortality in an inception cohort of patients with rheumatoid arthritis with onset in the 1980s and 1990s. Ann Rheum Dis. 2005; **64**:1595-601.

2 Gregersen PK, Silver J, Winchester RJ. The shared epitope hypothesis: an approach to understanding the molecular genetics of suseptibility to rheumatoid arthritis. Arthritis Rheum. 1987; **30**:1205-13.

3 Raychaudhuri S, Sandor C, Stahl EA, et al. Five amino acids in three HLA proteins explain most of the association between MHC and seropositive rheumatoid arthritis. Nat Genet. 2012; **44**:291-96.

4 Han B, Diogo D, Eyre S, et al. Fine mapping seronegative and seropositive rheumatoid arthritis to shared and distinct HLA alleles by adjusting for the effects of heterogeneity. Am J Hum Genet. 2014; **94**:522-32.

5 Koning F, Thomas R, Rossjohn J, et al. Coeliac disease and rheumatoid arthritis: similar mechanisms, different antigens. Nat Rev Rheumatol. 2015; **11**:450-61.

6 Holmdahl R, Andersson EC, Andersen CB, et al. Transgenic mouse models of rheumatoid arthritis. Immunol Rev. 1999; **169**:161-73.

7 Rosloniec EF, Whittington KB, He X, et al. Collagen-induced arthritis mediated by HLA-DR1 (*0101) and HLA-DR4 (*0401). Am J Med Sci. 2004; **327**:169-79.

8 Rosloniec EF, Ivey RA, 3rd, Whittington KB, et al. Crystallographic structure of a rheumatoid arthritis MHC susceptibility allele, HLA-DR1 (DRB1*0101), complexed with the immunodominant determinant of human type II collagen. J Immunol. 2006; **177**:3884-92.

9 Snir O, Backlund J, Bostrom J, et al. Multifunctional T cell reactivity with native and glycosylated type II collagen in rheumatoid arthritis. Arthritis Rheum. 2012; **64**:2482-8.

10 Min SY, Hwang SY, Park KS, et al. Induction of IL-10-producing CD4+CD25+ T cells in animal model of collagen-induced arthritis by oral administration of type II collagen. Arthritis Res Ther. 2004; **6**:R213-9.

11 Min SY, Park KS, Cho ML, et al. Antigen-induced, tolerogenic CD11c+,CD11b+ dendritic cells are abundant in Peyer's patches during the induction of oral tolerance to type II collagen and suppress experimental collagen-induced arthritis. Arthritis Rheum. 2006; **54**:887-98.

12 Ning B, Wei J, Zhang A, et al. Antigen-specific tolerogenic dendritic cells ameliorate the severity of murine collagen-induced arthritis. PLoS One. 2015; **10**:e0131152.

13 Uysal H, Bockermann R, Nandakumar KS, et al. Structure and pathogenicity of antibodies specific for citrullinated collagen type II in experimental arthritis. J Exp Med. 2009; **206**:449-62.

14 Burkhardt H, Huffmeier U, Spriewald B, et al. Association between protein tyrosine phosphatase 22 variant R620W in conjunction with the HLA-DRB1 shared epitope and humoral autoimmunity to an immunodominant epitope of cartilage-specific type II collagen in early rheumatoid arthritis. Arthritis Rheum. 2006; **54**:82-9.

15 Benham H, Nel HJ, Law SC, et al. Citrullinated peptide dendritic cell immunotherapy in HLA risk genotype-positive rheumatoid arthritis patients. Sci Transl Med. 2015; **7**:290ra87.

16 van der Helm-van Mil AH. Risk estimation in rheumatoid arthritis: from bench to bedside. Nat Rev Rheumatol. 2014; **10**:171-80.

16 APPENDICES

16.1 Appendix 1 NCI CTCAE v4.03

See: https://evs.nci.nih.gov/ftp1/CTCAE/About.html

CTCAE Files

NCI Common Terminology Criteria for Adverse Events (CTCAE) v.4 data files and related documents are published here. The most current release files appear in this directory:

Files: Booklet	Content
CTCAE_4.03_2010-06-14_QuickReference_5x7.pdf	Most recent release of core terminology: PDF document, traditional small booklet format.
CTCAE_4.03_2010-06-14_QuickReference_8.5x11.pdf	Most recent release of core terminology: PDF document, letter size format.

Files: Data	Content
CTCAE_4.03_2010-06-14_Revisions.txt	Revision history with details.
CTCAE_4.03_2010-06-14.xls	Most recent release of core terminology in Excel spreadsheet.
CTCAE_4.03_2010-06-14.owl	Most recent release of complete terminology data in Web Ontology Language (OWL) format.

16.2 Appendix 2 DAS28CRPv4 Scoring Tool and Calculator

Vital Activities **DATE: _______________**

This questionnaire includes information not available from blood tests or any source other than you. Please try to answer each question even if you do not think it is related to you at this time. There are no right or wrong answers. Please answer exactly as you think or feel. Thank you.	

DATE OF FIRST RHEUMATOID ARTHRITIS SYMPTOMS:_________________________________

1. Please circle the ONE best answer for your abilities at this time:

AT THIS MOMENT, are you able to:

	Without ANY Difficulty	With SOME Difficulty	With MUCH Difficulty	UNABLE to do	MHAQ SCORE (total first 8 items)
a. Dress yourself, including tying shoelaces and doing buttons?	0	1	2	3	
b. Get in and out of bed?	0	1	2	3	
c. Lift a full cup or glass to your mouth?	0	1	2	3	
d. Walk outdoors on flat ground?	0	1	2	3	
e. Wash and dry your entire body?	0	1	2	3	
f. Bend down to pick up clothing from the floor?	0	1	2	3	
g. Turn regular taps on and off?	0	1	2	3	
h. Get in and out of a car or bus?	0	1	2	3	
i. Run errands and shop?	0	1	2	3	
j. Climb up a flight of stairs?	0	1	2	3	

k. Walk two kilometres?	0	1	2	3	
l. Run or jog two kilometres?	0	1	2	3	
m. Drive a car 10 kilometres from your home?	0	1	2	3	
n. Participate in sports and games as you would like?	0	1	2	3	
o. Get a good night's sleep?	0	1	2	3	
p. Deal with usual stresses of daily life?	0	1	2	3	
q. Deal with feelings of anxiety or being nervous?	0	1	2	3	
r. Deal with feelings of depression or feeling blue?	0	1	2	3	

2. How much pain have you had because of your condition OVER THE PAST WEEK? Place a mark on the line below to indicate how severe your pain has been. For example, if your pain was quite bad place a mark at this end:

No pain	○ 0	○ 5	○ 10	○ 15	○ 20	○ 25	○ 30	○ 35	○ 40	○ 45	○ 50	○ 55	○ 60	○ 65	○ 70	○ 75	○ 80	○ 85	○ 90	○ 95	○ 100	Pain as bad as can be

3. Considering all the ways in which illness and health conditions may affect you, please make a mark below to show how you are doing:

Not affected by illness	○ 0	○ 5	○ 10	○ 15	○ 20	○ 25	○ 30	○ 35	○ 40	○ 45	○ 50	○ 55	○ 60	○ 65	○ 70	○ 75	○ 80	○ 85	○ 90	○ 95	○ 100	Heavily affected by illness

4. When you get up in the morning do you feel stiff?

Yes ☐ ☐ No

5. If you answer "yes" how long is it until you are as limber as you will be for that day?

Please write the number of minutes:________

OR number of hours:________

Example Calculator

Enter clinical data to calculate the Disease Activity Score

DAS28-CRP Calculator

for the DAS28-CRP with 4 variables

by M. Flendrie and J. Fransen

Clinical variable	Value
tender joint count (0-28)	14
swollen joint count (0-28)	1
CRP (mg/L)	10
VAS general health patient (mm)	75

DAS28	5.25

Formula DAS28-4(crp) = 0.56*SQRT(TJC28) + 0.28SQRTSJC28) + 0.36*ln(CRP+1) + 0.014*GH + 0.96

Tender JC of 28		Patient disease activity of 100	
Swollen JC 0f 28		morning stiffness (min)	
mHAQ of 24			
Patient pain			

JOINT SCORES

Score open squares but do not include in tender and swollen joint counts. Score TMJ, cervical spine, hips, midtarsal and subtalar joints in tender count only. Count R/C and U/C as one joint.

Nodules: Yes / No

RIGHT **LEFT**

P P

T=tender (all visits)			**TMJ**				
S=swelling (all visits)			**Cervical spine**				
For Visits 0,12,24+36months:			**Sternoclavicular**				
D=deformed			**Manubriosternal**				
O=operation			**ACJ**				
L= lax	=r		**Shoulder**		r=		
r=give range or R for restricted	=r		**Elbow**		r=		
	=r		**Wrist**		r=		

5	4	3	2	1	**MCP**	1	2	3	4	5
5	4	3	2	1	**PIP**	1	2	3	4	5

					**DIP**					
					**Hip**					
			=r		**Knee**		r=			
			=r		**Ankle**		r=			
					**Subtalar**					
					**Mid tarsal**					
5	4	3	2	1	**MTP**	1	2	3	4	5

Cervical spine – pain on flexion/extension, score T Shoulder joint – pain on flexion/extension or internal/external rotation without abduction, score THip joint – pain on Flexion, Abduction and External Rotation (FABER), score T Subtalar and metatarsal joints – pain on inversion/eversion, score T R/C = radiocarpal joint; U/C = ulnar-carpal joint See EULAR hand book for more details

Adapted from Pincus T Arthritis Rheum 2003, Smolen JS Arthritis Rheum 1995
